# Genetic, biological and epidemiological study on a cluster of H9N2 avian influenza virus infections among chickens, a pet cat, and humans at a backyard farm in Guangxi, China

**DOI:** 10.1080/22221751.2022.2143282

**Published:** 2022-12-18

**Authors:** Jing Yang, Jianhua Yan, Cheng Zhang, Shanqin Li, Manhua Yuan, Chunge Zhang, Chenguang Shen, Yang Yang, Lifeng Fu, Guanlong Xu, Weifeng Shi, Zhenghai Ma, Ting Rong Luo, Yuhai Bi

**Affiliations:** a CAS Key Laboratory of Pathogen Microbiology and Immunology, Institute of Microbiology, Center for Influenza Research and Early-warning (CASCIRE), CAS-TWAS Center of Excellence for Emerging Infectious Diseases (CEEID), Chinese Academy of Sciences, Beijing, People’s Republic of China; b Laboratory of Animal Infectious Diseases, Medical College & College of Animal Sciences and Veterinary Medicine, Guangxi University, Nanning, People’s Republic of China; c College of Life Science and Technology, Xinjiang University, Urumchi, People’s Republic of China; d Shenzhen Key Laboratory of Pathogen and Immunity, Guangdong Key Laboratory for Diagnosis and Treatment of Emerging Infectious Diseases, State Key Discipline of Infectious Disease, Second Hospital Affiliated to Southern University of Science and Technology, Shenzhen Third People’s Hospital, Shenzhen, People’s Republic of China; e School of Public Health, Southern Medical University, Guangzhou, People’s Republic of China; f China Institute of Veterinary Drug Control, Beijing, People’s Republic of China; g Key Laboratory of Etiology and Epidemiology of Emerging Infectious Diseases in Universities of Shandong, Shandong First Medical University & Shandong Academy of Medical Sciences, Taian, People’s Republic of China; h University of Chinese Academy of Sciences, Beijing, People’s Republic of China

**Keywords:** H9N2, avian influenza virus, interspecies transmission, genetic evolution, public health risk

## Abstract

During an investigation in October 2018, two people with diarrhoea, mild abdominal pain, and mild arthralgia symptoms in Guangxi, China, were identified as infected by H9N2 avian influenza virus (AIV). Four H9N2 AIVs were isolated from one of two patients, a pet cat, and a dead chicken (two respective isolates from its lung and kidney tissues) bred by the patients at a backyard farm. Epidemiological investigation indicated that the newly bought chicken died first, and clinical syndromes appeared subsequently in the two owners and one cat. Furthermore, the two individuals possessed high H9N2-specific hemagglutination inhibition and microneutralization antibodies. Shared nucleotide sequence identity (99.9% – 100%) for all genes was detected in the four H9N2 isolates, and hemagglutinin (HA) T138A located on the receptor binding domain (RBD), resulted from nucleotide polymorphisms that were exclusively found in the isolate from the female patient. Moreover, HA K137N on the RBD was found in isolates from these three host species. Importantly, these four H9N2 isolates presented an exclusive binding preference for the human-type receptor (α2-6-SA), and could replicate and cause pathological changes in mice. Phylogenetic analyses showed that these four isolates clustered together and belonged to clade C1.2, lineage Y280. In addition, H9N2 viruses of human origin are genetically divergent and interspersed with the widespread poultry-origin H9N2 AIVs. All these results indicate a high risk of H9N2 AIVs in public health, and effective prevention and control measures against H9N2 AIVs should be considered and performed for both animal and human health.

## Introduction

The A/H9N2 (hereafter referred to as H9N2) low-pathogenicity avian influenza virus (LPAIV) is of great global concern since its worldwide circulation and even long-term endemicity in the poultry populations of countries in Asia, Africa, and the Middle East [1–5]. Based on nationwide surveillance in 2016–2019 across mainland China, the H9N2 virus has become the dominant influenza subtype persisting in both chickens and ducks [1]. Since H9N2 AIV was firstly isolated in mainland China in 1994, diverse lineages and genotypes of the virus have been established through extensive and complicated genetic reassortment and mutations [1,3,6–7]. Moreover, increasing H9N2 isolates display predominant human-type receptor specificity characterized by a Leu (L) residue at amino acid position 226 (numbered by H3) in the receptor binding domain (RBD) of the hemagglutinin (HA) protein [1,8]. The widespread persistence, cocirculation of divergent variants, and growing mutations associated with the human-type receptor preference of H9N2 virus in birds increase the public health risk of human infections.

Occasional cross-species transmission of H9N2 AIVs from poultry to mammals, especially humans, has been reported [4,9–11]. Since the first report of a human case of H9N2 virus in 1998, at least 110 human infections have been confirmed in China, Bangladesh, Egypt, Pakistan, Oman, India, Senegal, and Cambodia as of May 2, 2022, including 97 cases documented in China (https://www.who.int/teams/global-influenza-programme/avian-influenza/monthly-risk-assessment-summary) [12–14]. Typically, H9N2-infected patients present with mild influenza-like symptoms; hence, many human H9N2 infections may be undetected, which is supported by serological investigations in poultry-associated workers (https://www.who.int/teams/global-influenza-programme/avian-influenza/monthly-risk-assessment-summary) [15,16]. Furthermore, H9N2 AIVs exchange their genes with other influenza subtypes to generate novel viruses (e.g. A/H7N9, A/H10N8, A/H10N3, and A/H5N6), which have caused human infections [2,17–19]. The novel A/H7N9 AIV contains six internal protein-coding genes from the H9N2 virus circulating in poultry, resulting in five epidemic waves in humans in mainland China since 2013 [20–22]. The fact that humans were directly infected by H9N2 and even by other novel subtype AIVs with H9N2 internal-protein coding genes further highlights the threat of H9N2 to public health.

In the present study, we report a cluster of H9N2 infections among two humans, one pet cat, and several chickens at a backyard farm in Guangxi Province in October 2018. An epidemiological investigation and hemagglutination inhibition (HI) and microneutralization (MN) assays were performed to determine the disease progression of H9N2 human infections on the farm. Molecular characteristics analyses and the receptor-binding and virulence-related assays were also employed to understand the infective features and to explore the potential transmission routes of the H9N2 virus among chickens, humans, and cats. The findings of our study further uncover the public health risks of H9N2 and support the development of targeted prevention and control strategies.

## Materials and methods

### Ethics statement

Animal studies were conducted according to the recommendations of the Guide for the Care and Use of Laboratory Animals of the Ministry of Science and Technology, China. In this study, the animal experiments were approved by the Research Ethics Committee of the Institute of Microbiology, Chinese Academy of Sciences (APIMCAS2018014).

### Eggs

Ten-day-old specific pathogen-free (SPF) embryonated chicken eggs were obtained from Beijing Vital River Laboratory Animal Technology Company (China) and used for virus isolation at 37 °C and 80% humidity.

### Sample collection and virus isolation

Lung and kidney samples of the dead chicken were collected on October 15, 2018. Throat swabs from the two patients were collected on October 19. On the same day, the diseased cat was euthanized, and heart, liver, spleen, kidney, lung, and larynx samples were collected. The supernatants of the swabs and tissue homogenates were inoculated into 10-day-old specific pathogen-free chicken embryos through the allantoic cavity inoculation route, as previously described [1,2]. After 1–3 consecutive passages, the hemagglutination titre-positive allantoic fluids were further tested using quantitative reverse transcriptase PCR (qRT-PCR) kits (Mabsky Biotech, China) to identify and subtype the influenza viruses.

### Whole-genome sequencing and assembly of H9N2 AIVs

Viral RNA was extracted from the allantoic fluid of the AIV-positive samples, including throat samples from the female patient, heart samples from the cat, and lung and kidney samples of the dead chicken, using the MagaBio plus Virus RNA Purification Kit (BIOER, China). The whole genome of the AIV isolates was sequenced using next-generation sequencing (NGS). Specifically, viral RNAs were used for RT-PCR, and DNA synthesis was conducted as previously described [1,2]. Sequencing libraries were then prepared. The libraries were sequenced using paired-end sequencing with 150 bp per read and ∼0.2G sequencing data per sample using the Illumina NovaSeq6000 PE150 sequencer (Illumina, USA).

Sequencing reads generated by NGS were processed and assembled to obtain sequence information on H9N2 AIVs. First, the low-quality reads (> 50% bases with quality ≤ 19), adaptor-contaminated reads (> 5 bp matched to the adaptor sequence in either of the paired reads), poly-Ns (with 8Ns), and duplicated reads were discarded from the raw read data using SOAP2 v2.21 [23]. The clean reads were mapped according to influenza sequence data stored in the INFLUENZA database (downloaded on June 1, 2018) to select the best-matching reference sequences [24]. Later, reference-based sequencing assembly was performed using BWA v0.7.12 and SAMtools v1.4 [25,26]. In addition, the proportions of the four nucleotides at each position in each gene sequence were calculated. The consensus sequences generated using NGS sequencing method were cross-validated using the Sanger sequencing.

The four H9N2 isolates were named A/Guangxi/NN10.19T-NGS/2018, A/cat/Guangxi/NN10.19H-NGS/2018, A/chicken/Guangxi/NN10.19L1-NGS/2018, and A/chicken/Guangxi/NN10.19K2-NGS/2018. The corresponding gene sequences obtained in this study are deposited in the National Microbiology Data Center (https://nmdc.cn; accession number: NMDCN0001181 – NMDCN0001190) and the EpiFlu database of GISAID (https://www.gisaid.org; accession number: EPI1856269 – EPI1856300).

### Receptor binding assay

The receptor-binding specificity of three H9N2 AIV isolates from the patient, cat, and chicken was detected through a solid-phase direct binding assay using the biotinylated glycans α2,3 sialic acid receptor (α2-3-SA; Neu5Acα2-3Galβ1-4GlcNAcβ-SpNH-LC-LC-Biotin) and α2,6 sialic acid receptor (α2-6-SA; Neu5Acα2-6Galβ1-4GlcNAcβ-SpNH-LC-LC-Biotin) as described previously [1]. Briefly, viral dilutions containing 32 or 64 HA units with the neuraminidase inhibitors (NAIs; 10μM each of oseltamivir and zanamivir) were incubated. Virus-receptor binding properties were detected with rabbit antisera against the influenza virus H9, H5, or H1 and the HRP-linked goat-anti-rabbit antibody (Bioeasytech, China). A/Vietnam/1194/2004(H5N1) and A/California/04/2009(H1N1) were used as controls in this assay.

To further confirm virus receptor-binding specificity, we performed hemagglutination assays according to the manual from the World Health Organization (WHO) using 1% chicken red blood cells (cRBCs, expressing both α2-3-SA and α2-6-SA receptors), 1% resialylated cRBCs (α2-6-SA receptors), and 1% sheep red blood cells (sRBCs, α2-3-SA receptors) [27,28]. The resialylated cRBCs were generated by treating cRBCs with 2,3-sialidase (Takara Bio (Beijing), China) to remove the α2-3-SA receptors from the cRBCs. Specifically, we added 10 µl 2,3-sialidase (with 50 mu/µl concentration) to 90 µl 10% cRBCs suspension, mixed gently, and incubated them for 15 min at 37 °C. Then, the treated cRBCs were washed twice by phosphate buffer saline (PBS) and centrifuged at 400 × g for 5 min each time. The sediments were used to prepare the 1% resialylated cRBCs by PBS.

### Hemagglutination inhibition and microneutralization antibody detection

Plasma samples collected from the two patients were treated with the receptor-destroying enzyme (RDE). We tested the plasma samples for antibodies against the H9N2 isolate (A/Guangxi/NN10.19T-NGS/2018), and representatives of influenza A virus, namely, A/H1N1 (A/Brisbane/02/2018), A/H3N2 (A/Kansas/14/2017), and A/H5N6 (A/Shenzhen/TH002/2015), as well as influenza B viruses, B/Colorado/06/2017 (belonging to B/Victoria/2/87 lineage labelled as Bv hereafter) and B/Phuket/3073/2013 (belonging to B/Yamagata/16/88 lineage labelled as By hereafter), using HI and MN assays, respectively, according to the manual provided by the WHO [28] and a previous report [16]. Seasonal influenza A/H1N1, A/H3N2, Bv, and By strains were kindly provided by Dr. Dayan Wang and Dr. William J. Liu (Chinese Center for Disease Control and Prevention, China). We considered ≥ 1:40 and ≥ 1:80 as the cut-off values for the HI and MN titres for H9N2 positivity, respectively [16,29].

### Mouse pathogenicity experiments

Four groups of fourteen 7- to 8-week-old female BALB/c mice (Beijing Huafukang Bioscience, China) were used to examine the pathogenicity of H9N2 viruses identified in this study. Three groups of BALB/c mice were intranasally inoculated with 10^6^ EID_50_ of H9N2 viruses isolated from the chicken (A/chicken/Guangxi/NN10.19L1-NGS/2018), cat (A/cat/Guangxi/NN10.19H-NGS/2018), and human (A/Guangxi/NN10.19T-NGS/2018), respectively. One control group of mice was intranasally inoculated with PBS. Three mice in each group were euthanized on 3, 5, and 7 days post-infection (dpi). The lungs of the euthanized mice were harvested and homogenized to detect viral titres (viral RNA copies) using qRT-PCR. The lung index (ratio of lung weight to body weight) was also calculated for each euthanized mouse. In addition, five mice in each group were monitored for bodyweight changes until 14 dpi. The percent bodyweight change was calculated as the ratio of bodyweight to initial bodyweight on day 0.

For histopathological examinations, the left lungs collected from euthanized mice at 5 dpi were immersed in 4% paraformaldehyde. The fixed lung tissues were dehydrated, embedded in paraffin, cut into 4-μm-thick sections, and stained with haematoxylin and eosin (H&E) staining.

### Molecular characteristic analyses

To uncover the genetic divergence of H9N2 viruses isolated from the chicken, cat, and human at the backyard farm, we first calculated the percent identity between each H9N2 virus based on consensus nucleotide sequences. Next, we analysed the single nucleotide polymorphisms (SNPs) and amino acid substitutions resulting from the nucleotide polymorphisms in H9N2 virus populations isolated from the different hosts. We also analysed the key amino acid sites in the HA, NA, PB2, PA, and M genes to understand the potential biological features of H9N2 viruses, including the receptor-binding preference, replication, virulence, and antiviral drug susceptibility. Subsequently, we inferred the structure of the ectodomain of HA molecule of A/Guangxi/NN10.19T-NGS/2018, using the homology modelling method with A/swine/Hong Kong/9/98 (H9N2; PDB ID: 1JSD) as a template in the SWISS-model [30]. The three-dimensional protein structure was annotated and visualized using VMD v1.9.4 [31].

### Phylogenetic analysis

The nucleotide sequences of eight gene segments of H9N2 AIVs isolated in mainland China were downloaded from the influenza virus database hosted by the National Center for Biotechnology Information (NCBI; https://www.ncbi.nlm.nih.gov/genomes/FLU/Database/nph-select.cgi#mainform) and the EpiFlu database of the GISAID as of November 22, 2020. Additionally, we downloaded the nucleotide sequences of H9N2 AIVs isolated from humans in China. Based on previous studies, we also gathered the representative strains for H9N2 lineage classification: the BJ/94 lineage (represented by A/chicken/Beijing/1/1994), Y280 lineage (A/duck/Hong Kong/Y280/1997), G1 lineage (A/quail/Hong Kong/G1/1997), and Y439 lineage (A/duck/Hong Kong/Y439/1997) [3,6]. We removed sequences that were less than 90% full-length and retained one of the duplicated sequences based on the sequence names. We integrated sequences of H9N2 AIVs generated in this study, data obtained from public databases, and representative sequences for subsequent analyses. We then aligned the sequences of each gene using the default parameters in MAFFT v7.453 [32]. We constructed the phylogenetic trees of eight genes, except the HA gene of the virus, using maximum likelihood inference with the best-fit model automatically selected by ModelFinder and 1000 ultrafast bootstrap replicates using IQ-TREE v2.0.3 [33]. Limited by the computational complexity of a larger dataset of H9N2 HA sequences, we used RAxML v8.2.12 to infer the maximum-likelihood phylogeny of the H9N2 HA gene using the GTRGAMMA nucleotide substitution model and 100 bootstrap replicates [34]. To understand the evolutionary position and phylogenetic relationship of H9N2 AIVs in the present study, we used the clade classification method reported in a previous study to classify our H9N2 genes [1]. More details of the sequences used in this study are provided in the Supplementary Material (Table S1).

### Epidemiological data collection

During our surveillance on AIVs, two farmers presented some syndromes who bred chickens in their backyard, so we collected samples from them and their cats and chickens for the identification of pathogens. And we collected demographic characteristics (including age, gender, and occupation) of the two humans living on the farm, and the geographic location. We also recorded the clinical symptoms, onset dates, course of disease development in the humans, cat, and chickens and their exposure and contact history.

## Results

### Epidemiological investigation

**Figure 1.**
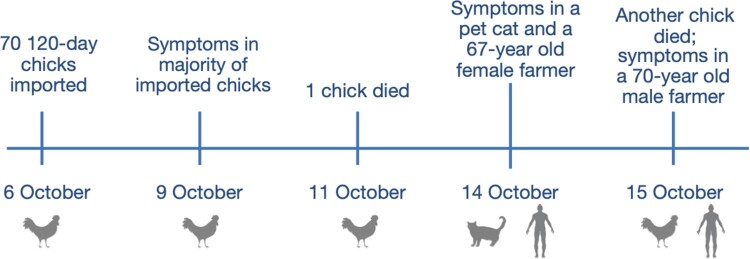
Schematic representation of the timeline of H9N2 AIV infections among chickens, a pet cat, and two humans at a backyard farm in Guangxi Province, October 2018.

On October 6, 2018, seventy 120-day-old chicks were imported into a backyard farm that already had two chickens (Figure 1). Three days later, the majority of the newly bought chickens showed clinical symptoms, whereas the two chickens originally raised on the farm were still healthy. One newly imported chick died on October 11 and another died on October 15, after which the other animals were slaughtered by the owners. On the morning of October 14, inappetence and nausea were observed in one of the three pet cats kept on the farm. Further, one of the two farmers (a 67-year-old female) living on the farm had slight pains in her abdomen and hand joints on the night of October 14, and experienced three episodes of diarrhoea on October 15, which self-healed later. Another farmer (a 70-year-old male) had a slight pain in his abdomen on October 15, which self-healed later.

### Identification of the causative agent H9N2 AIV and the HI and MN antibodies in the two patients

The H9N2 AIVs were isolated from the throat swab of the female patient, heart sample from the pet cat, and lung and kidney samples from the dead chicken after 1–3 consecutive passages.

**Figure 2.**
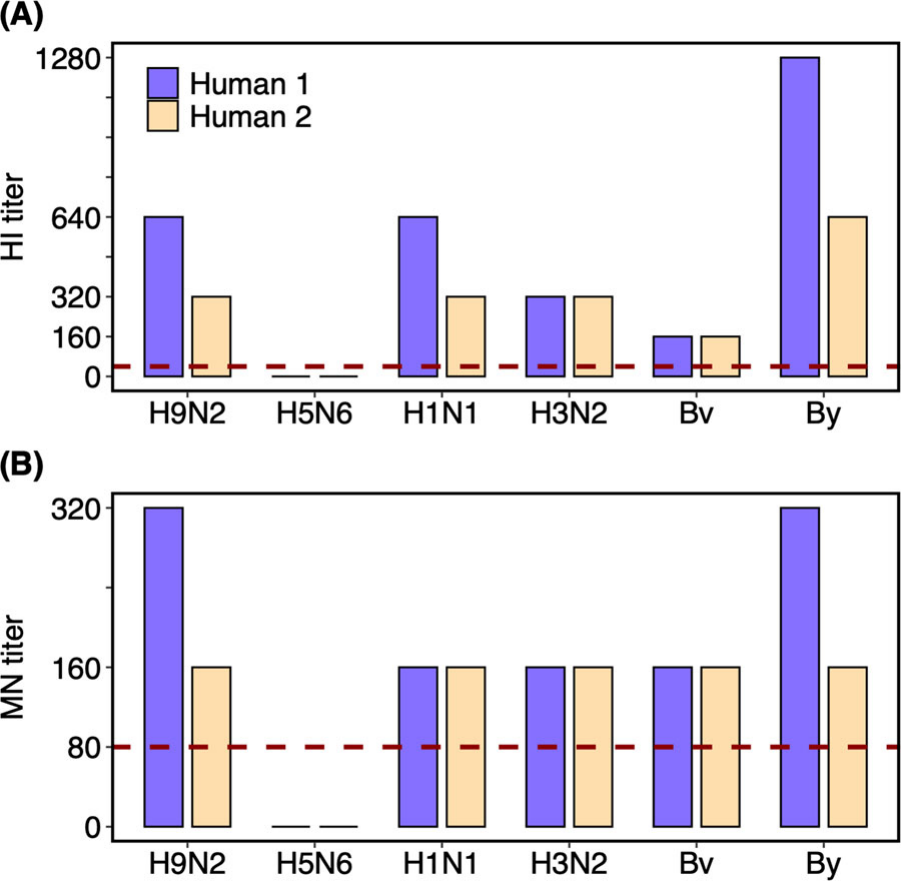
**HI and MN titres against different influenza viruses of the plasma samples from H9N2 infected couple.** Plasma samples of the two recovered humans were collected on the 25th day after the symptom onset. Dashed lines labelled the 1:40 and 1:80 as the low limits for the positive (A) HI and (B) MN titres, respectively. Human 1 and 2 represent the female and male patients, respectively. Virus isolates used in the assay including A/Guangxi/NN10.19T-NGS/2018(H9N2), A/Shenzhen/TH002/2015(H5N6), A/Brisbane/02/2018(pH1N1), A/Kansas/14/2017(H3N2), B/Colorado/06/2017(Bv), and B/Phuket/3073/2013(By).

The levels of HI and MN antibodies against the H9N2 and other influenza viruses (including A/H5N6 virus and seasonal influenza viruses A/H1N1, A/H3N2, Bv, and By) in the plasma samples of the two recovered humans were detected (Figure 2). The plasma samples from the patients presented high HI and MN titres against the H9N2 isolate, with 1:640 HI titre and 1:320 MN titre for the female and 1:320 HI titre and 1:160 MN titre for the male. However, antibodies against A/H5N6 virus were negative. Moreover, HI and MN titres of 160–1280 and 160–320, respectively, were detected for the seasonal influenza viruses in both patients. Specifically, the HI titres against A/H1N1 (1:640, 1:320), A/H3N2 (1:320, 1:320), Bv (1:160, 1:160), and By (1:1280, 1:640) were detected in plasma samples from the female and male patients, and the corresponding MN titres against A/H1N1 (1:160, 1:160), A/H3N2 (1:160, 1:160), Bv (1:160, 1:160), and By (1:320, 1:160) were also detected. The HI and MN titres against seasonal influenza detected in plasma samples from the two humans could have been induced by natural infections or vaccination.

### Receptor-binding properties of H9N2 AIVs

**Figure 3.**
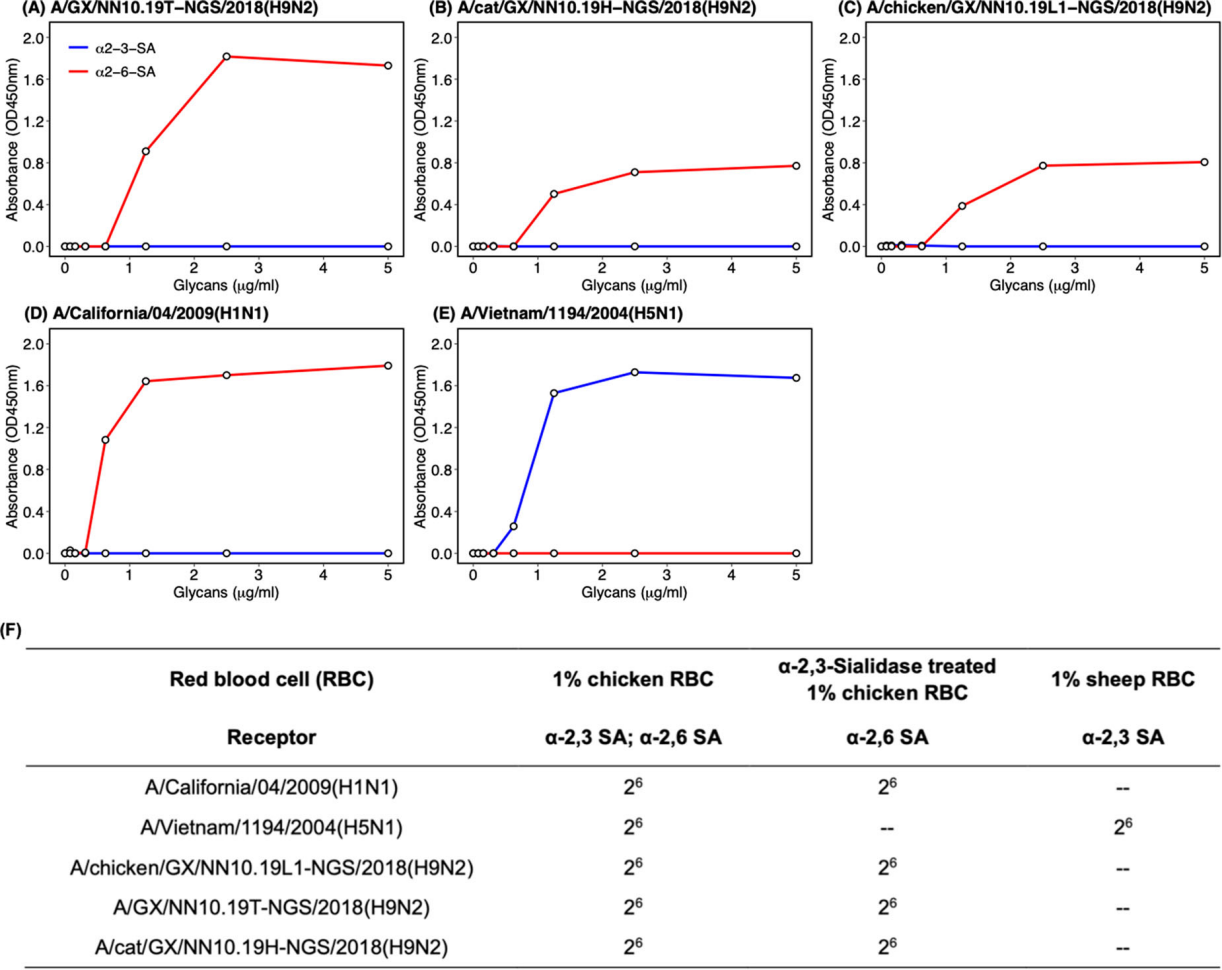
**Receptor-binding properties of H9N2 AIVs isolated from the patient, cat, and chicken.** Receptor-binding properties of H9N2 isolated from (A) the human, (B) cat, (C) chicken, and (D) A/Vietnam/1194/2004(H5N1) and (E) A/California/04/2009(H1N1) to human-type (α2-6-SA) and avian-type (α2-3-SA) receptors were detected by the solid-phase direct binding assay with the trisaccharide receptors. The human-type and avian-type receptors are colored by red and blue, respectively. The mean of values obtained by reading the absorbance three times was shown by points. (F) Receptor-binding properties of H9N2, H1N1 and H5N1 viruses were further confirmed by hemagglutination assays using red blood cells expressing different sialic acid receptors. GX represents Guangxi Province in sequence names. – represents negative.

To understand the potential mechanism behind the interspecies transmission of H9N2 AIVs on the same farm, we performed solid-phase receptor-binding detection and hemagglutination assay using the red blood cells with different types of sialic acid receptors to test the receptor-binding properties of the H9N2 isolates. In the solid-phase receptor-binding assay, the H9N2 viruses isolated from the human, cat, and chicken showed an exclusive binding preference for the human-type receptor (α2-6-SA) rather than the avian-type receptor (α2-3-SA) (Figure 3). The same results were also found in the hemagglutination assay. These H9N2 viruses can agglutinate cRBCs (α2-6-SA and α2-3-SA) and resialylated cRBCs (α2-6-SA) rather than the sRBCs (α2-3-SA) (Figure 3). In the control group, the A/H5N1 only bound to the avian-type receptor and agglutinated RBCs with α2-3-SA receptors, whereas the A/H1N1 isolate only bound to the human-type receptor and agglutinated RBCs with α2-6-SA receptors (Figure 3).

### Pathogenicity of H9N2 AIVs in mouse model

**Figure 4.**
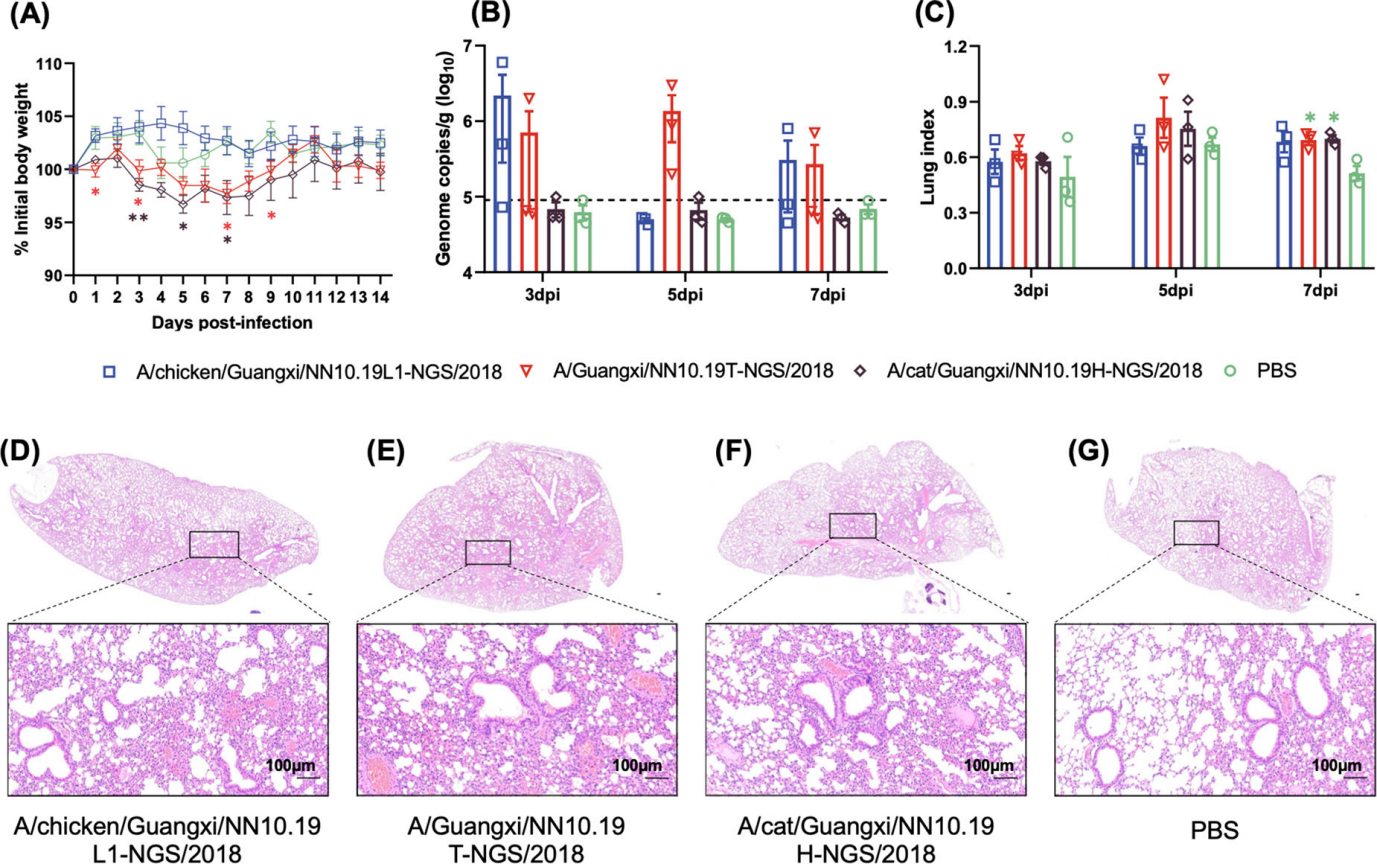
**Pathogenicity of the H9N2 viruses from chicken, cat, and human in BALB/c mice.** (A) Body weight changes of BALB/c mice inoculated with H9N2 viruses. (B) Lung viral titres (viral RNA copies) of infected mice were detected by qRT-PCR on 3, 5 and 7 days post-infection (dpi). The horizontal dashed line represents the lower limit of detection. (C) Lung index of the infected mice on 3, 5 and 7 dpi. Asterisk represents the statistically significant difference between the corresponding group and the control group given the Student’s T-test. *, *p*-value < 0.05; **, *p*-value < 0.01. (D)-(G) H&E staining results for the lung tissues of these H9N2 infected mice. The bar is 100 μm.

Mice infected with H9N2 viruses isolated from the cat and human showed non-negligible body weight losses from 3 dpi to 9 dpi compared to mice inoculated with PBS or H9N2 virus from the chicken (Figure 4). The lung viral titres of mice infected with H9N2 viruses from the human and chicken seem higher than those of mice infected with PBS and the H9N2 virus from cat; however, there was no statistical significance in viral titres between these groups, and the viral titres are relatively dispersed for each of the former two groups. The lung index values of mice infected with these H9N2 viruses were significantly (*p*-value < 0.05 given Student’s T-test) higher than those of mice infected with PBS in the control group at 7 dpi. H&E staining revealed the pathological changes in the lungs (e.g. inflammatory cell infiltration, hemorrhage, and thickening alveolar wall) of mice challenged with the H9N2 viruses.

### Molecular characteristics of H9N2 AIVs

Considering the consensus nucleotide sequences, the percent sequence identities between each pair of H9N2 AIVs isolated in this study were over 99.9% for each of the eight genes: HA (99.94% – 100%), NA (99.93% – 100%), PB2 (99.96% – 100%), PB1 (100%), PA (99.95% – 100%), MP (99.90% – 100%), NP (100%), and NS (100%).

**Figure 5.**
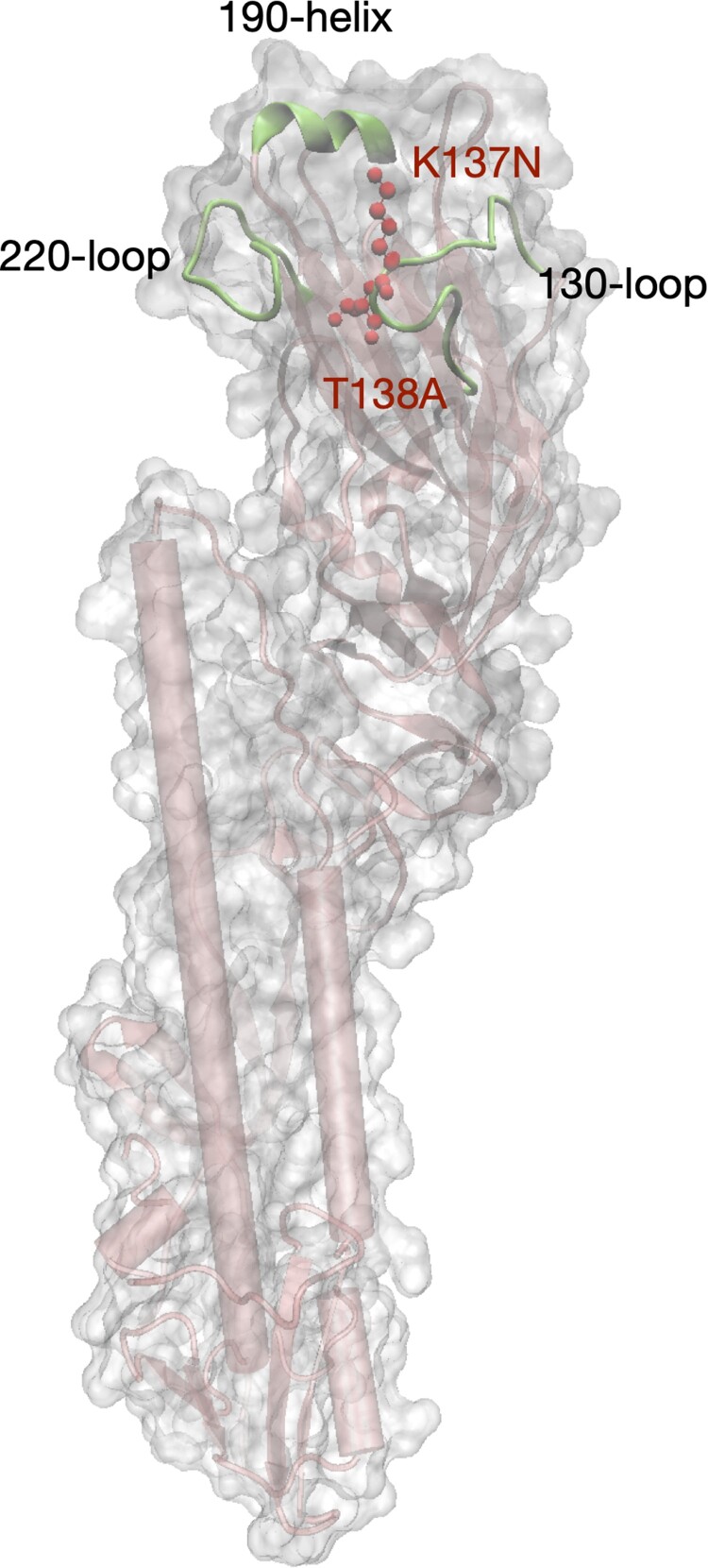
**Modeling structure of the ectodomain of HA molecule of the human H9N2 isolate.** The structure was modelled by a homology modelling method using A/swine/Hong Kong/9/98 as a template (PDB ID: 1JSD) in SWISS-model. K137N and T138A (H3 numbering) in the receptor binding domain (RBD) were labeled in red color. The RBD includes three secondary elements, 130-loop, 190-helix, and 220-loop, forming the edge of RBD, and four conserved residues, 98Y, 153W, 183H, and 195Y which form the base element.

To further uncover the genetic diversity of the virus populations, we found ten nucleotide polymorphisms specific to H9N2 isolated from the human and cat in comparison with those from the chicken given the NGS data (Table 1). Three of ten nucleotide polymorphisms resulted in two amino acid substitutions in the human isolate (HA T138A and PB1 Y657stop codon) and one in the cat isolate (NS1 A202D). The HA T138A of the human H9N2 virus is located on the 130-loop of the RBD according to the protein structure of the HA molecule of human H9N2 (Figure 5). The K137N substitution in the RBD of HA caused by nucleotide polymorphisms also occurs in H9N2 viruses from the human, the cat, and a lung sample of the chicken. The amino acid mutation from Y (87.02%) to the stop codon (11.40%) at amino acid position 657 truncated the PB1 protein of the human isolate (Table 1 and Table S2).

**Table 1.** Amino acid substitutions resulted from SNPs specific for H9N2 AIVs isolated from the human and cat in comparison with two others from chicken.

Virus host		Human and cat			Chicken
Gene	Position ^a^	Amino acid percent ^b^ (aa (codon): percent)	Host	Type of mutation^c^	Consensus amino acid^d^
HA	138	T(ACA): 85.34, A(GCA): 14.41	Human	NS	T
HA	475	D(GAC): 90.34, D(GAT): 9.36	Human	S	D
HA	475	D(GAC): 93.88, D(GAT): 5.89	Cat	S	D
NA	13	V(GTT): 58.21, V(GTC): 41.07	Cat	S	V
PB2	50	M(ATG): 84.87, M(CTG): 9.40	Cat	S	M
PB1	657	Y(TAT): 87.02, *(TAA): 11.40	Human	NS	Y
MP1	230	K(AAG): 84.93, K(AAA): 14.42	Cat	S	K
NP	271	A(GCC): 84.51, A(GCT): 15.11	Cat	S	A
NS1	139	N(AAT): 84.56, N(AAC): 14.82	Human	S	N
NS1	202	A(GCT): 84.24, D(GAT): 15.18	Cat	NS	A

^a^ The amino acid position in HA and NA of H9N2 was based on H3 and N2 numbering, respectively.

^b^The proportion of the amino acid residues on the SNP sites of the four H9N2 isolates. The read depths of NGS sequencing for nucleotides in each codon are shown in Table S2.

^c^NS and S represent non-synonymous and synonymous substitutions, respectively.

^d^ Consensus amino acid information from two H9N2 isolates identified from the chicken.

Furthermore, all four H9N2 AIVs isolated in this study possessed 226L, 155 T, and 183N in the HA gene sequences (H3 numbering; Table 2), consistent with their preference for human-type receptors (Figure 3). Four H9N2 HA genes also possessed PSRSSRGLF at cleavage sites, which is the molecular feature of LPAIV. A 3-amino acid (aa) deletion at positions 62–64 (H3N2-N2 numbering) in the NA stalk region was observed in the four H9N2 isolates, suggesting better adaptation to terrestrial birds [35–37]. Moreover, 591Q, 627E, and 701D in PB2 indicated that the four H9N2 isolates had not yet adapted to mammals [38–42], but the PB2 A588V substitution may increase mammalian adaptation [43]. In our four H9N2 isolates, 28P and 63I in PA and 31N in M2 gene were observed, suggesting that these isolates are sensitive to baloxavir [1,44] but resistant to amantadine and rimantadine [45].

**Table 2.** Molecular characterization of four H9N2 AIVs in this study.

Isolate name	HA (Receptor binding sites, H3 numbering)										HA cleavage sites	N2 stalk deletion(N2 numbering)	PB2 (Enhance virus replication or virulence to mammals)					PA (Affect baloxavir acid (BXA) susceptibility)		M2
	135	138	155	158–160	173	183	186	189–190	221–222	226–228			526	588	591	627	701	28	63	31
A/cat/Guangxi/NN10.19H-NGS/2018	T	T	T	DGN	G	N	*P*	DT	PL	LMG	PSRSSRGLF	62–64	K	V	Q	E	D	*P*	I	N
A/chicken/Guangxi/NN10.19L1-NGS/2018	T	T	T	DGN	G	N	*P*	DT	PL	LMG	PSRSSRGLF	62–64	K	V	Q	E	D	*P*	I	N
A/chicken/Guangxi/NN10.19K2-NGS/2018	T	T	T	DGN	G	N	*P*	DT	PL	LMG	PSRSSRGLF	62–64	K	V	Q	E	D	*P*	I	N
A/Guangxi/NN10.19T-NGS/2018	T	T	T	DGN	G	N	*P*	DT	PL	LMG	PSRSSRGLF	62–64	K	V	Q	E	D	*P*	I	N

### Phylogeny of H9N2 isolates

**Figure 6.**
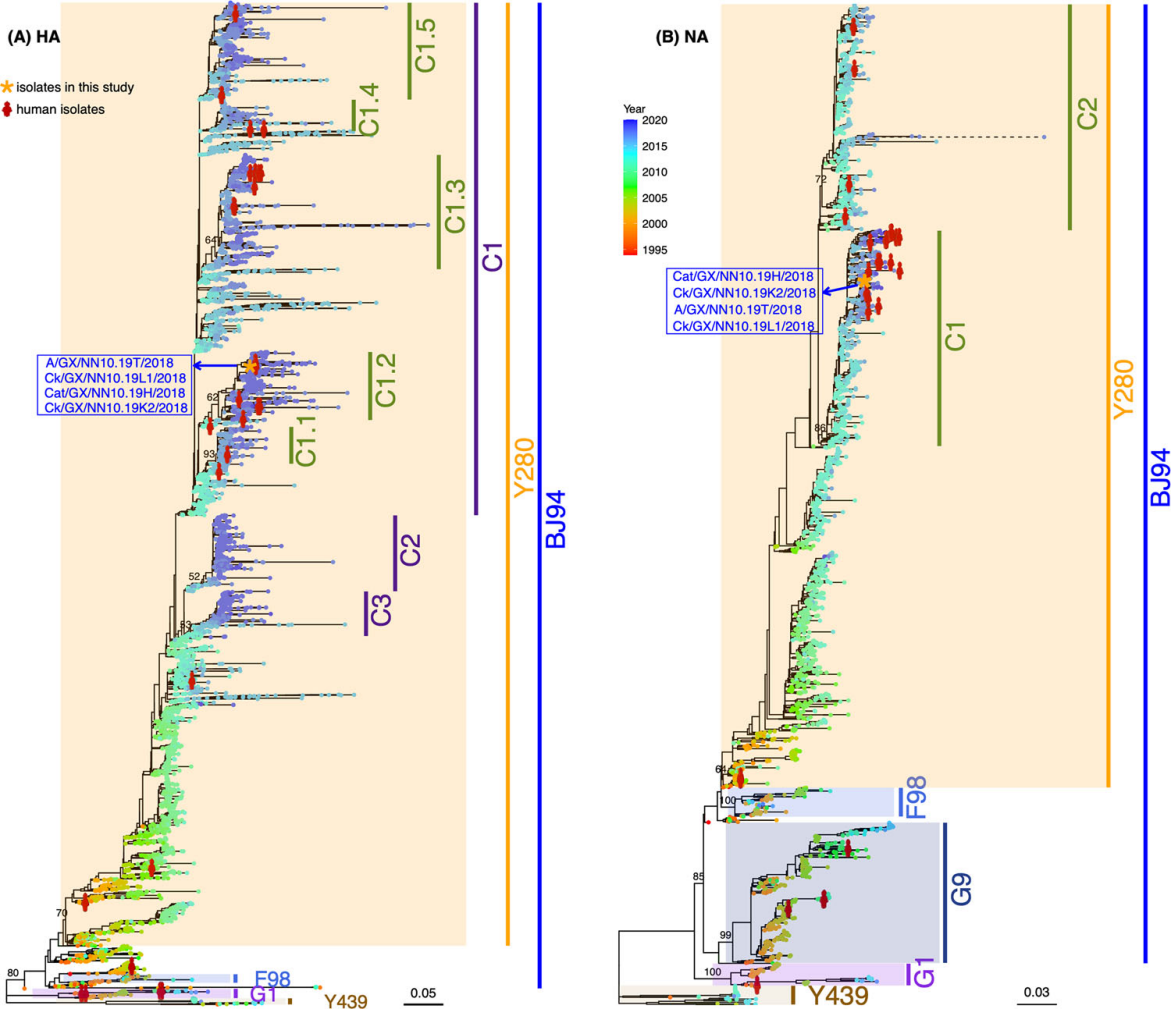
**Phylogenetic trees of HA and NA genes of H9N2 AIVs in mainland China.** H9N2 viruses isolated in this study are marked by orange asterisks and their sequence names are listed next to the asterisks in blue color. Human H9N2 from public databases are labeled by red stick figures of humans. Tips of each tree were colored by the isolation year of the corresponding tip taxa. Bars on the right represent multiple established lineages and clades given tree topology and genetic relationship. Numbers on the branches represent the support of corresponding clades.

The four H9N2 AIVs isolated in this study fell into the same phylogenetic clade, given the evolutionary relationship of eight genes of H9N2 viruses circulating in mainland China (Table 3, Figure 6, and Figure S1). The HA sequences of four H9N2 AIVs collected in the present study were clustered in clade C1.2 of the Y280 lineage represented by A/duck/Hong Kong/Y280/1997. The H9N2 NA genes belonged to clade C1 of the Y280 lineage. The PB1, PA, NP, and NS genes of the four H9N2 isolates belonged to the F98 lineage represented by A/chicken/Shanghai/F/1998 (Figure S1). The MP and PB2 genes belonged to the G1 lineage represented by A/quail/Hong Kong/G1/1997 and the SH5 lineage represented by A/chicken/Shanghai/5/2003, respectively (Figure S1). PB2-SH5 is a new lineage proposed in this study, given its genetic divergence from previous lineages identified by H9N2 representatives. The established lineages and clades, including our four viruses (HA-C1, NA-C1, PB2-SH5, PB1-F98, PA-F98, MP-G1, NS-F98, and NP-F98), have been the dominant lineages of H9N2 persisting in mainland China, especially in recent years (Figure 6, Table 3). Furthermore, all 35 H9N2 viruses isolated from humans were divergently distributed in the phylogenetic trees, rather than forming a clustered pattern.

**Table 3.** Genetic source of eight gene segments of H9N2 AIVs in this study

Isolate name	HA	NA	PB2	PB1	PA	MP	NP	NS
A/cat/Guangxi/NN10.19H-NGS/2018	Y280-C1.2	Y280-C1	SH5	F98	F98	G1	F98	F98
A/chicken/Guangxi/NN10.19L1-NGS/2018	Y280-C1.2	Y280-C1	SH5	F98	F98	G1	F98	F98
A/chicken/Guangxi/NN10.19K2-NGS/2018	Y280-C1.2	Y280-C1	SH5	F98	F98	G1	F98	F98
A/Guangxi/NN10.19T-NGS/2018	Y280-C1.2	Y280-C1	SH5	F98	F98	G1	F98	F98

Note: Y280-like is represented by A/duck/Hong Kong/Y280/1997; SH5-like is represented by A/chicken/Shanghai/5/2003; F98-like is represented by A/chicken/Shanghai/F/1998; and G1-like is represented by A/quail/Hong Kong/G1/1997.

## Discussion

Based on similar clinical signs in two human cases and epidemiological, serological, and virological studies, a cluster of human cases, a sick pet cat, and chicken infections caused by H9N2 AIVs were identified at a backyard farm in Guangxi Province, China, in October 2018. Four H9N2 AIVs were isolated from a patient, cat, and chicken. Nucleotide sequence identity of over 99.9% for each gene segment of the four H9N2 isolates indicated that only one type of H9N2 AIV circulated in the farm and was later transmitted across species. At the beginning of this outbreak, the clinical symptoms first occurred in the majority of newly bought chickens rather than in the two originally raised chickens, suggesting that the H9N2 AIV was probably introduced into the farm by the imported chickens. Given the earliest clinical onset date reported in the chickens and later clinical syndromes displayed in the two persons and one cat, together with chickens were documented as the dominant host for H9N2 circulating in China, we inferred that the virus could be transmitted from infected chickens to cats and/or humans by directly or indirectly contact, which also conforms to the lack of evidence for H9N2 transmission between mammals [46]. In addition, the human and cat isolates shared the same nucleotide polymorphism at position 475aa in HA, and the human and chicken isolates also shared the same polymorphism at position 30aa of M1, so transmission route from chicken to cat then to human couldn’t be excluded. Given the above, all live poultry should be quarantined before introduction into farms. Moreover, the separation between the poultry farming area and the daily living area of farmers and companion animals should be enhanced especially in small-scale backyard poultry farms, to avoid potential interspecies transmission of AIVs. In addition, timely investigation of the AIV prevalence in poultry and wild birds near the affected farm could provide further comprehensive information to uncover the potential origin of the causative pathogens for clustered infections.

All HA genes of H9N2 viruses isolated in the present study possessed residue 226L, indicating a human-type receptor-binding preference and enhanced virus transmissibility in ferret models [10]. Moreover, the receptor-binding and hemagglutination assays further confirmed that the H9N2 isolates had an exclusive human-type receptor-binding capacity. Previous research has reported that the Q226L substitution in the H9N2 HA gene increased in China and that the virus possessed a dual human and avian receptor-binding capacity [8,47]. However, the nationwide influenza surveillance in 2016–2019 pointed out that nearly all H9N2 viruses isolated in China possessed 226L in the HA gene and exclusively bound to the human-type receptor [1]. As the receptor-binding capacity is key for influenza virus infecting host cells [48–51], the strong and even exclusive human receptor-binding specificity indicates a high risk and threat of H9N2 spreading from avian to human and causing more human infections. Additionally, HA T138A resulting from nucleotide polymorphisms was found in the H9N2 virus isolated from the human, and the position is located on the 130-loop of the RBD. Previous research has reported that the S138A substitution increased binding to the human-type receptor for A/H7N9 AIVs [22]. In addition, the receptor-binding site HA K137N was found in the human, cat, and chicken H9N2 isolates. Polymorphisms in the RBD should be traced to understand the human infection risk of H9N2.

Typically, human infections with H9N2 develop mild illness and influenza-like syndromes, although several severe respiratory infections and fatal cases have also been documented [4]. The two patients infected by H9N2 AIVs in the present study showed mild symptoms, such as diarrhoea, abdominal pain, and arthralgia, rather than the influenza-like syndromes reported in previous studies [4,10,11,52]. Atypical clinical syndromes would make H9N2 human cases hard to detect and diagnose. In particular, for asymptomatic infections, seropositivity against H9N2 viruses among poultry workers has been commonly reported in countries with enzootic H9N2 in poultry [4,53–55]. Moreover, silent infections of H9N2 virus in humans could increase virus adaptation in humans and the risks of emergence of the variant with stronger virulence and/or transmissibility in humans.

Major clades have been established in the phylogenetic trees of H9N2 viruses circulating in mainland China [1]. Our four H9N2 isolates belonged to the major clades in the phylogenies of each gene. The H9N2 AIVs causing human infections were not clustered together on the phylogenies, suggesting that even genetically divergent H9N2 AIVs could infect humans. In addition, the nationwide distribution of H9N2 AIVs [1,2] further increases the probability of human exposure to the virus. Furthermore, H9N2 virus is not only dominant in chickens but also in ducks, in which more rare subtypes exist and probably accelerate the emergence of novel reassortants [1], such as the novel A/H7N9, A/H10N8, A/H10N3, and A/H5N6, that threaten human health [1,2,19].

In summary, the cluster of H9N2 infections among humans, chickens, and a cat has raised attention to interspecies transmission and disease ecology of H9N2 AIVs. The endemicity in poultry, wide spatial distribution, and multiple susceptible hosts increase the likelihood of human exposure to the virus. Furthermore, the human-type receptor preference, genetically divergent viruses that infect humans, and few overt clinical symptoms highlight the risk of infection and adaption of H9N2 in humans. Targeted prevention and control should be urgently performed to eliminate H9N2 AIVs (especially the dominant clades) circulating in poultry farms and live poultry markets. Surveillance of human H9N2 infections, especially in workers occupationally exposed to poultry, should be enhanced to monitor the emergence of human-adapted H9N2 viruses and provide early warning of a potential outbreak or pandemic.

## Supplementary Material

Supplemental Material
