# Supplementary material for: Genetic, biological and epidemiological study on a cluster of H9N2 avian influenza virus infections among chickens, a pet cat, and humans at a backyard farm in Guangxi, China
Source: Emerg Microbes Infect. 2022 Dec 18;12(1):2143282. doi: 10.1080/22221751.2022.2143282 (PMC9769140; doi:10.1080/22221751.2022.2143282)
Supplement: Supplemental Material [file Supplemental_FigS1.pdf › Supplemental_FigS1.pdf]

(A) HA

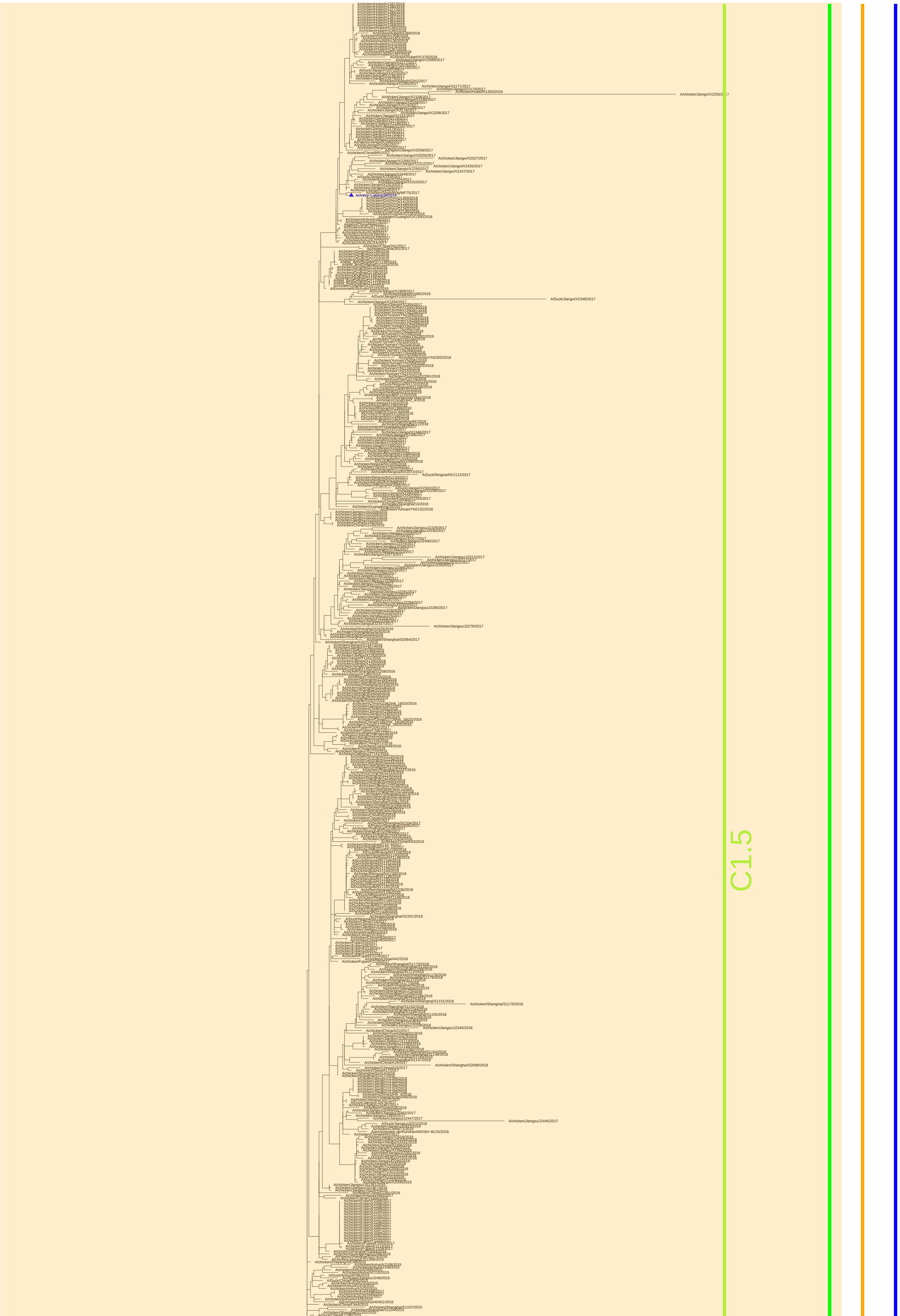

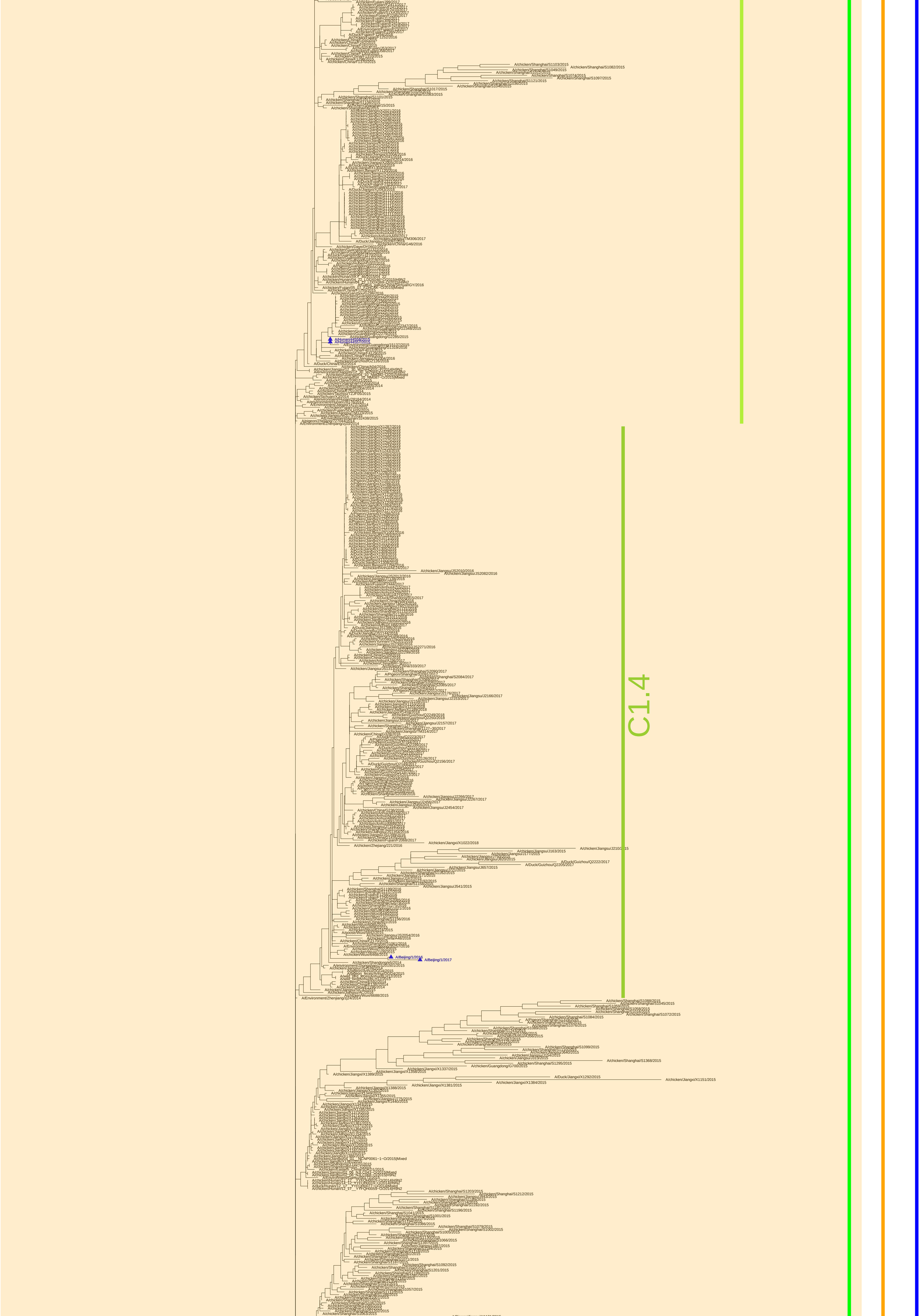
C1.4

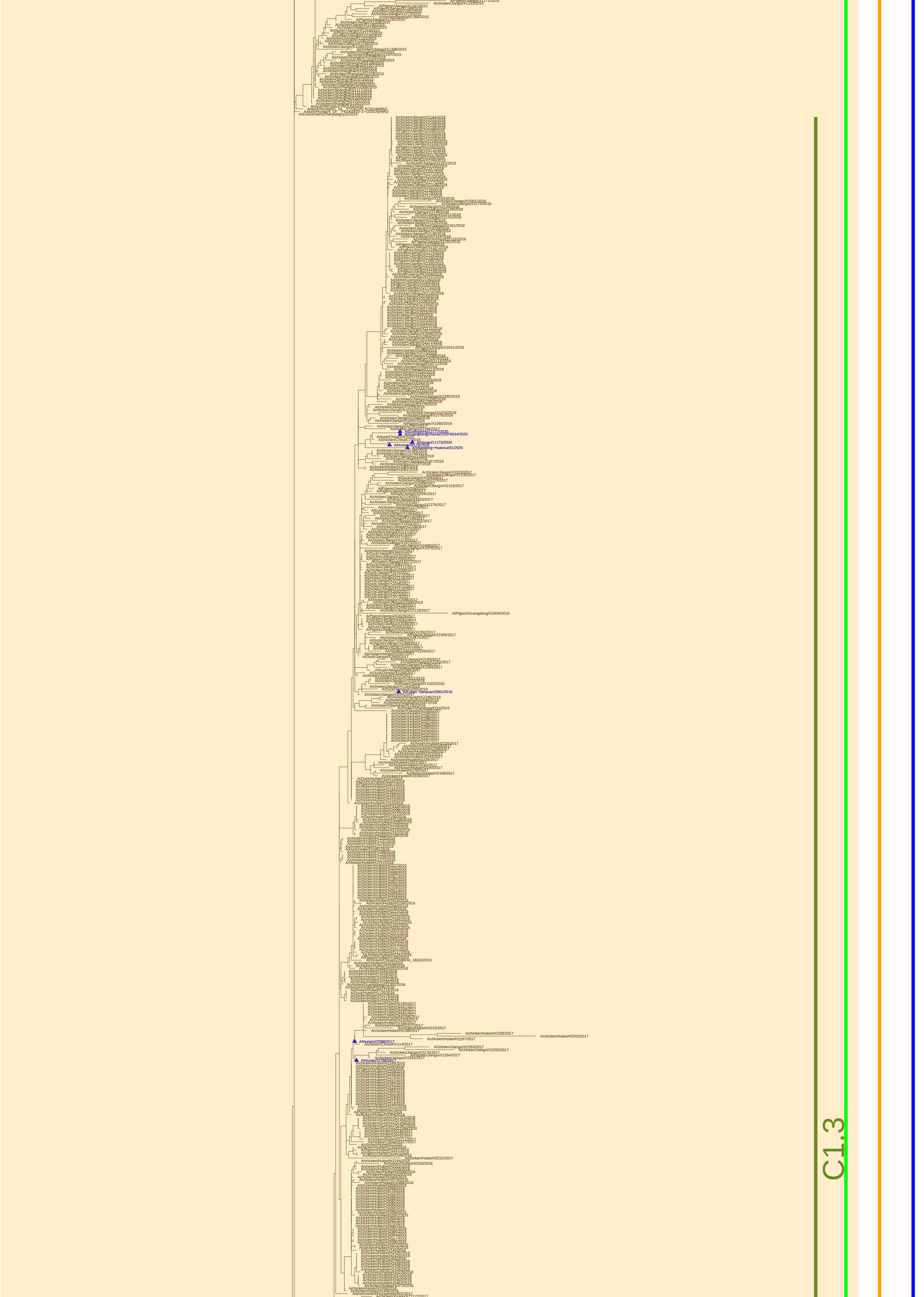
C1.3

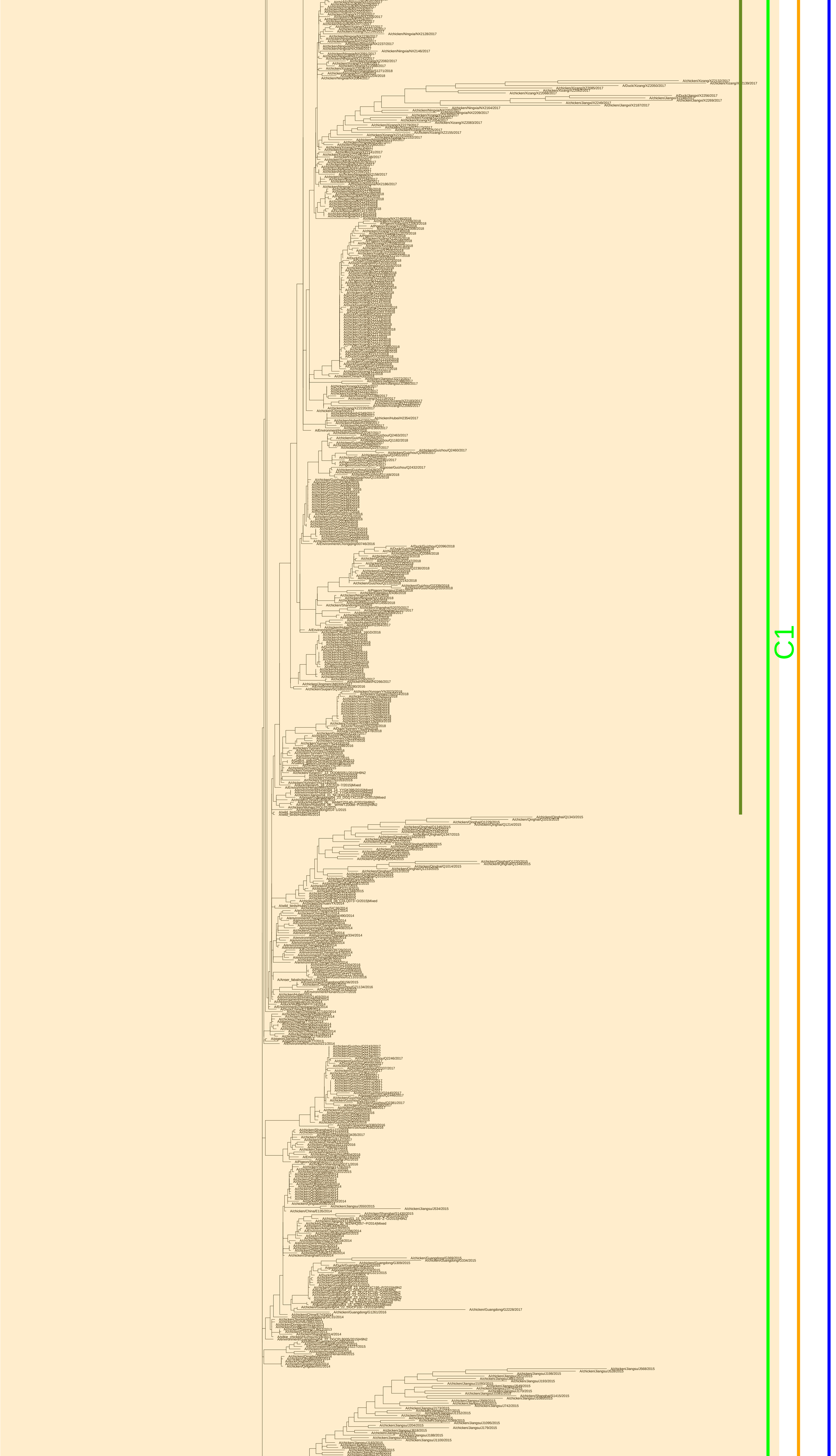
C1

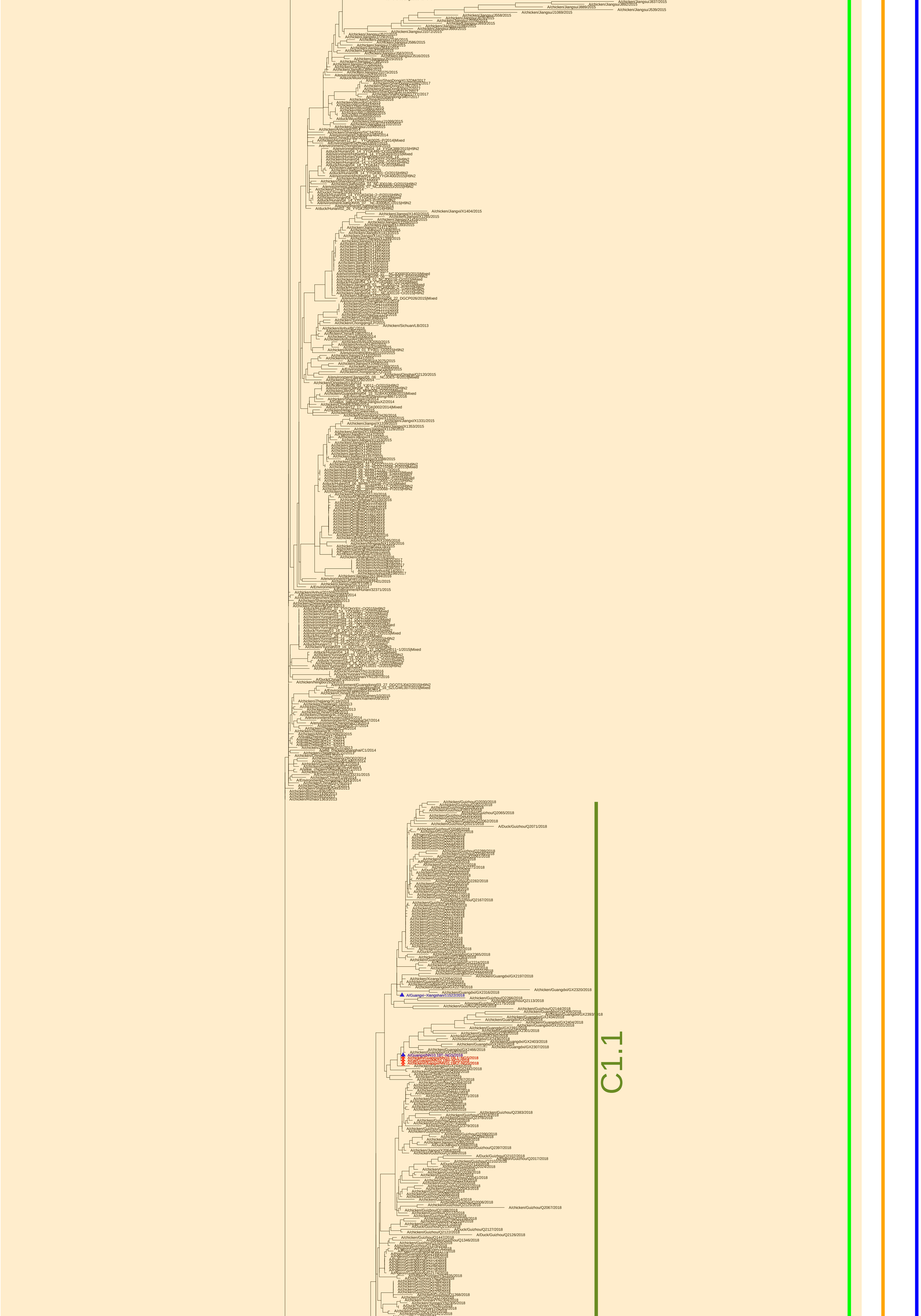
C1.1

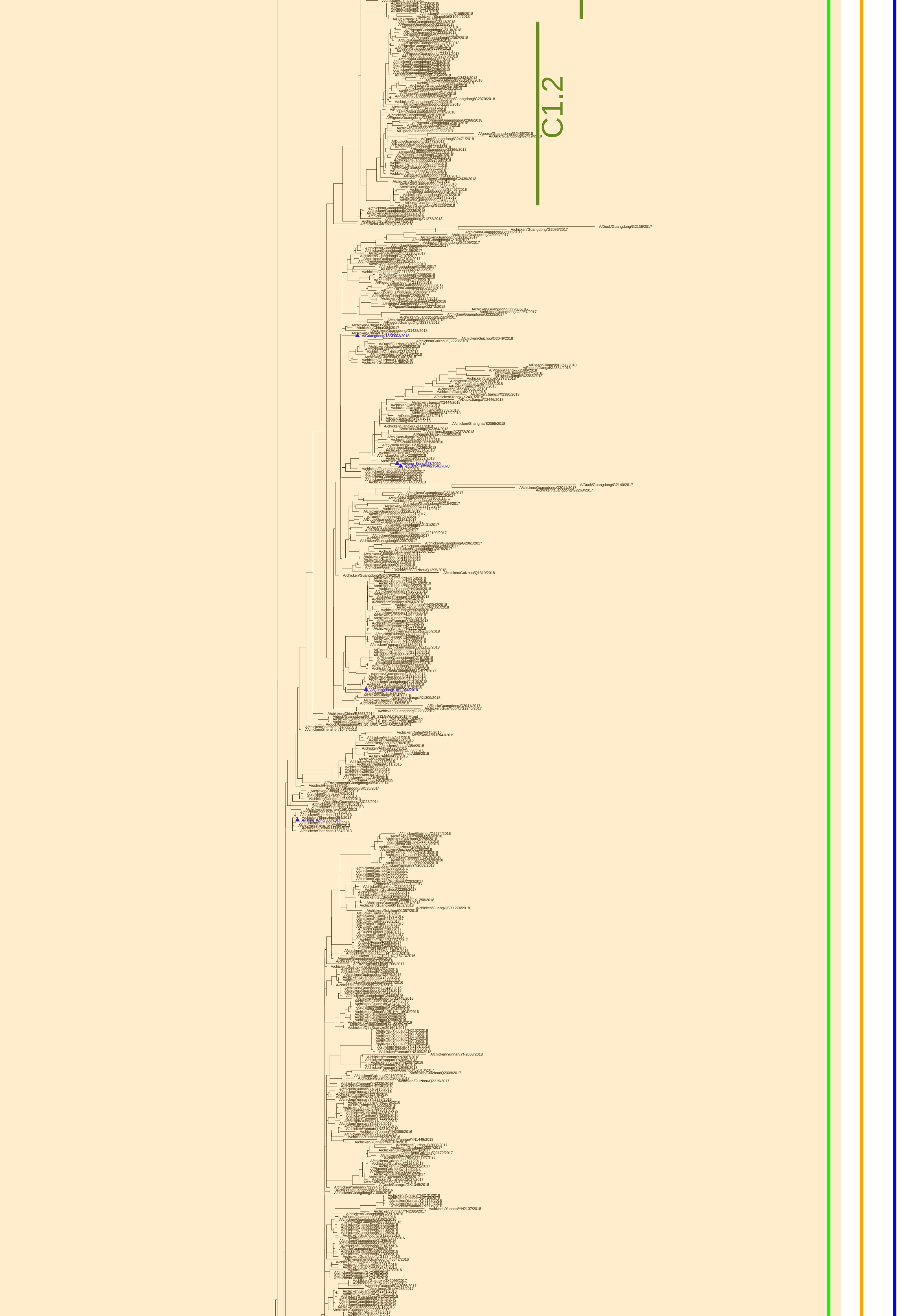
C1.2

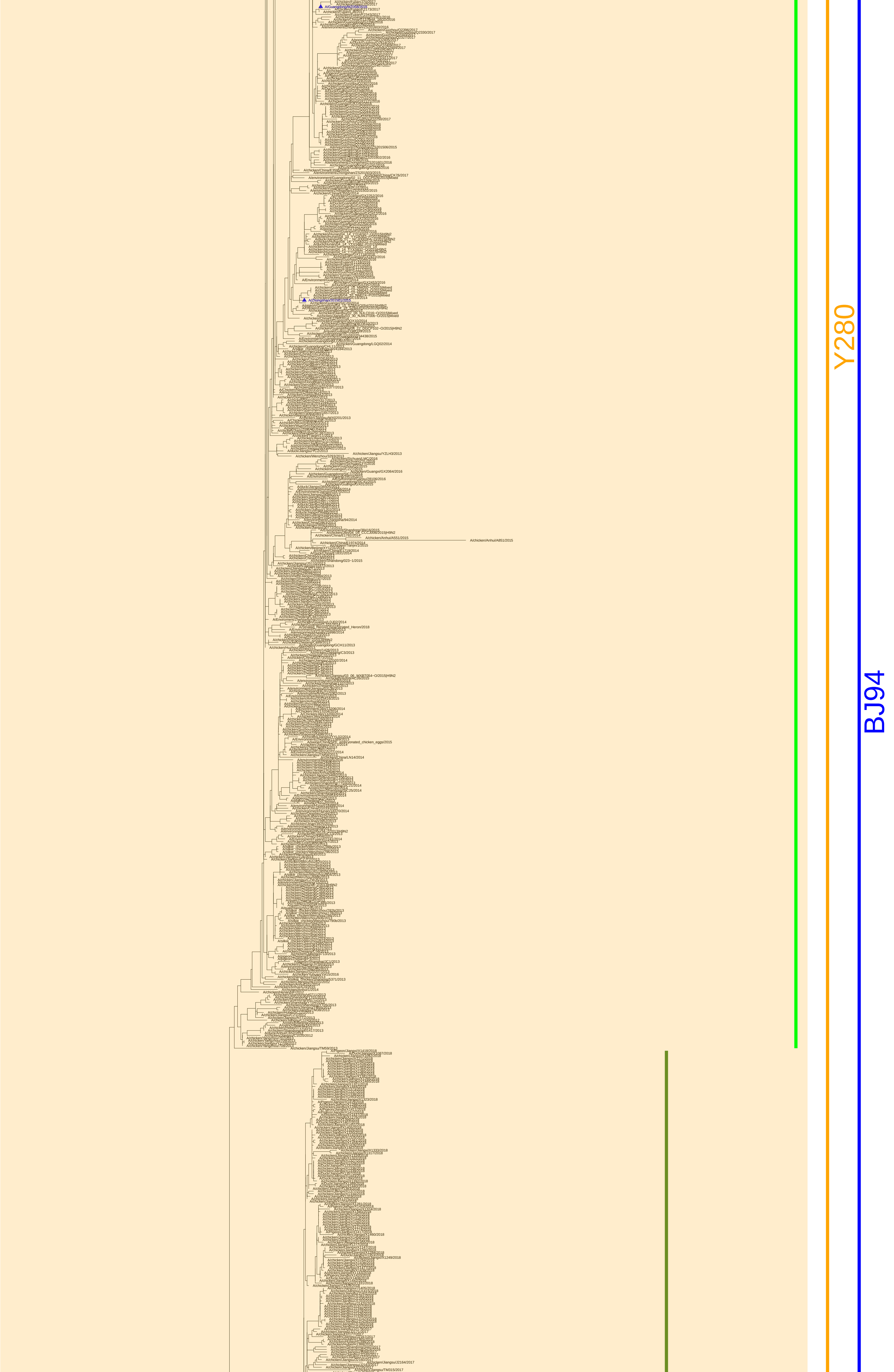
Y280
BJ94

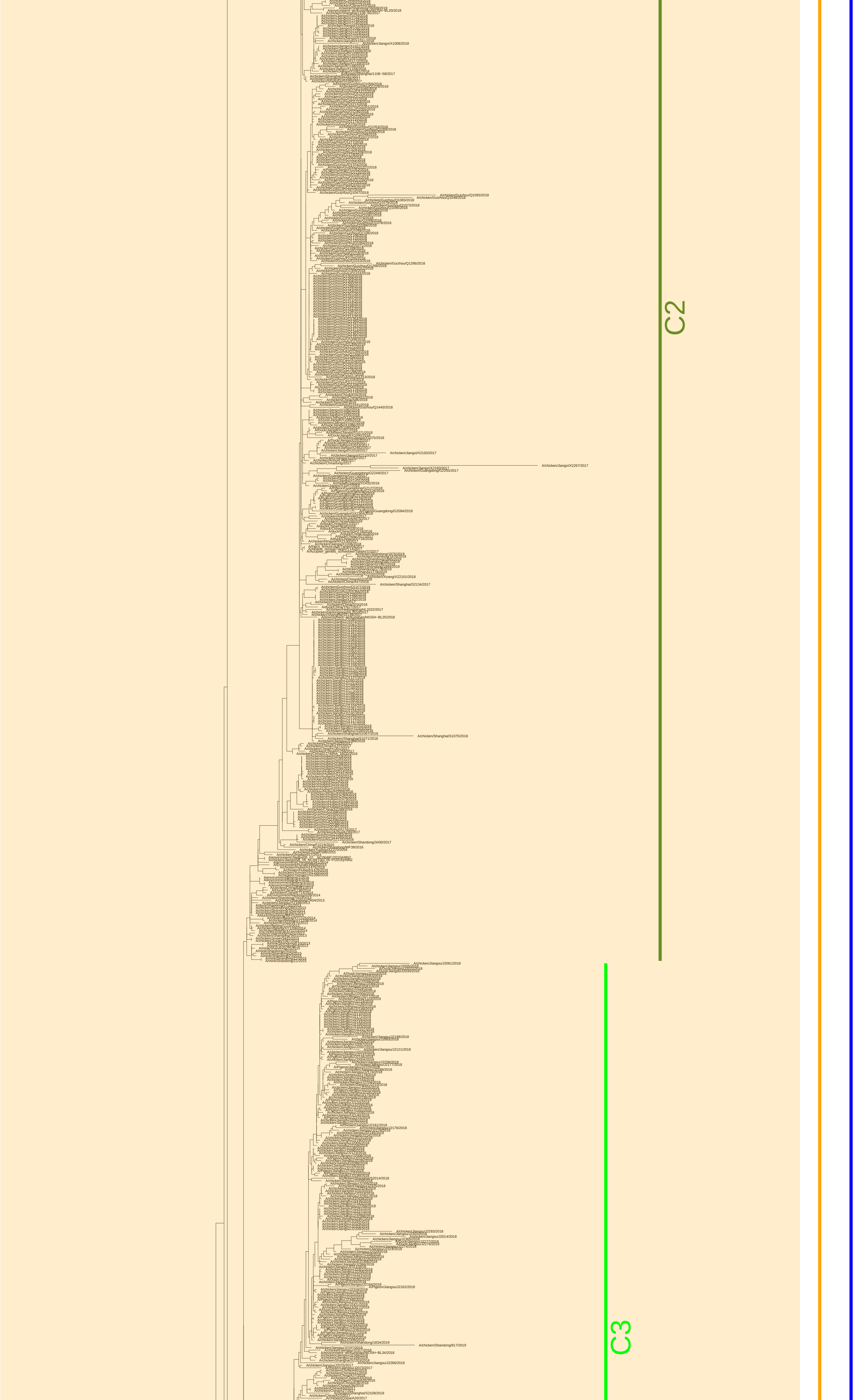
C2
C3

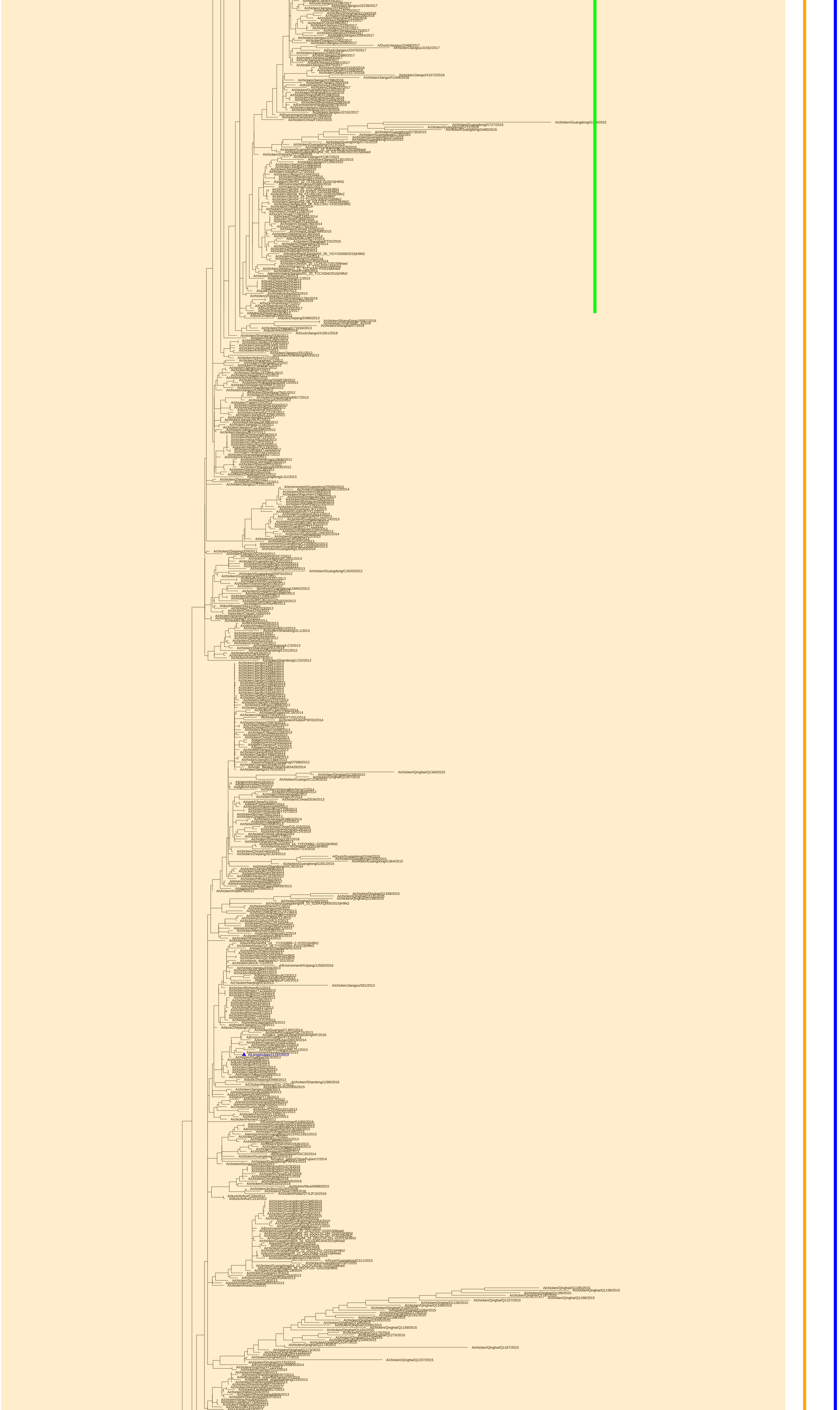
A/chicken/Guangdong/CJD02/2012
A/chicken/Qinghai/Q1340/2015
A/Environment/Xinjiang/12550/2016
A/chicken/Jiangsu/S01/2013
A/chicken/Shandong/1286/2016
A/chicken/Qinghai/Q1167/2015
A/chicken/Qinghai/Q1237/2015

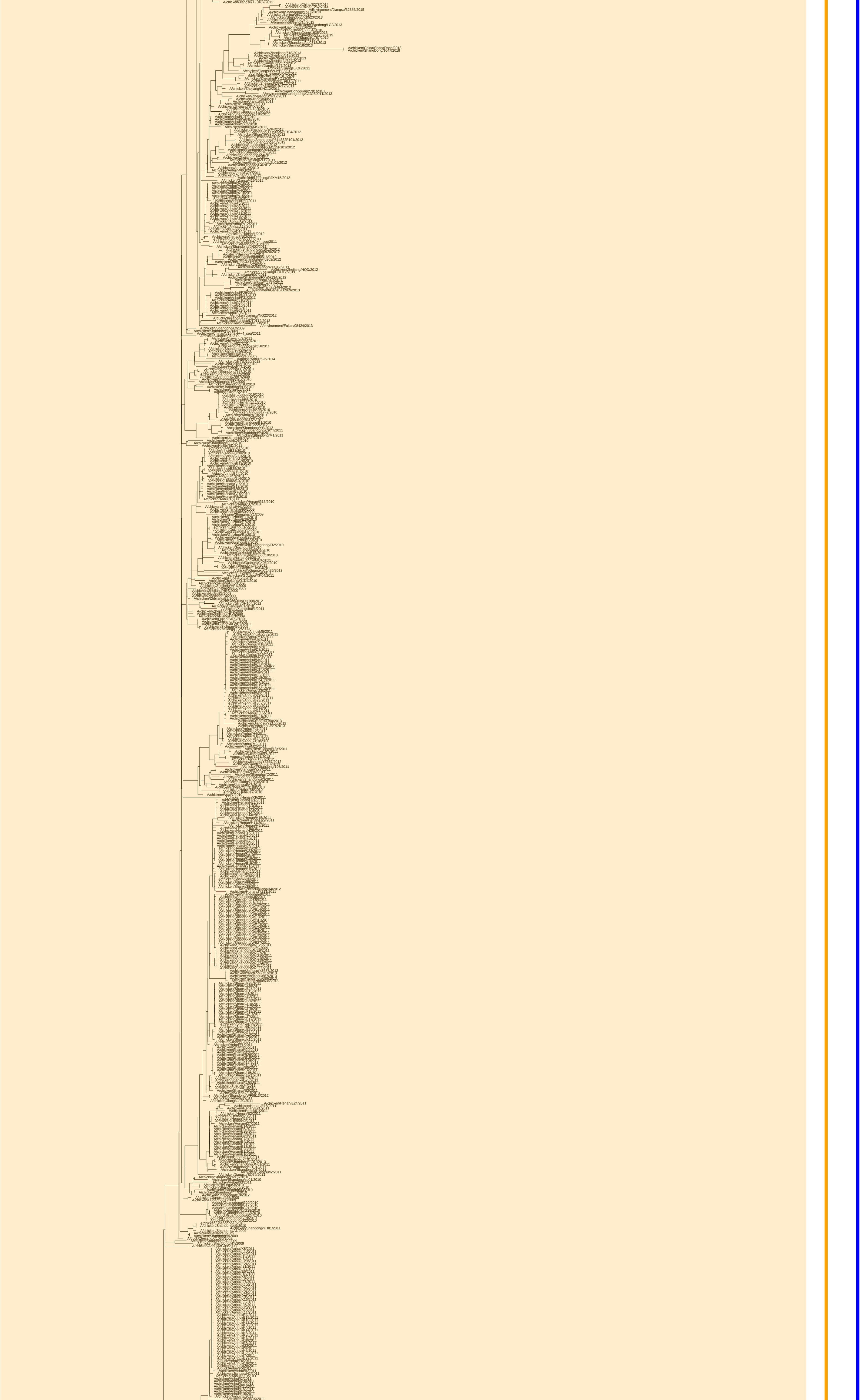

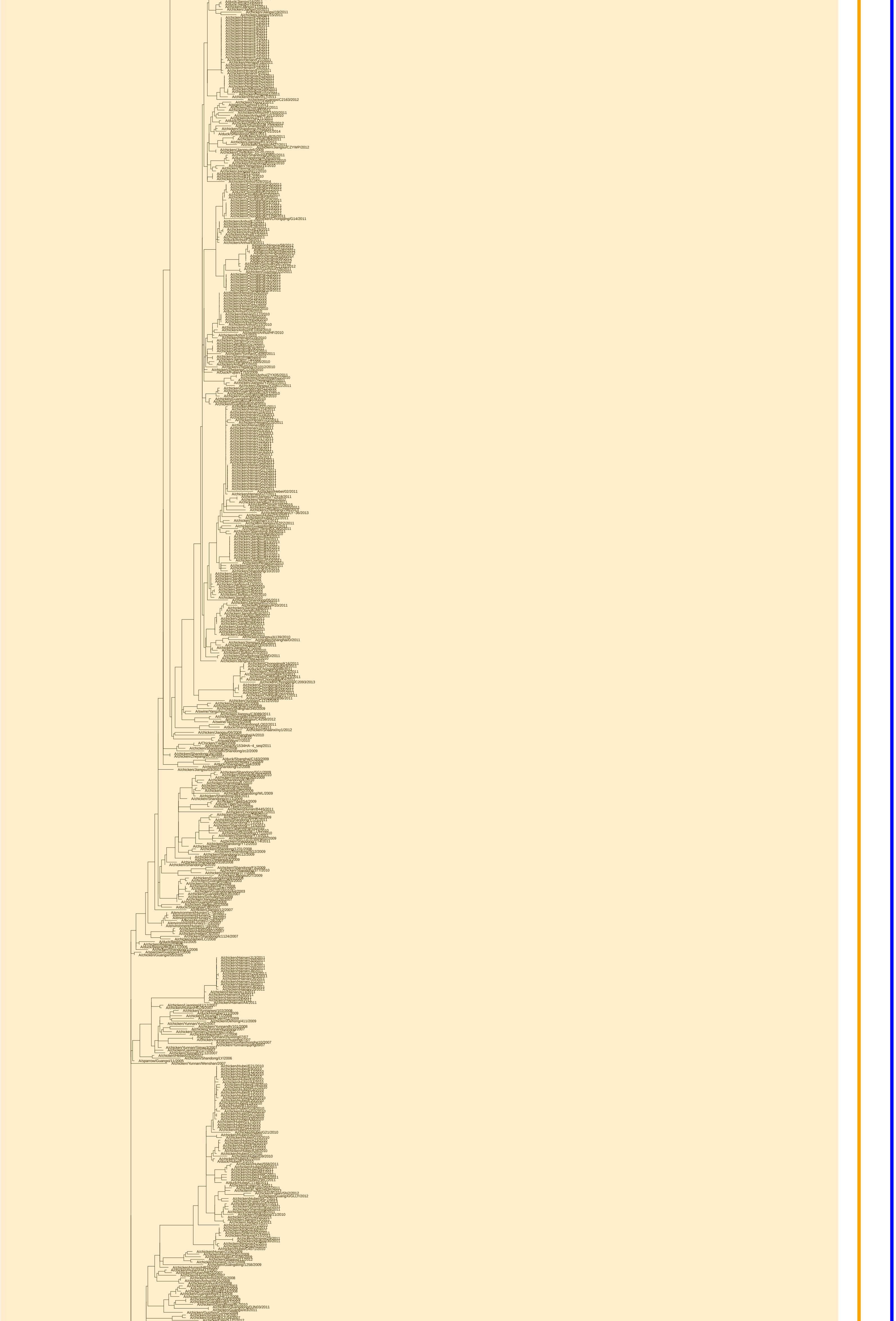

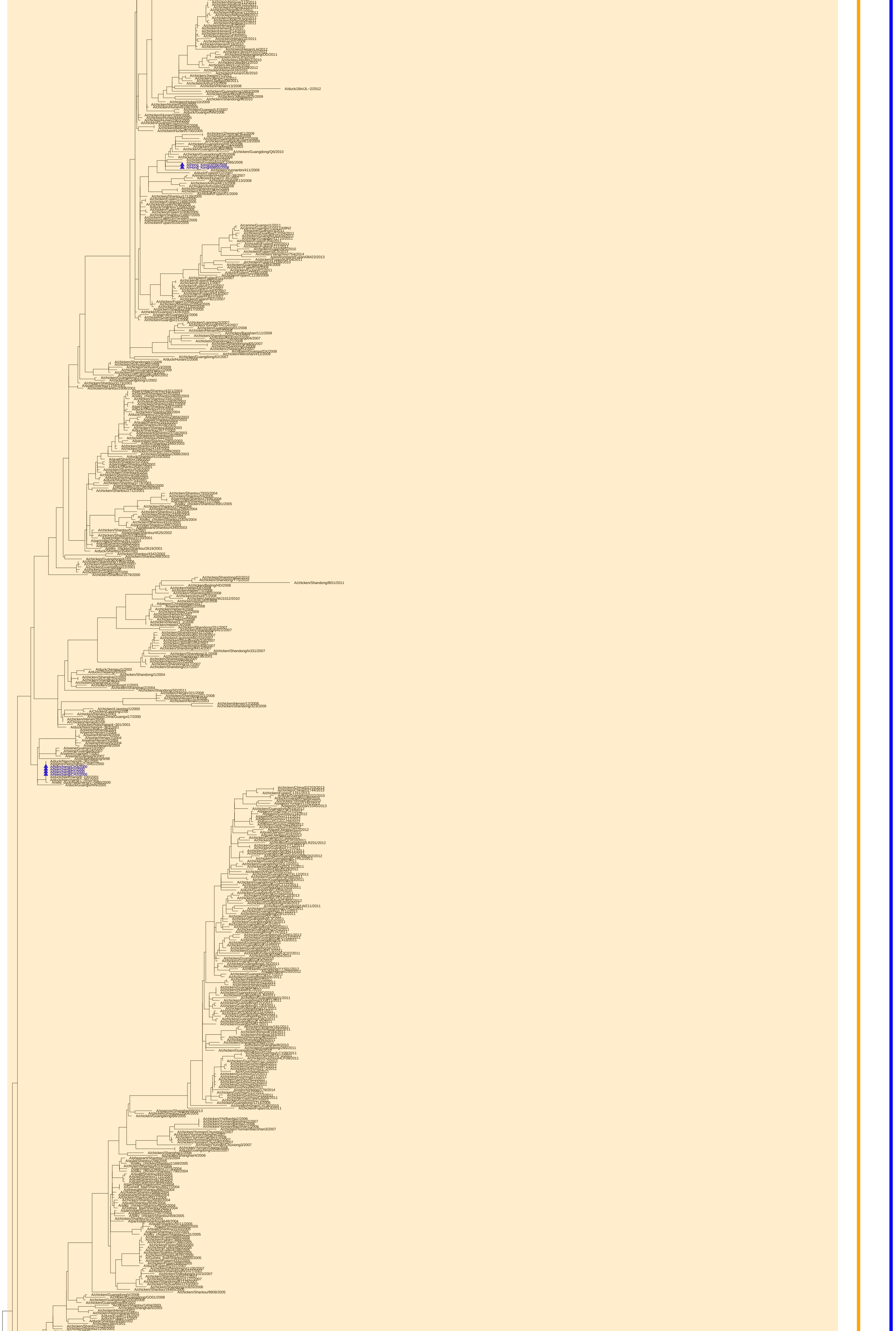
A/duck/Jilin/JL-2/2012
A/chicken/Guangdong/Q6/2010
A/chicken/Shandong/BD1/2011

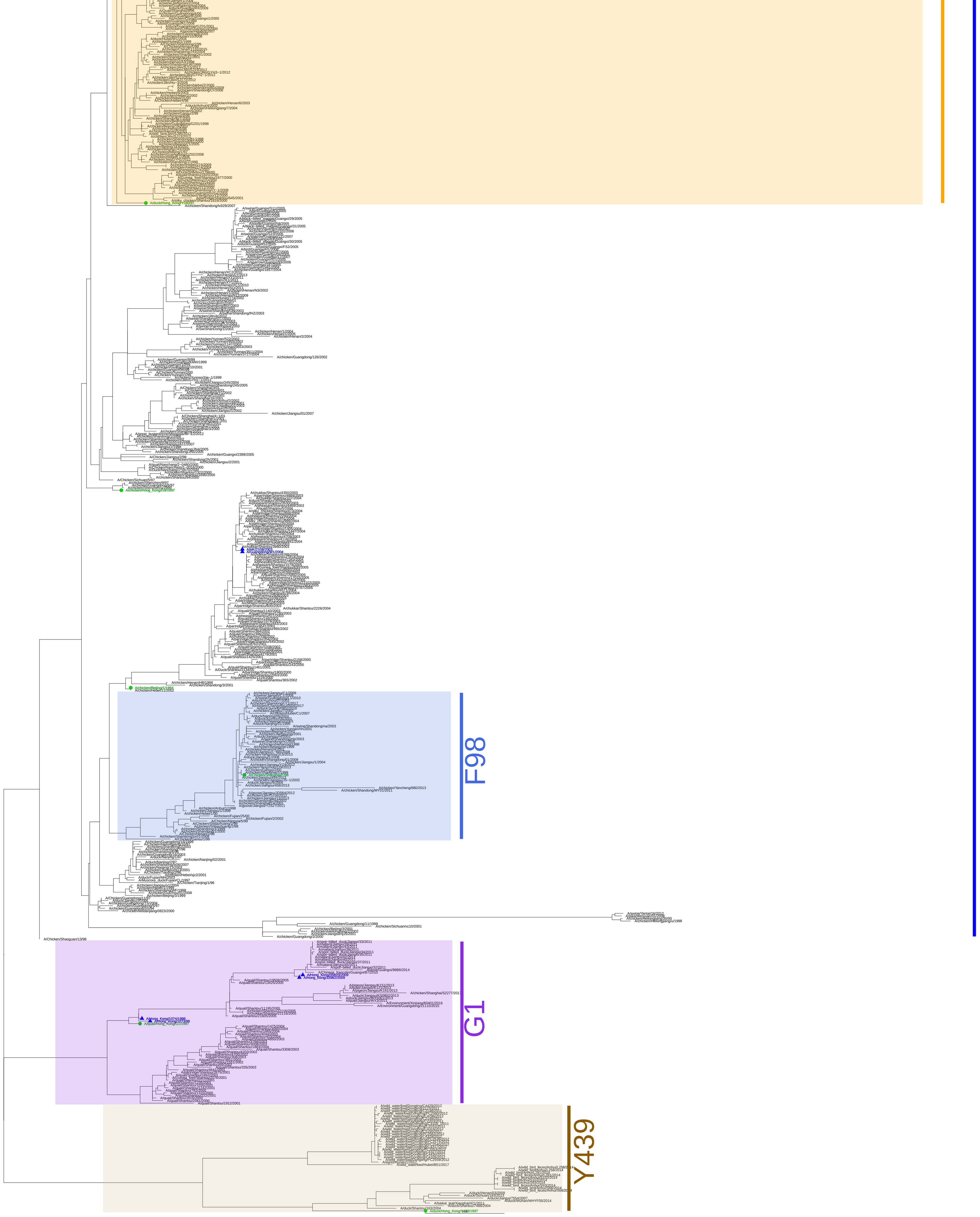
F98
G1
Y439

(B) NA

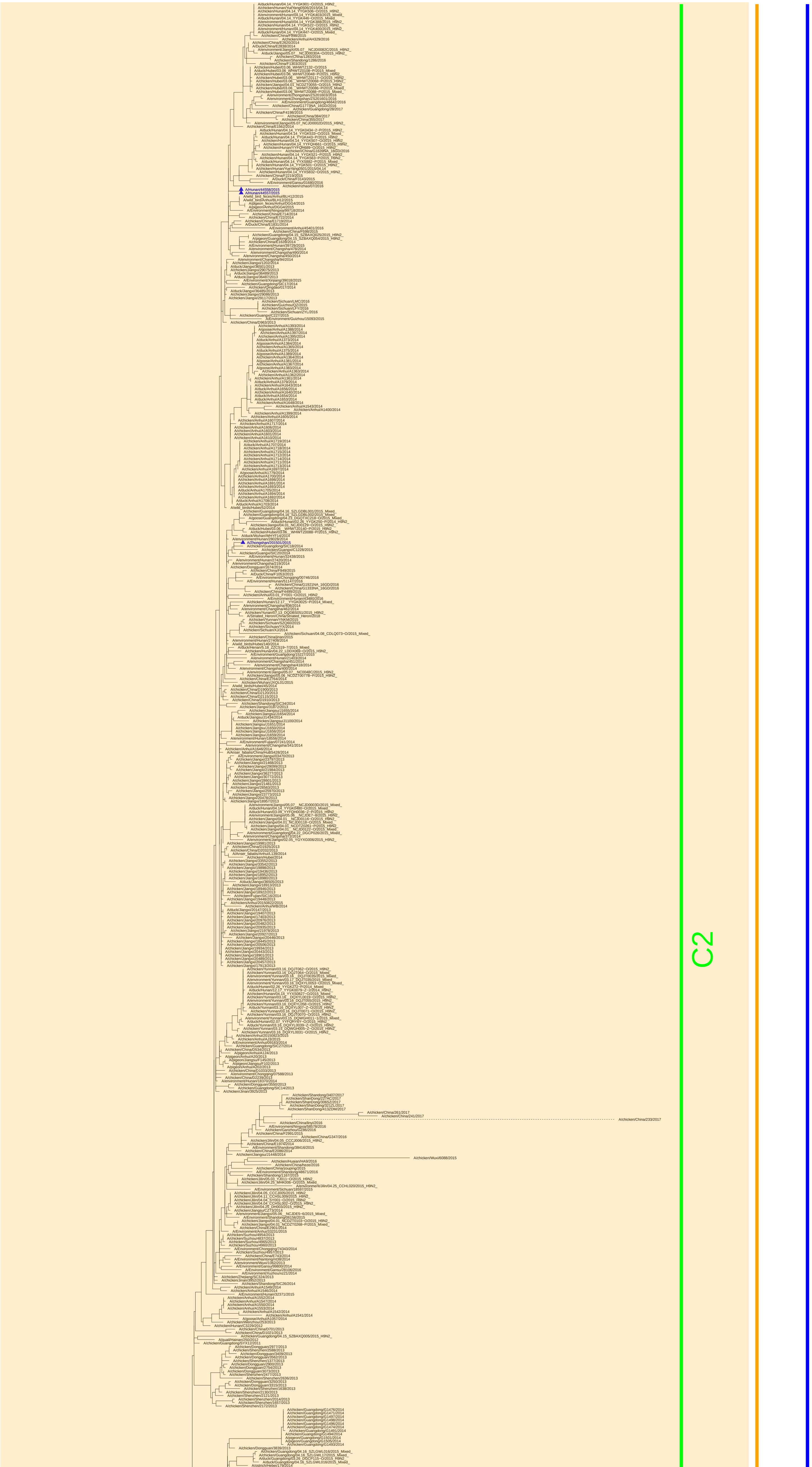

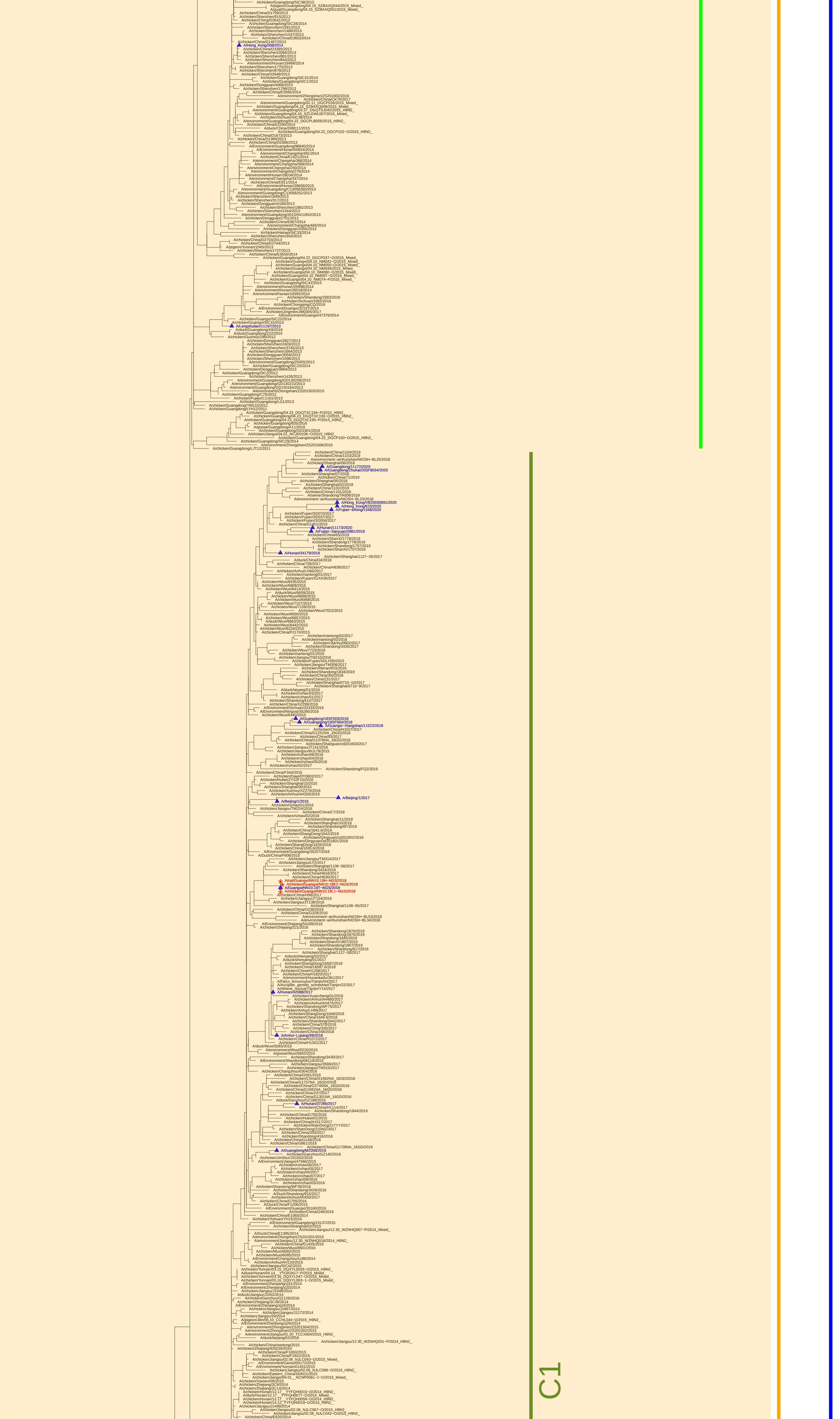
C1

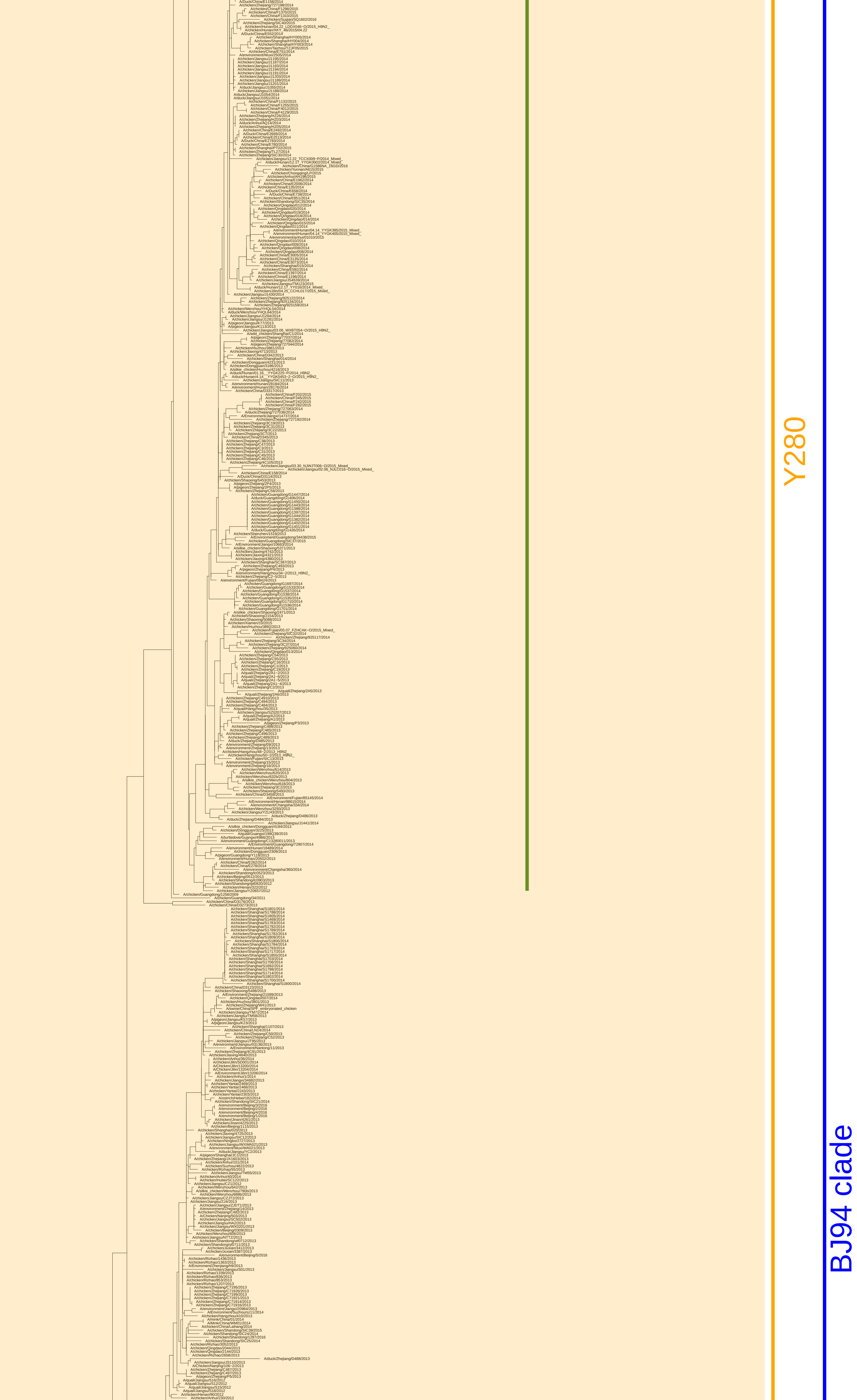
Y280
BJ94 clade

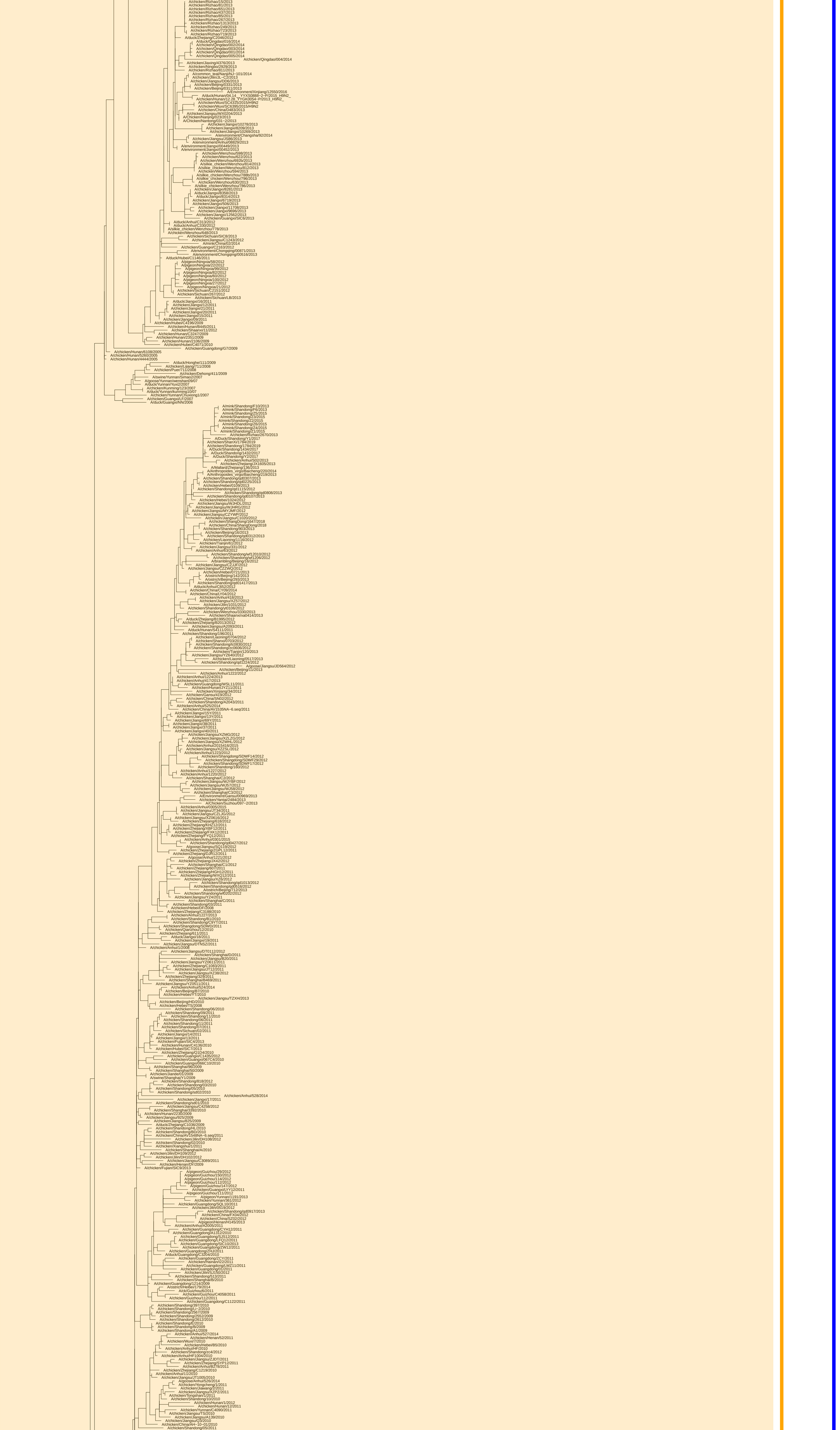

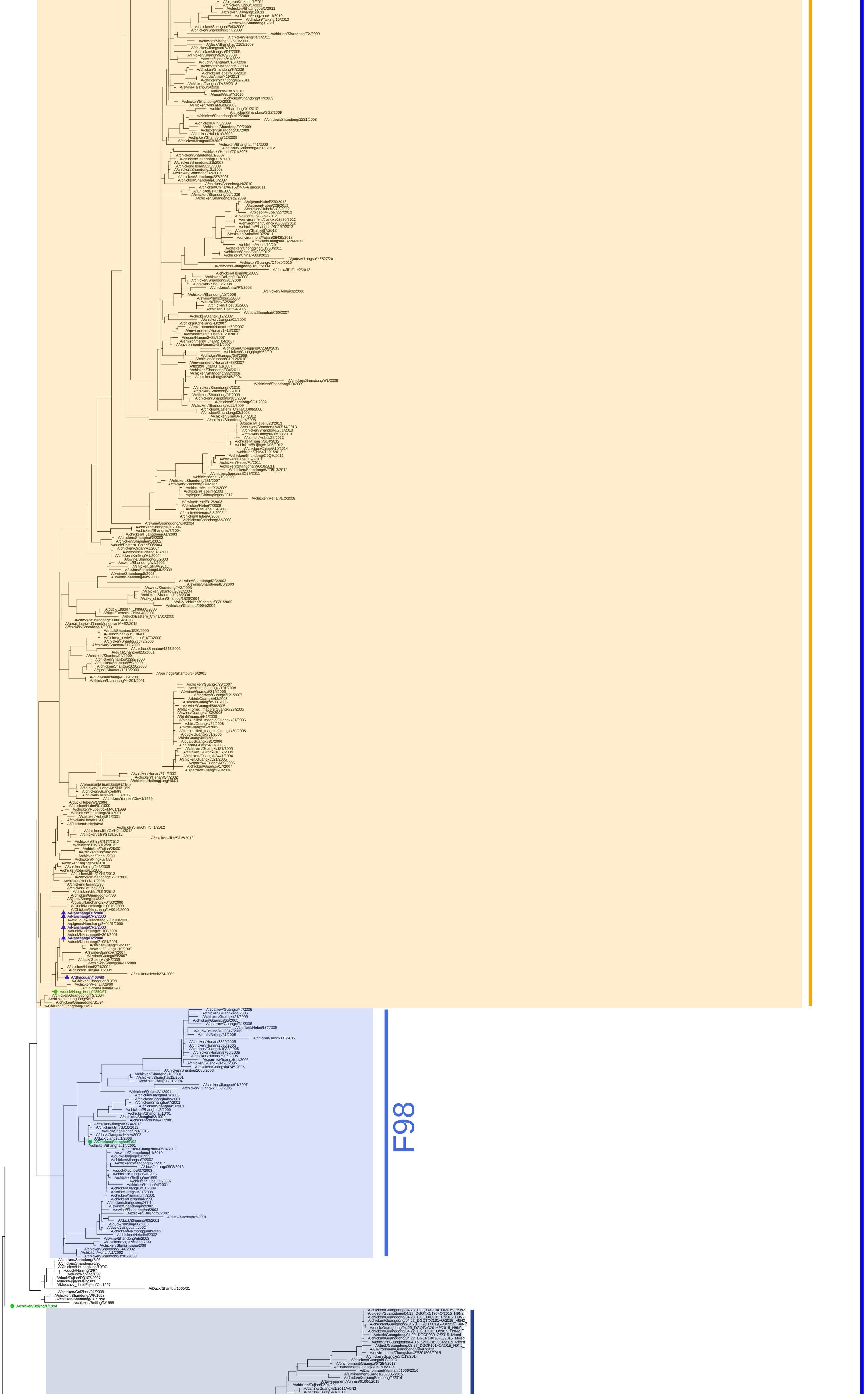
F98

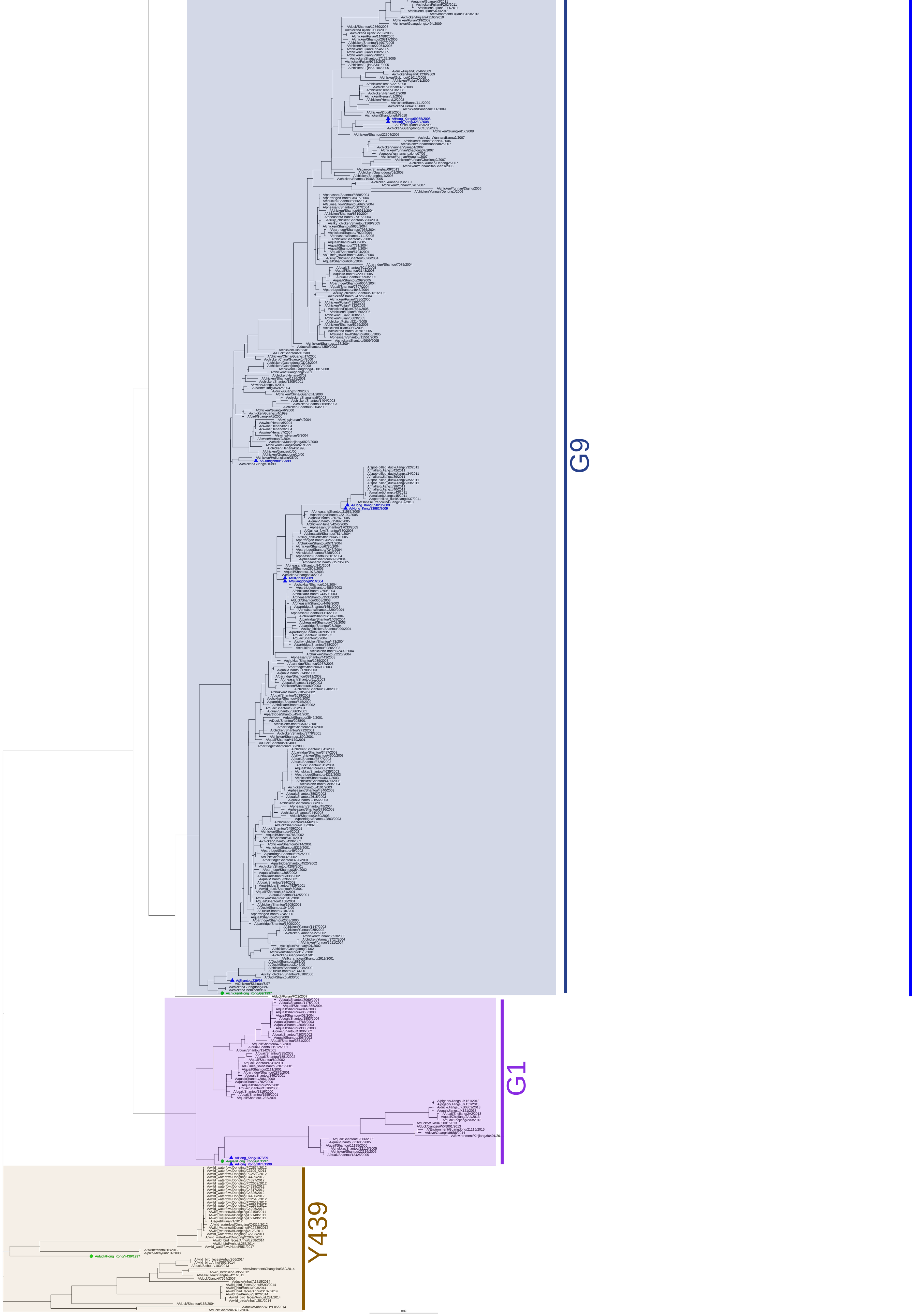
G9
G1
Y439

(C) PB2

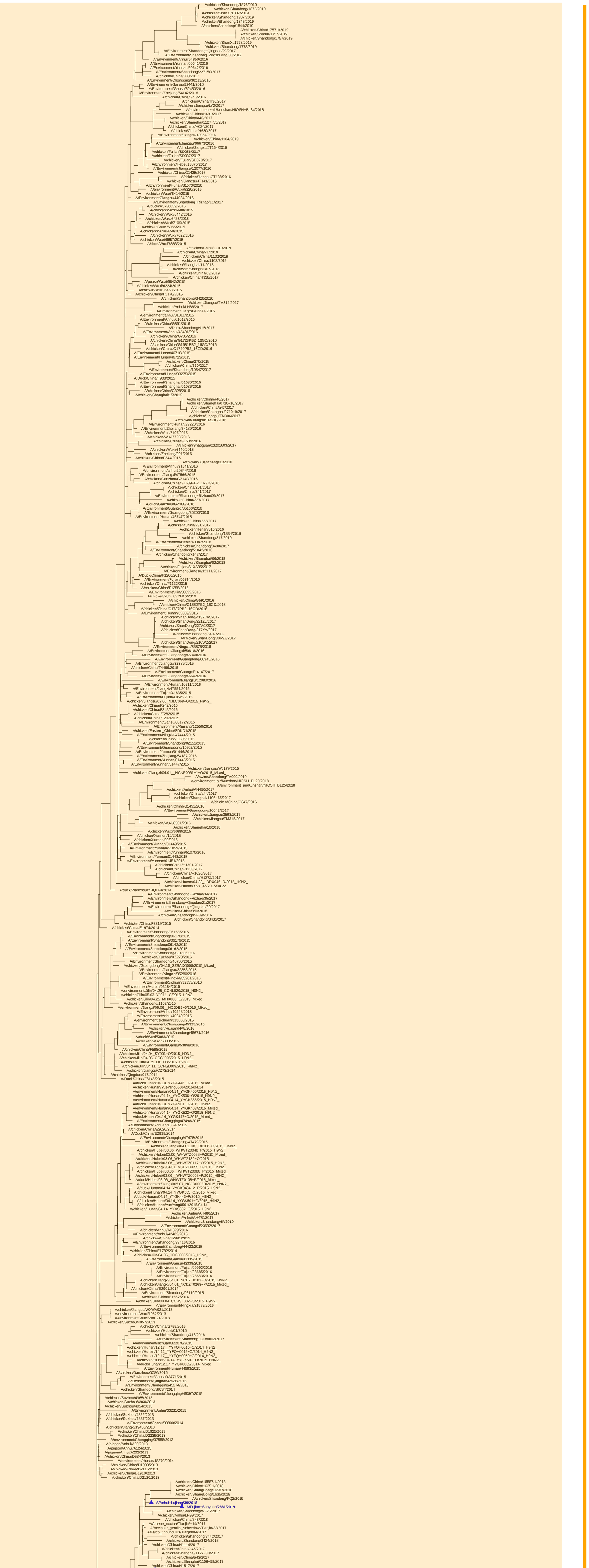

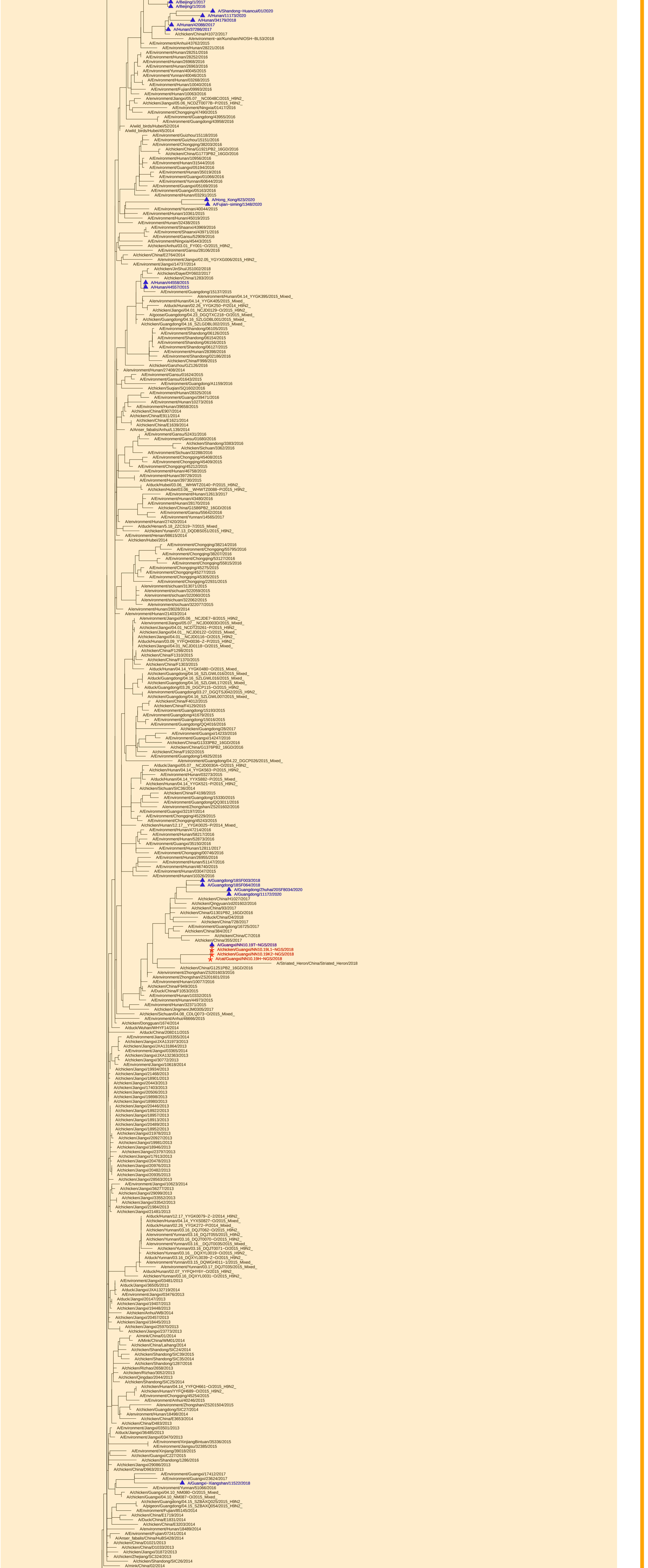

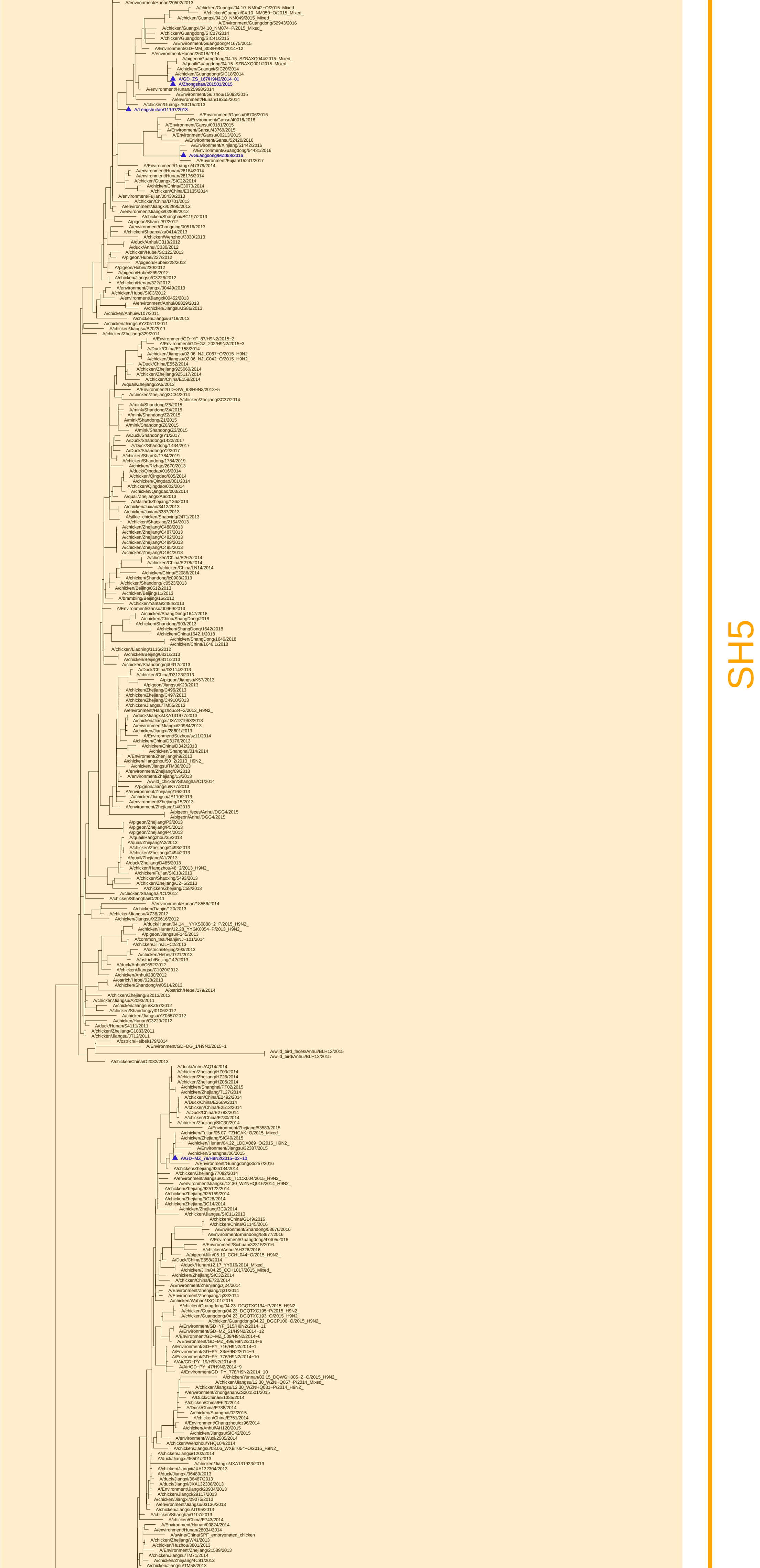
SH5

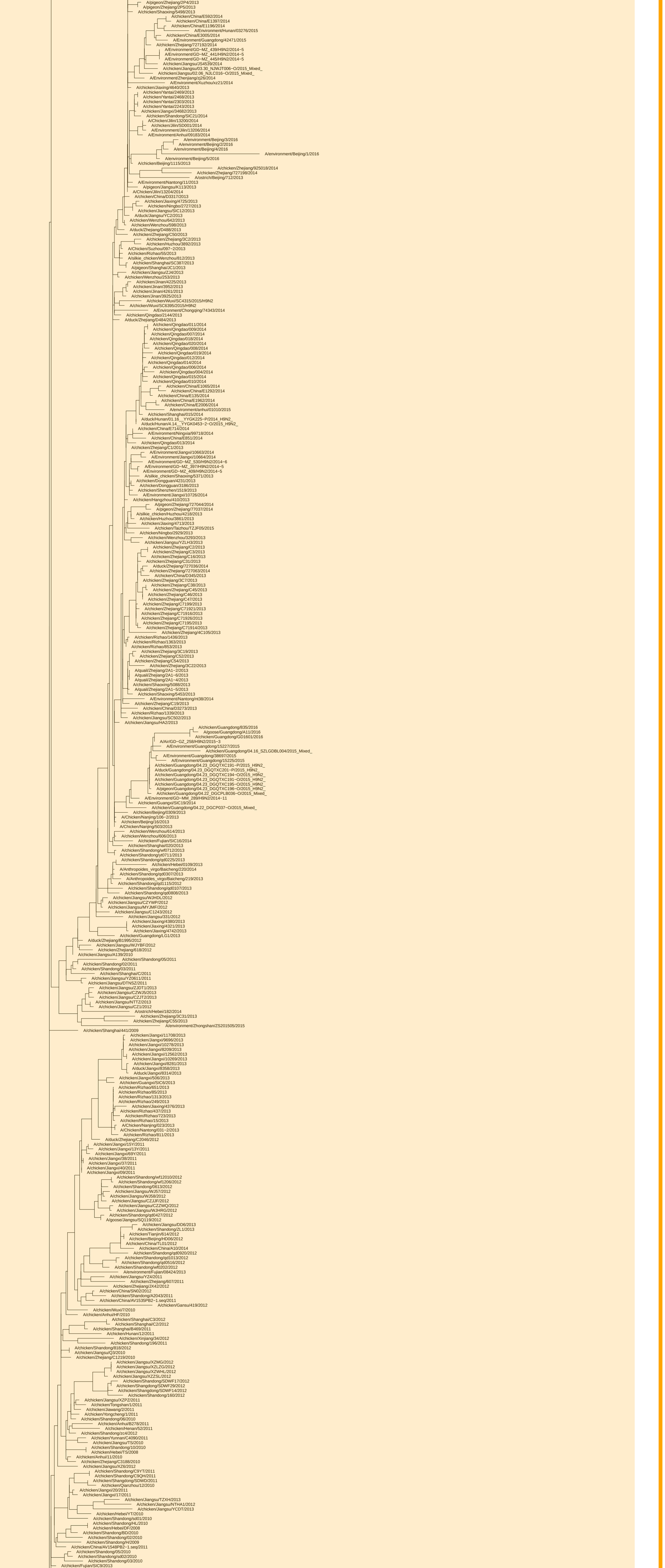

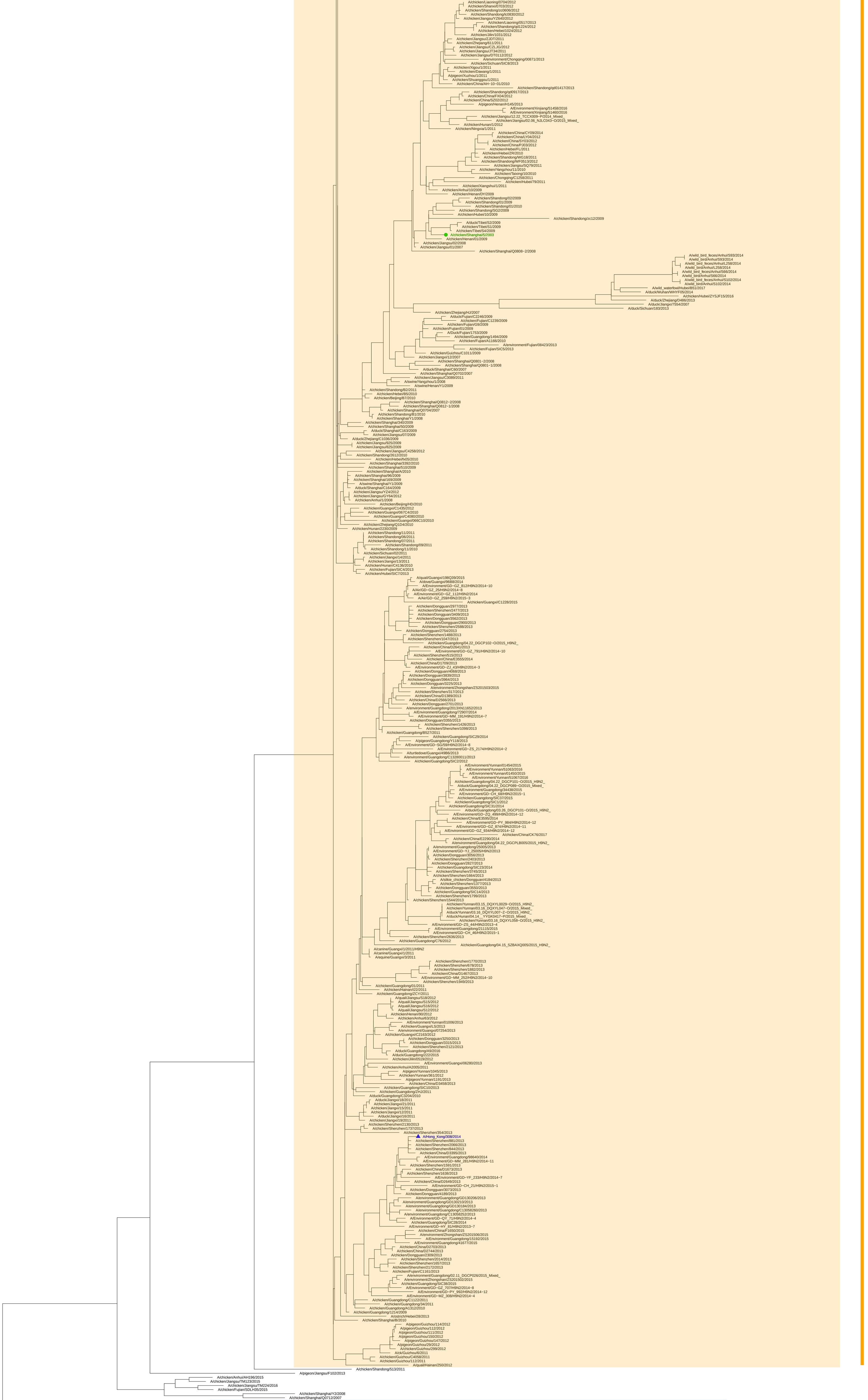

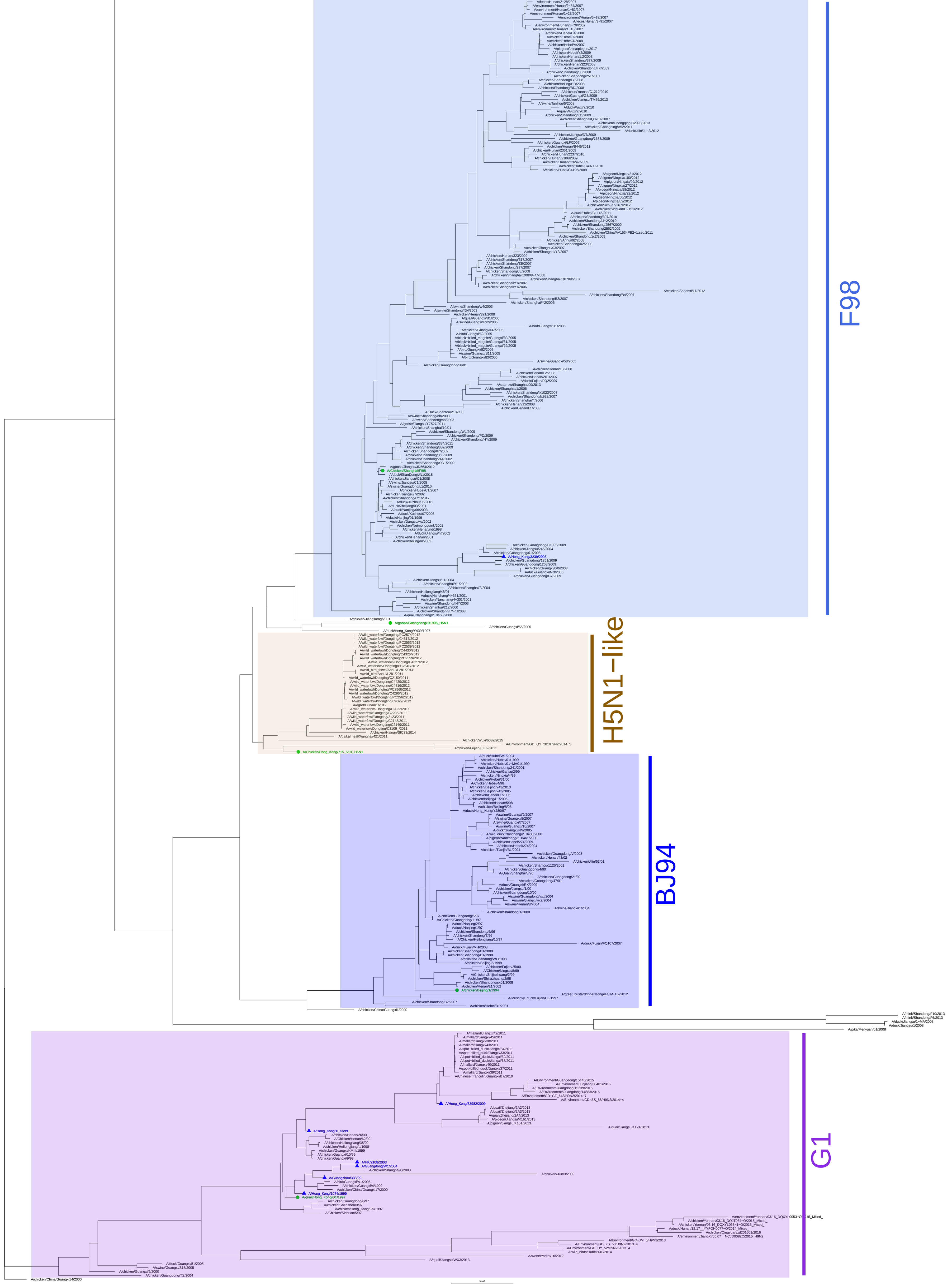
F98
H5N1−like
BJ94
G1
0.02

(D) PB1

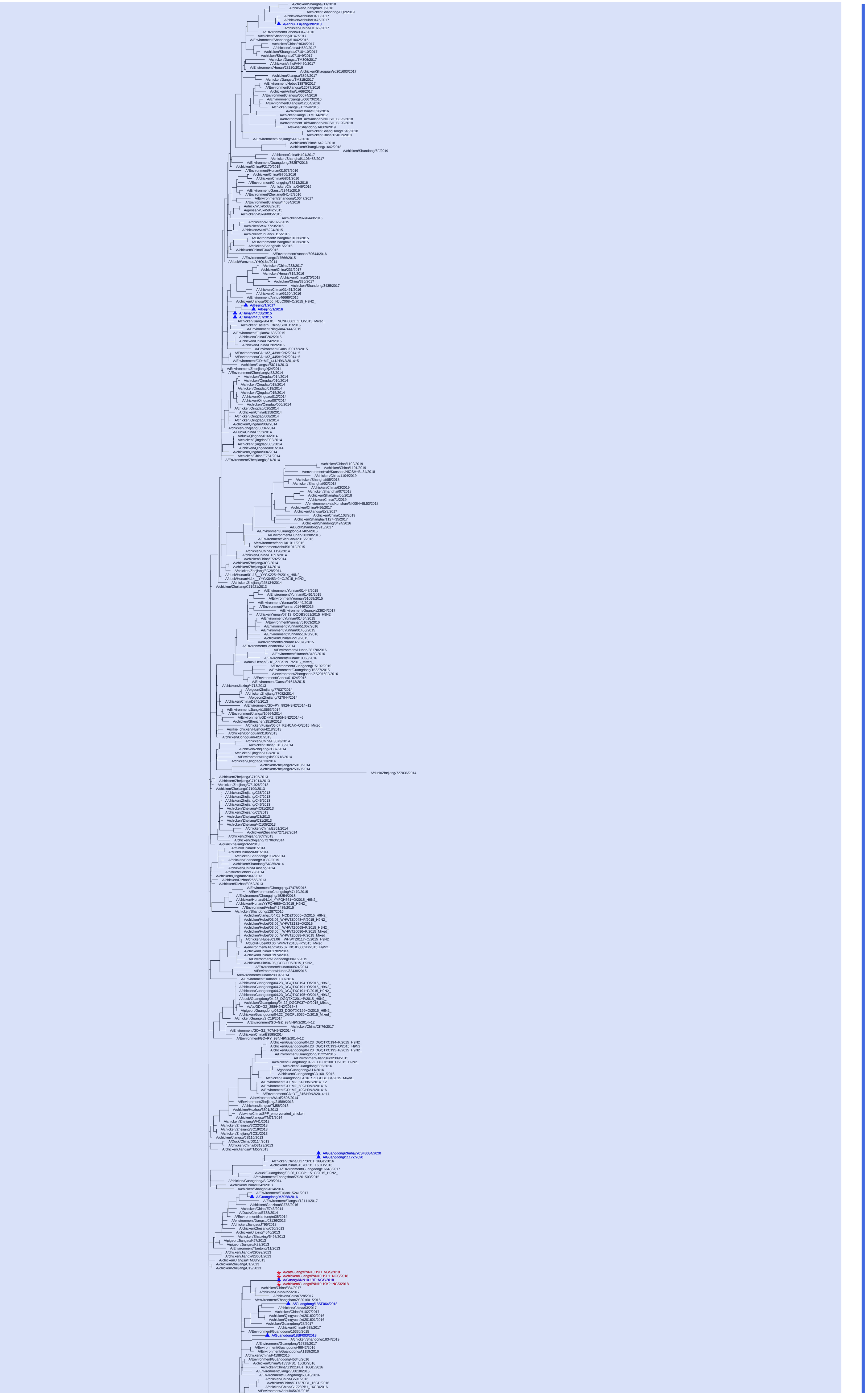

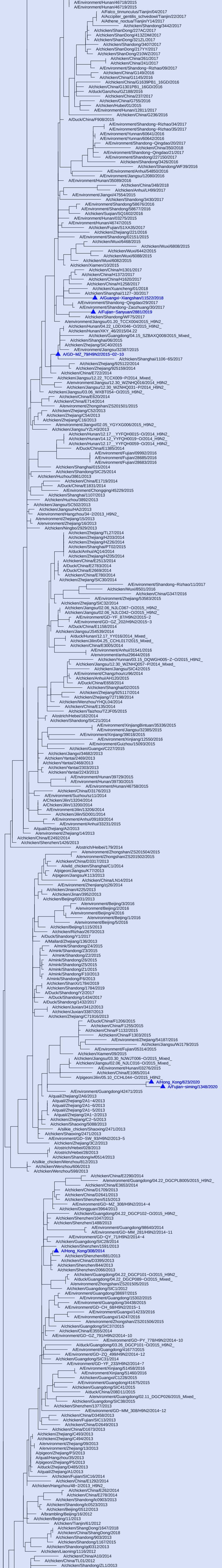
A/Guangxi-Xiangshan/11522/2018
A/Fujian-Sanyuan/2881/2019
A/GD-MZ_79/H9N2/2015-02-10
A/Hong_Kong/623/2020
A/Fujian-siming/1348/2020
A/Hong_Kong/308/2014

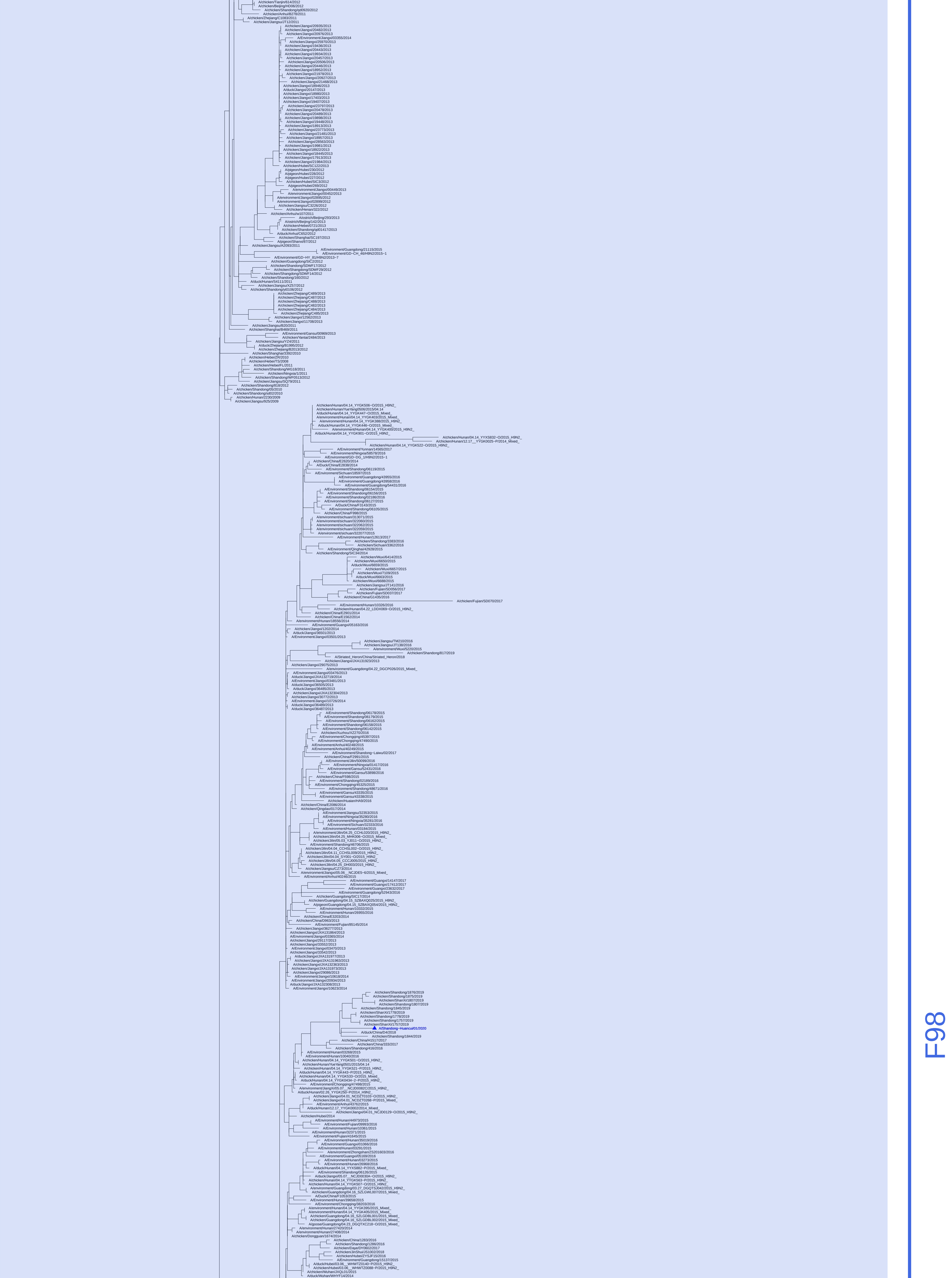
A/Shandong-Huancui/01/2020
F98

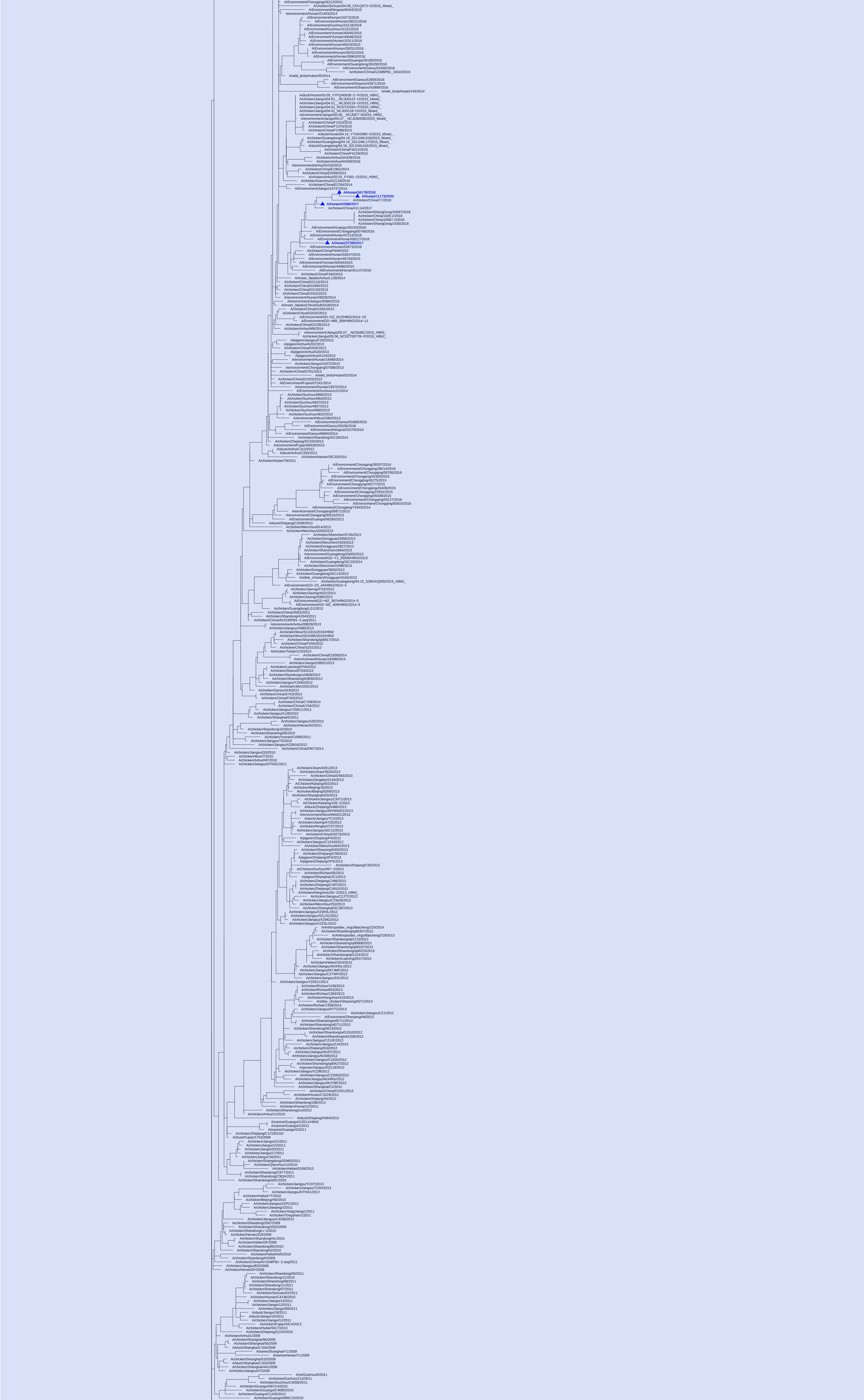

A/Hunan/34179/2018
A/Hunan/11173/2020
A/Hunan/42088/2017
A/Hunan/37286/2017

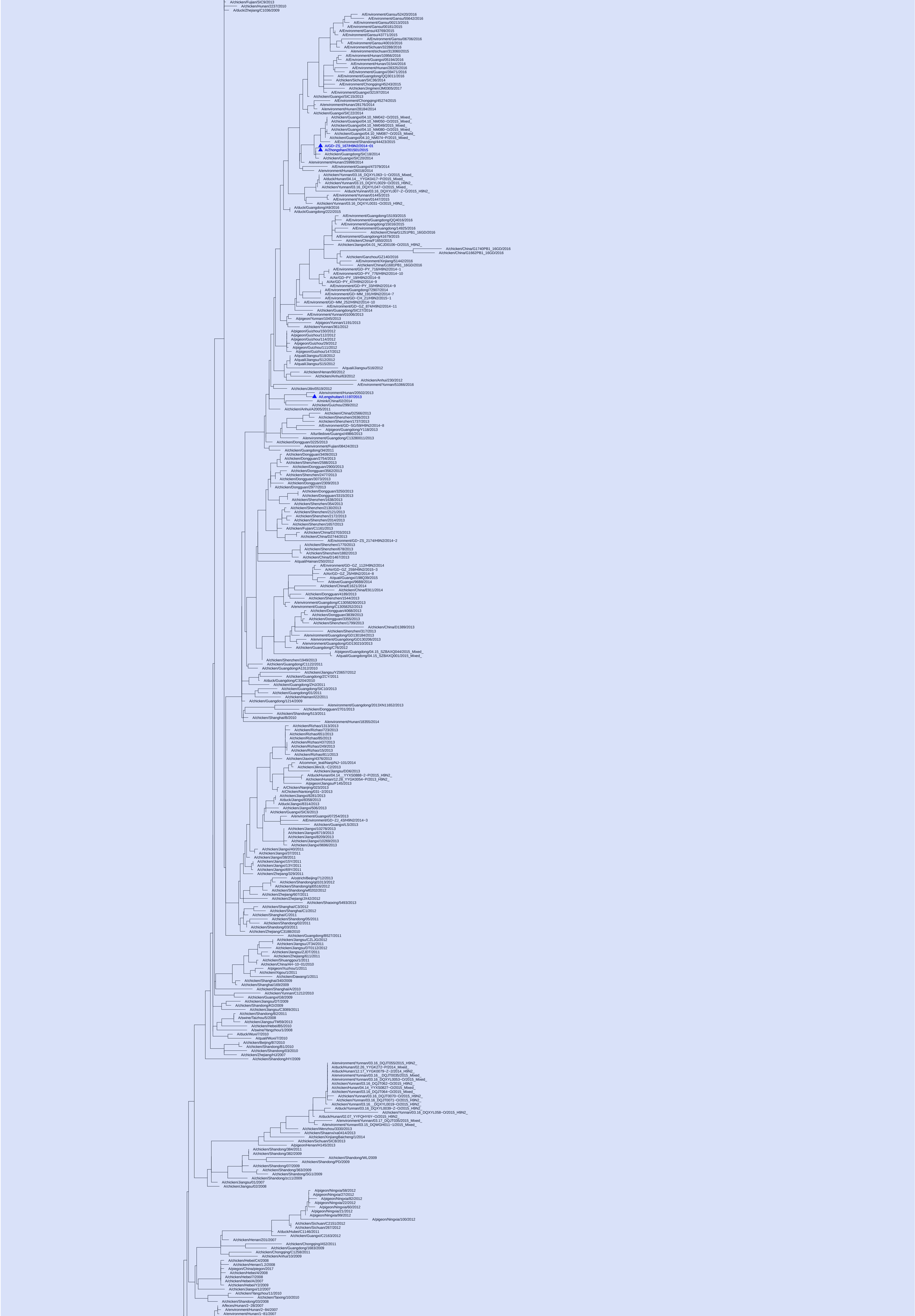

A/GD-ZS_167/H9N2/2014-01
A/Zhongshan/201501/2015
A/Lengshuitan/11197/2013

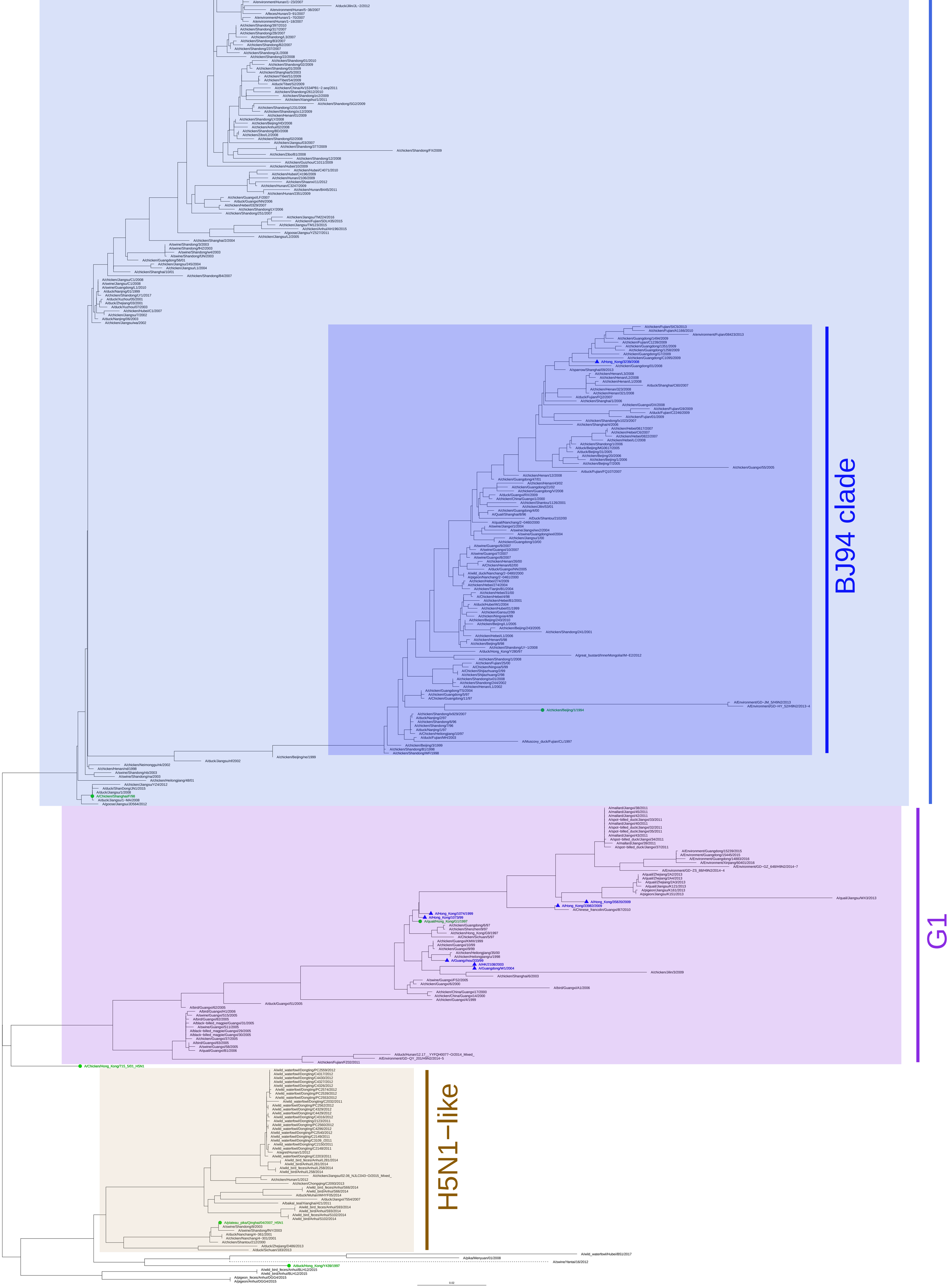
BJ94 clade
G1
H5N1−like
0.02

(E) PA

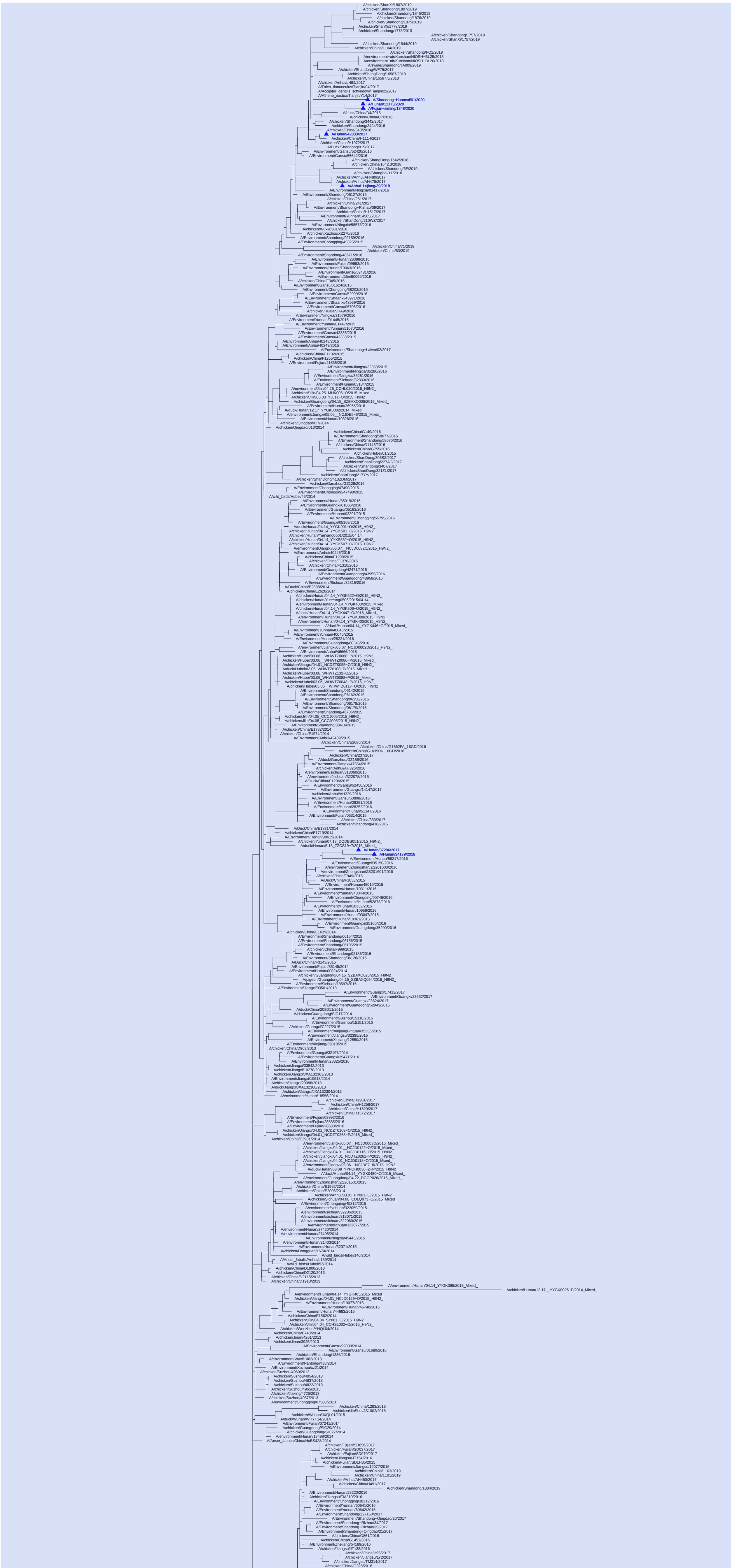

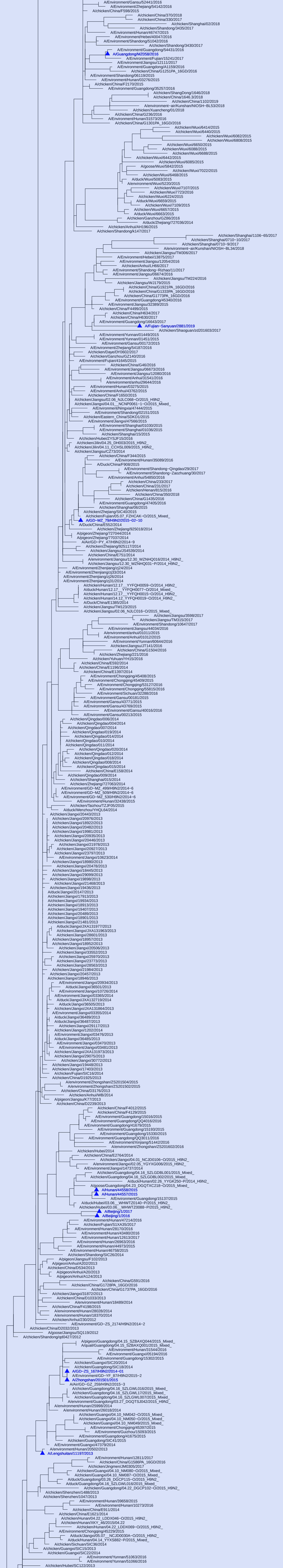

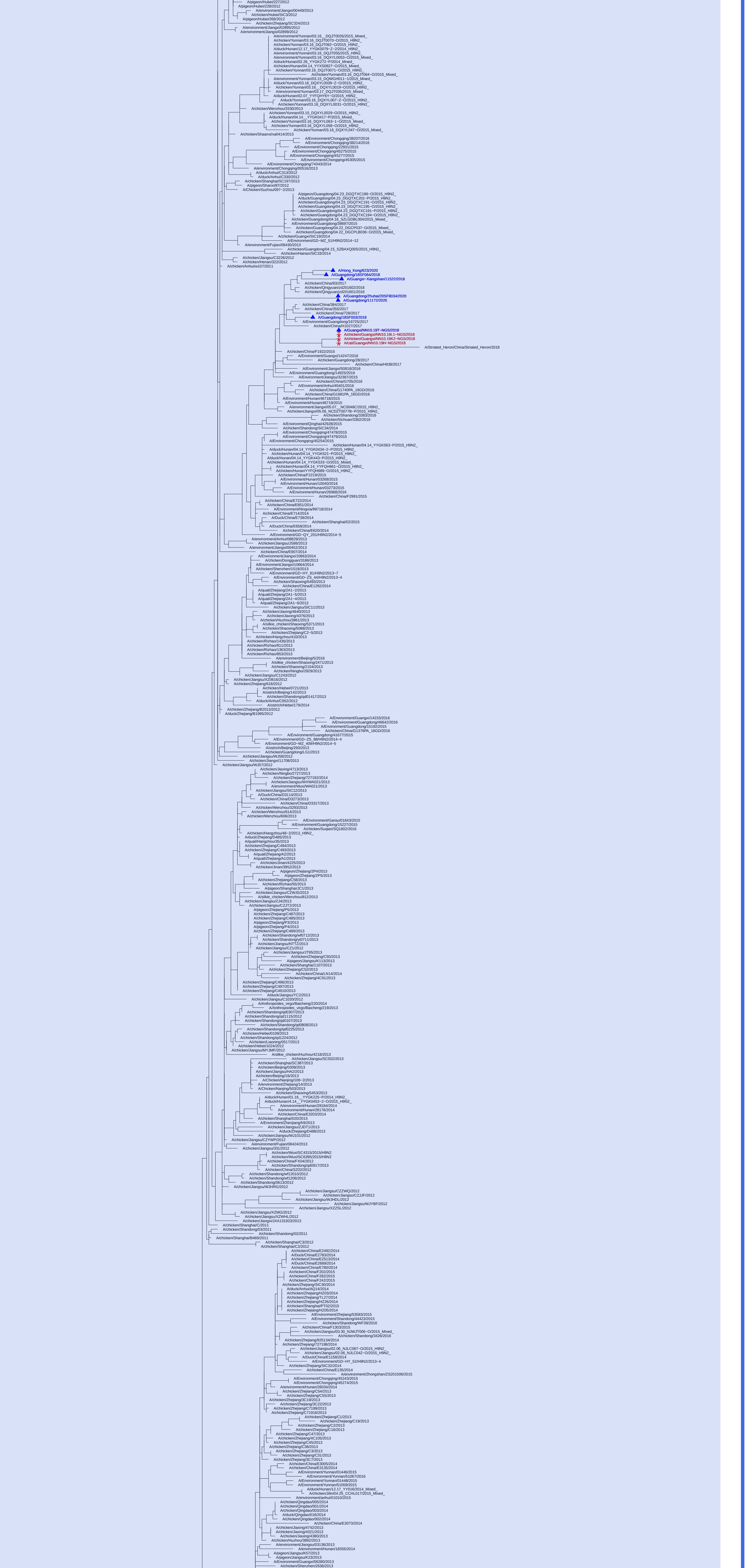
F98

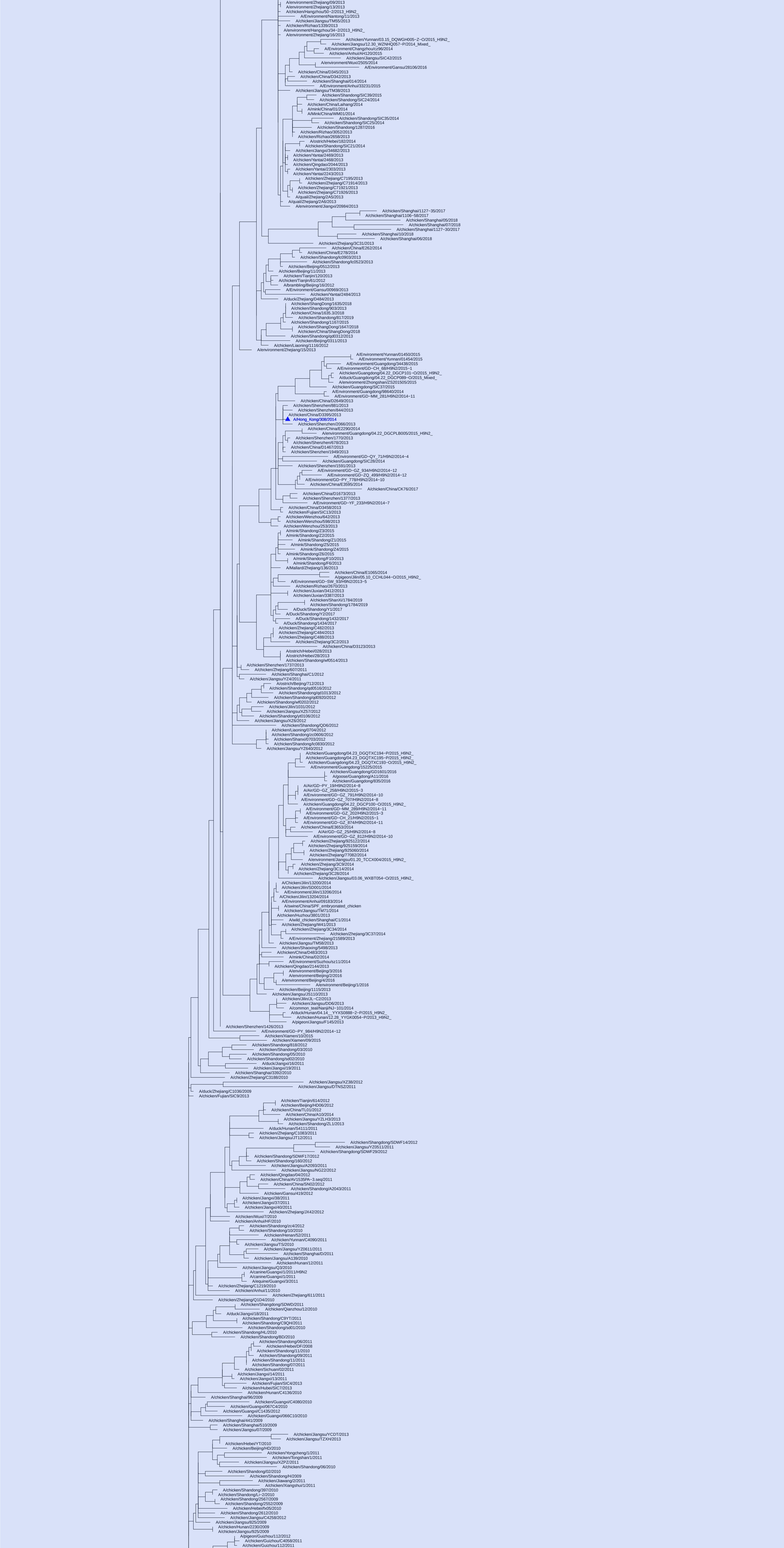

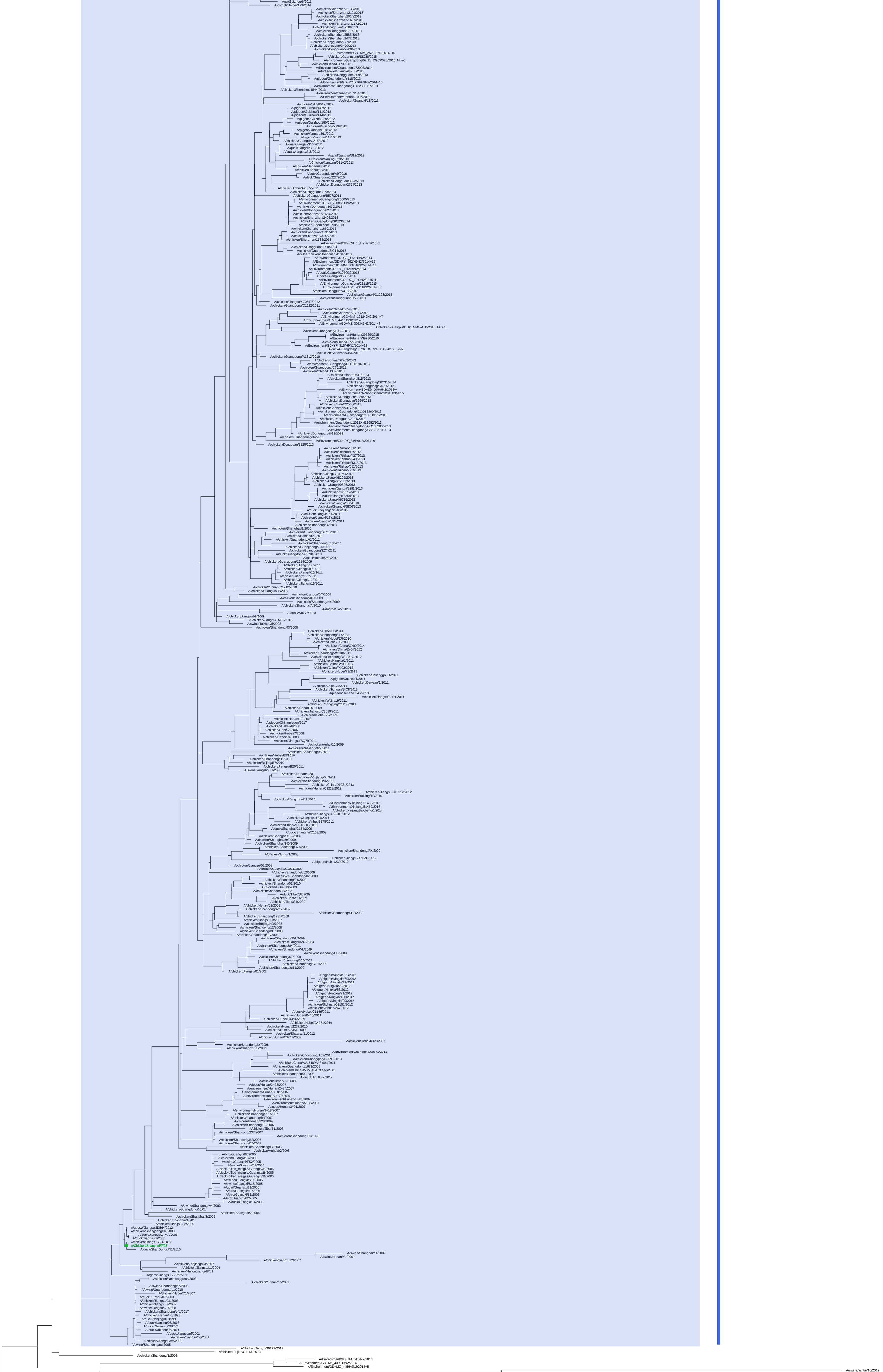

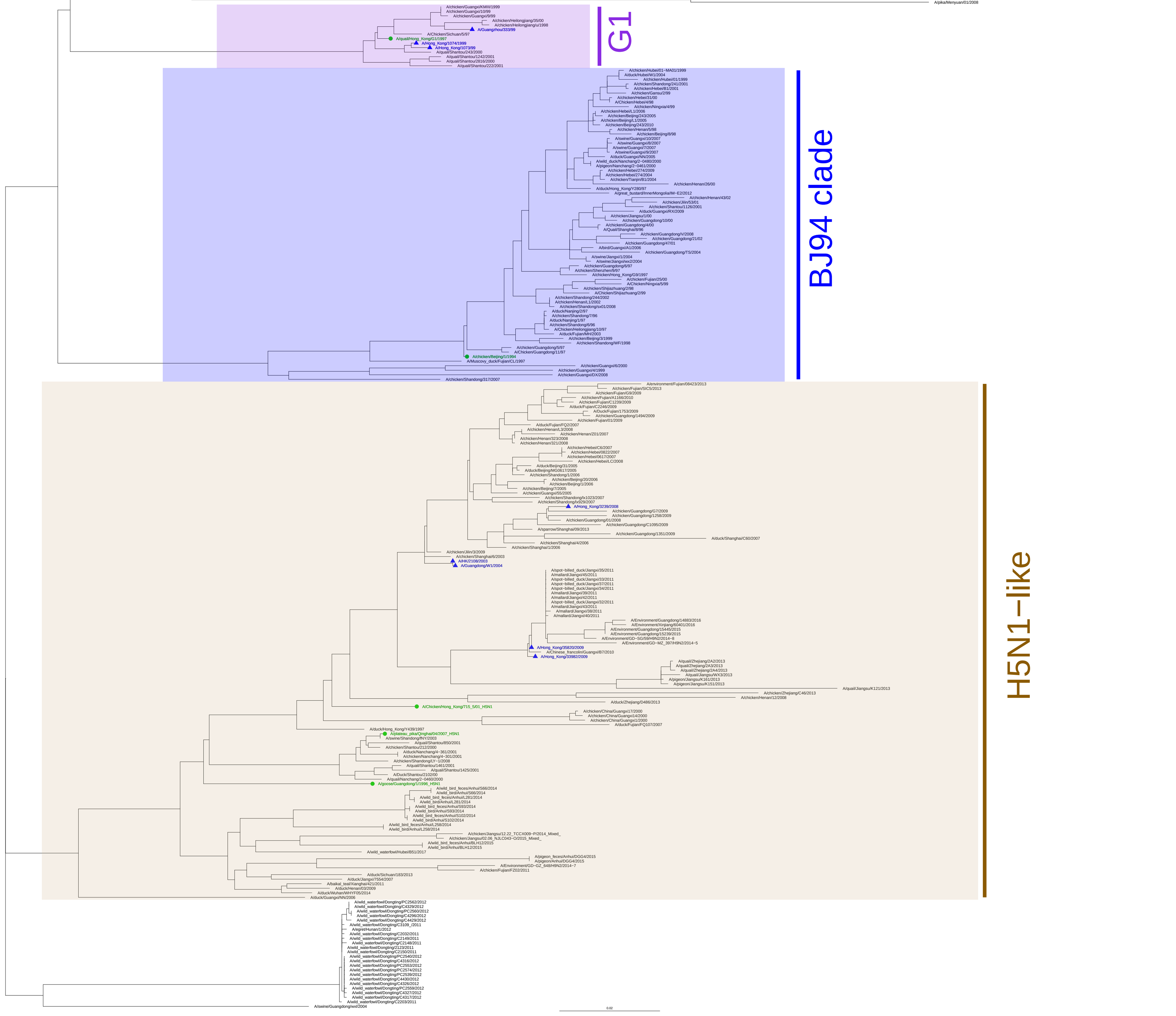

A/pika/Menyuan/01/2008
G1
A/chicken/Guangxi/KMIII/1999
A/chicken/Guangxi/10/99
A/chicken/Guangxi/9/99
A/chicken/Heilongjiang/35/00
A/chicken/Heilongjiang/u/1998
A/Guangzhou/333/99
A/Chicken/Sichuan/5/97
A/quail/Hong_Kong/G1/1997
A/Hong_Kong/1074/1999
A/Hong_Kong/1073/99
A/quail/Shantou/243/2000
A/quail/Shantou/1242/2001
A/quail/Shantou/2816/2000
A/quail/Shantou/222/2001
BJ94 clade
A/chicken/Hubei/01-MA01/1999
A/duck/Hubei/W1/2004
A/chicken/Hubei/01/1999
A/chicken/Shandong/241/2001
A/chicken/Hebei/B1/2001
A/chicken/Gansu/2/99
A/chicken/Hebei/31/00
A/Chicken/Hebei/4/98
A/chicken/Ningxia/4/99
A/chicken/Hebei/L1/2006
A/chicken/Beijing/243/2005
A/chicken/Beijing/L1/2005
A/chicken/Beijing/243/2010
A/chicken/Henan/5/98
A/chicken/Beijing/8/98
A/swine/Guangxi/10/2007
A/swine/Guangxi/8/2007
A/swine/Guangxi/7/2007
A/swine/Guangxi/9/2007
A/duck/Guangxi/NN/2005
A/wild_duck/Nanchang/2-0480/2000
A/pigeon/Nanchang/2-0461/2000
A/chicken/Hebei/274/2009
A/chicken/Hebei/274/2004
A/chicken/Tianjin/B1/2004
A/chicken/Henan/26/00
A/duck/Hong_Kong/Y280/97
A/great_bustard/InnerMongolia/IM-E2/2012
A/chicken/Henan/43/02
A/chicken/Jilin/53/01
A/chicken/Shantou/1126/2001
A/duck/Guangxi/RX/2009
A/chicken/Jiangsu/1/00
A/chicken/Guangdong/10/00
A/chicken/Guangdong/4/00
A/Quail/Shanghai/8/96
A/chicken/Guangdong/V/2008
A/chicken/Guangdong/21/02
A/chicken/Guangdong/47/01
A/bird/Guangxi/A1/2006
A/chicken/Guangdong/TS/2004
A/swine/Jiangxi/1/2004
A/swine/Jiangxi/wx2/2004
A/chicken/Guangdong/6/97
A/chicken/Shenzhen/9/97
A/chicken/Hong_Kong/G9/1997
A/chicken/Fujian/25/00
A/chicken/Ningxia/5/99
A/chicken/Shijiazhuang/2/98
A/Chicken/Shijiazhuang/2/99
A/chicken/Shandong/244/2002
A/chicken/Henan/L1/2002
A/chicken/Shandong/sx01/2008
A/duck/Nanjing/2/97
A/chicken/Shandong/7/96
A/duck/Nanjing/1/97
A/chicken/Shandong/6/96
A/Chicken/Heilongjiang/10/97
A/duck/Fujian/MH/2003
A/chicken/Beijing/3/1999
A/chicken/Shandong/WF/1998
A/chicken/Guangdong/5/97
A/Chicken/Guangdong/11/97
A/chicken/Beijing/1/1994
A/Muscovy_duck/Fujian/CL/1997
A/chicken/Guangxi/6/2000
A/chicken/Guangxi/4/1999
A/chicken/Guangxi/DX/2008
A/chicken/Shandong/317/2007
H5N1-like
A/environment/Fujian/08423/2013
A/chicken/Fujian/SIC5/2013
A/chicken/Fujian/G9/2009
A/chicken/Fujian/A1166/2010
A/chicken/Fujian/C1239/2009
A/duck/Fujian/C2246/2009
A/Duck/Fujian/1753/2009
A/chicken/Guangdong/1494/2009
A/chicken/Fujian/01/2009
A/duck/Fujian/FQ2/2007
A/chicken/Henan/L3/2008
A/chicken/Henan/Z01/2007
A/chicken/Henan/323/2008
A/chicken/Henan/321/2008
A/chicken/Hebei/C6/2007
A/chicken/Hebei/0822/2007
A/chicken/Hebei/0617/2007
A/chicken/Hebei/LC/2008
A/duck/Beijing/31/2005
A/duck/Beijing/MG0617/2005
A/chicken/Shandong/1/2006
A/chicken/Beijing/20/2006
A/chicken/Beijing/1/2006
A/chicken/Beijing/7/2005
A/chicken/Guangxi/55/2005
A/chicken/Shandong/lx1023/2007
A/chicken/Shandong/lx929/2007
A/Hong_Kong/3239/2008
A/chicken/Guangdong/G7/2009
A/chicken/Guangdong/1258/2009
A/chicken/Guangdong/01/2008
A/chicken/Guangdong/C1095/2009
A/sparrow/Shanghai/09/2013
A/chicken/Guangdong/1351/2009
A/duck/Shanghai/C60/2007
A/chicken/Shanghai/4/2006
A/chicken/Shanghai/1/2006
A/chicken/Jilin/3/2009
A/chicken/Shanghai/6/2003
A/HK/2108/2003
A/Guangdong/W1/2004
A/spot-billed_duck/Jiangxi/35/2011
A/mallard/Jiangxi/45/2011
A/spot-billed_duck/Jiangxi/33/2011
A/spot-billed_duck/Jiangxi/37/2011
A/spot-billed_duck/Jiangxi/34/2011
A/mallard/Jiangxi/39/2011
A/mallard/Jiangxi/42/2011
A/spot-billed_duck/Jiangxi/32/2011
A/mallard/Jiangxi/43/2011
A/mallard/Jiangxi/38/2011
A/mallard/Jiangxi/40/2011
A/Environment/Guangdong/14883/2016
A/Environment/Xinjiang/60401/2016
A/Environment/Guangdong/15445/2015
A/Environment/Guangdong/15239/2015
A/Environment/GD-SG/59/H9N2/2014-8
A/Environment/GD-MZ_397/H9N2/2014-5
A/Hong_Kong/35820/2009
A/Chinese_francolin/Guangxi/B7/2010
A/Hong_Kong/33982/2009
A/quail/Zhejiang/2A2/2013
A/quail/Zhejiang/2A3/2013
A/quail/Zhejiang/2A4/2013
A/quail/Jiangsu/WX3/2013
A/pigeon/Jiangsu/K161/2013
A/pigeon/Jiangsu/K151/2013
A/quail/Jiangsu/K121/2013
A/chicken/Zhejiang/C46/2013
A/chicken/Henan/12/2008
A/duck/Zhejiang/D486/2013
A/Chicken/Hong_Kong/715_5/01_H5N1
A/chicken/China/Guangxi17/2000
A/chicken/China/Guangxi14/2000
A/chicken/China/Guangxi1/2000
A/duck/Fujian/FQ107/2007
A/duck/Hong_Kong/Y439/1997
A/plateau_pika/Qinghai/04/2007_H5N1
A/swine/Shandong/FNY/2003
A/quail/Shantou/850/2001
A/chicken/Shantou/212/2000
A/duck/Nanchang/4-361/2001
A/chicken/Nanchang/4-301/2001
A/chicken/Shandong/LY-1/2008
A/quail/Shantou/1461/2001
A/quail/Shantou/1425/2001
A/Duck/Shantou/2102/00
A/quail/Nanchang/2-0460/2000
A/goose/Guangdong/1/1996_H5N1
A/wild_bird_feces/Anhui/S66/2014
A/wild_bird/Anhui/S66/2014
A/wild_bird_feces/Anhui/L281/2014
A/wild_bird/Anhui/L281/2014
A/wild_bird_feces/Anhui/S93/2014
A/wild_bird/Anhui/S93/2014
A/wild_bird_feces/Anhui/S102/2014
A/wild_bird/Anhui/S102/2014
A/wild_bird_feces/Anhui/L258/2014
A/wild_bird/Anhui/L258/2014
A/chicken/Jiangsu/12.22_TCCX009-P/2014_Mixed_
A/chicken/Jiangsu/02.06_NJLC043-O/2015_Mixed_
A/wild_bird_feces/Anhui/BLH12/2015
A/wild_bird/Anhui/BLH12/2015
A/wild_waterfowl/Hubei/B51/2017
A/pigeon_feces/Anhui/DGG4/2015
A/pigeon/Anhui/DGG4/2015
A/Environment/GD-GZ_648/H9N2/2014-7
A/chicken/Fujian/FZ02/2011
A/duck/Sichuan/183/2013
A/duck/Jiangxi/7554/2007
A/baikal_teal/Xianghai/421/2011
A/duck/Henan/03/2009
A/duck/Wuhan/WHYF05/2014
A/duck/Guangxi/NN/2006
A/wild_waterfowl/Dongting/PC2562/2012
A/wild_waterfowl/Dongting/C4329/2012
A/wild_waterfowl/Dongting/PC2560/2012
A/wild_waterfowl/Dongting/C4296/2012
A/wild_waterfowl/Dongting/C4429/2012
A/wild_waterfowl/Dongting/C3109_/2011
A/egret/Hunan/1/2012
A/wild_waterfowl/Dongting/C2032/2011
A/wild_waterfowl/Dongting/C2149/2011
A/wild_waterfowl/Dongting/C2148/2011
A/wild_waterfowl/Dongting/2123/2011
A/wild_waterfowl/Dongting/C2150/2011
A/wild_waterfowl/Dongting/PC2540/2012
A/wild_waterfowl/Dongting/C4316/2012
A/wild_waterfowl/Dongting/PC2553/2012
A/wild_waterfowl/Dongting/PC2574/2012
A/wild_waterfowl/Dongting/PC2539/2012
A/wild_waterfowl/Dongting/C4430/2012
A/wild_waterfowl/Dongting/C4326/2012
A/wild_waterfowl/Dongting/PC2559/2012
A/wild_waterfowl/Dongting/C4327/2012
A/wild_waterfowl/Dongting/C4317/2012
A/wild_waterfowl/Dongting/C2203/2011
A/swine/Guangdong/wxl/2004
0.02

(F) NP

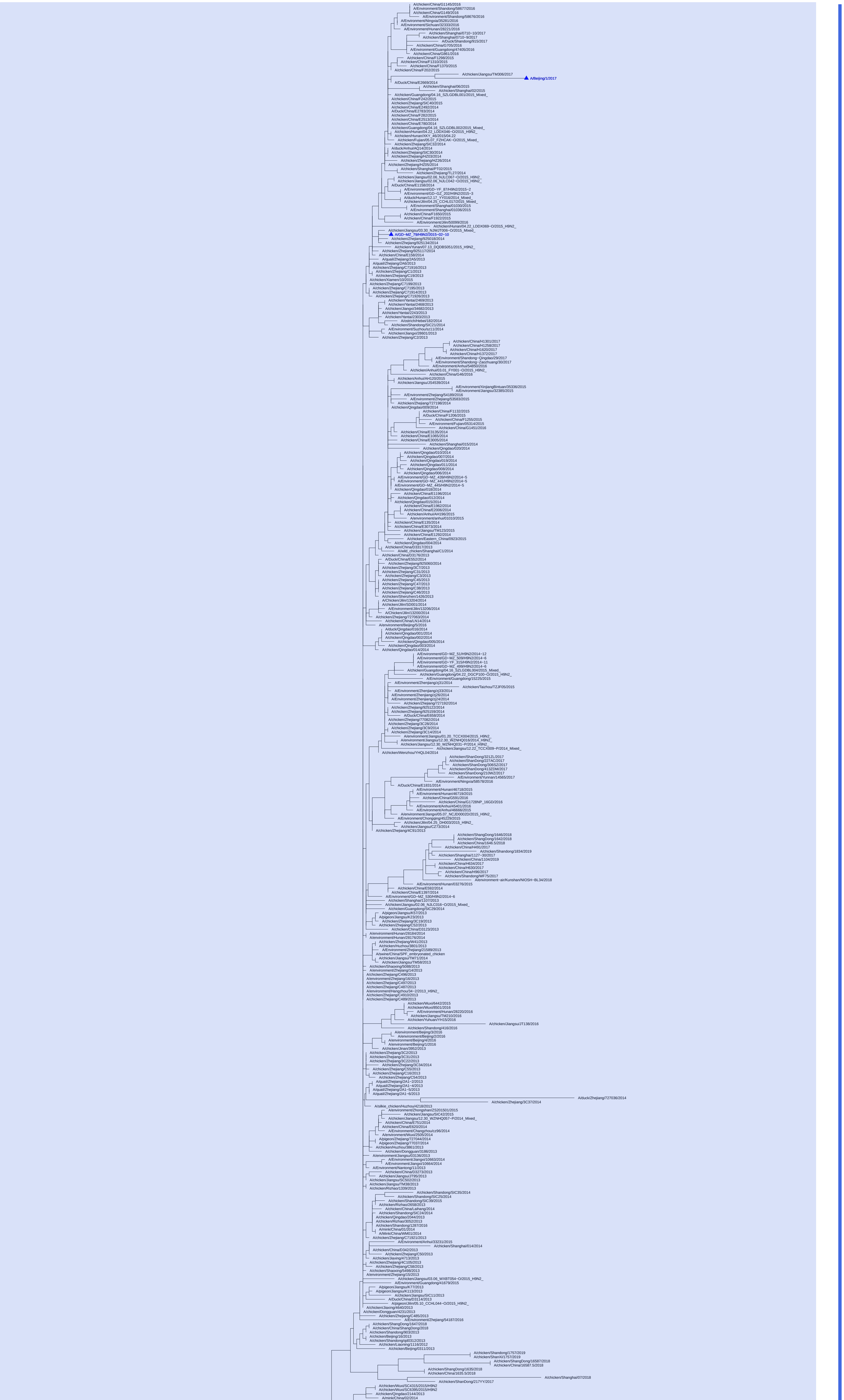

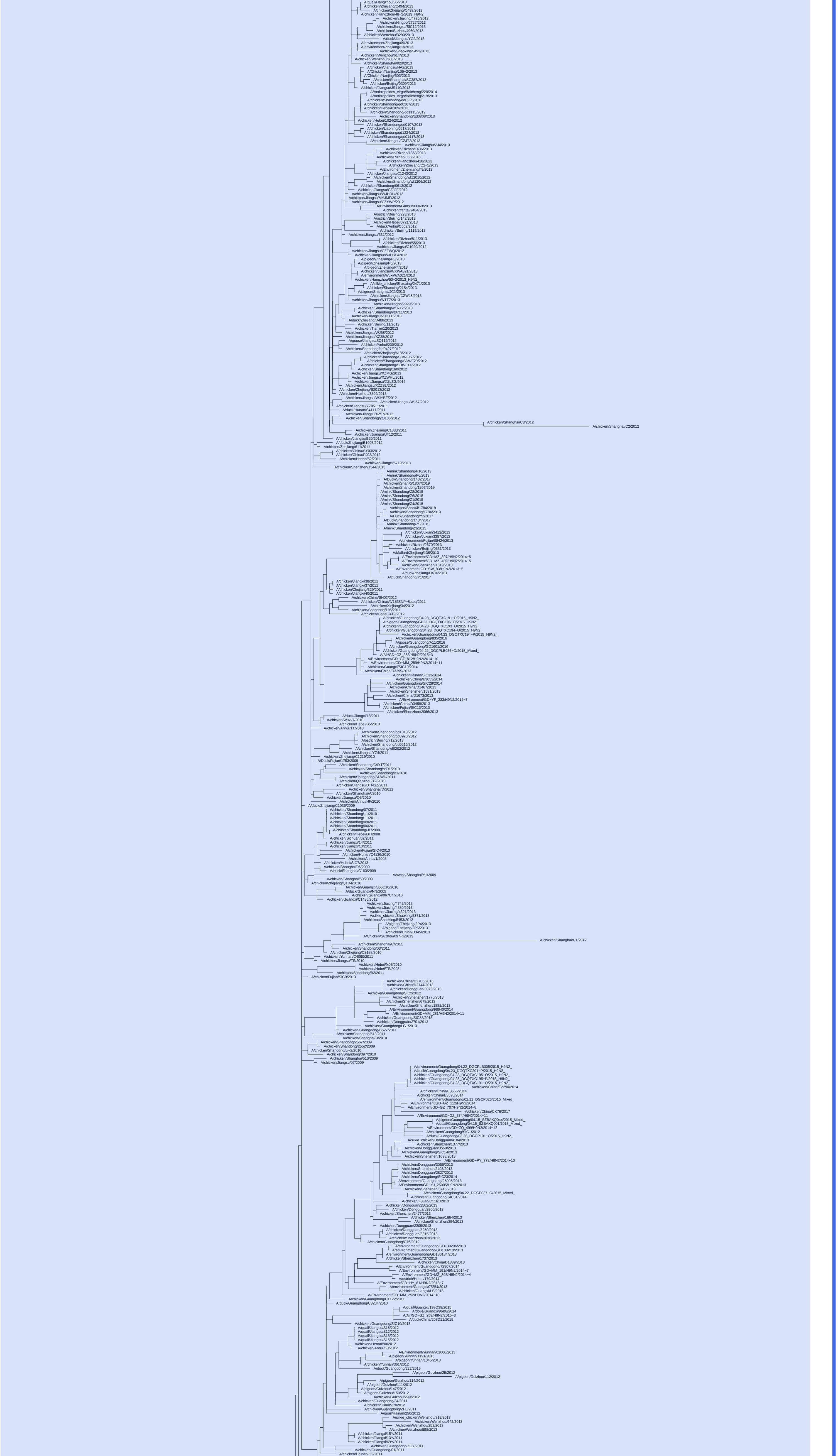
A/quail/Hangzhou/35/2013
A/chicken/Zhejiang/C494/2013
A/chicken/Zhejiang/C493/2013
A/chicken/Hangzhou/48-2/2013_H9N2
A/chicken/Jiaxing/4725/2013
A/chicken/Ningbo/2727/2013
A/chicken/Jiangsu/SIC12/2013
A/chicken/Suzhou/4960/2013
A/chicken/Wenzhou/3293/2013
A/duck/Jiangsu/YC2/2013
A/environment/Zhejiang/09/2013
A/environment/Zhejiang/13/2013
A/chicken/Shaoxing/5493/2013
A/chicken/Wenzhou/614/2013
A/chicken/Wenzhou/606/2013
A/chicken/Shanghai/020/2013
A/chicken/Jiangsu/HA2/2013
A/Chicken/Nanjing/106-2/2013
A/Chicken/Nanjing/5/03/2013
A/chicken/Shanghai/SC387/2013
A/chicken/Beijing/0309/2013
A/chicken/Jiangsu/JS10/2013
A/Anthropoides_virgo/Baicheng/220/2014
A/Anthropoides_virgo/Baicheng/219/2013
A/chicken/Shandong/qd0225/2013
A/chicken/Shandong/qd0307/2013
A/chicken/Hebei/0109/2013
A/chicken/Shandong/qd1115/2012
A/chicken/Shandong/qd0808/2013
A/chicken/Hebei/1024/2012
A/chicken/Shandong/qd0107/2013
A/chicken/Liaoning/0517/2013
A/chicken/Shandong/qd1224/2012
A/chicken/Shandong/qd01417/2013
A/chicken/Jiangsu/CZJT2/2013
A/chicken/Jiangsu/ZJ4/2013
A/chicken/Rizhao/1436/2013
A/chicken/Rizhao/1363/2013
A/chicken/Rizhao/853/2013
A/chicken/Hangzhou/410/2013
A/chicken/Zhejiang/C2-5/2013
A/Enviroment/Zhenjiang/h9/2013
A/chicken/Jiangsu/C1243/2012
A/chicken/Shandong/wf12010/2012
A/chicken/Shandong/wf1206/2012
A/chicken/Shandong/0613/2012
A/chicken/Jiangsu/CZJJF/2012
A/chicken/Jiangsu/WJHDL/2012
A/chicken/Jiangsu/MYJMF/2012
A/chicken/Jiangsu/CZYWF/2012
A/Environment/Gansu/00969/2013
A/chicken/Yantai/2484/2013
A/ostrich/Beijing/293/2013
A/ostrich/Beijing/142/2013
A/chicken/Hebei/0721/2013
A/duck/Anhui/C652/2012
A/chicken/Beijing/1115/2013
A/chicken/Jiangsu/331/2012
A/chicken/Rizhao/811/2013
A/chicken/Rizhao/55/2013
A/chicken/Jiangsu/C1020/2012
A/chicken/Jiangsu/CZZWQ/2012
A/chicken/Jiangsu/HJHRG/2012
A/pigeon/Zhejiang/P3/2013
A/pigeon/Zhejiang/P5/2013
A/pigeon/Zhejiang/P4/2013
A/chicken/Jiangsu/WXWA021/2013
A/environment/Wuxi/WA021/2013
A/chicken/Hangzhou/50-2/2013_H9N2
A/silkie_chicken/Shaoxing/2471/2013
A/chicken/Shaoxing/2154/2013
A/pigeon/Shanghai/JC1/2013
A/chicken/Jiangsu/CZWJ5/2013
A/chicken/Jiangsu/NTTZ/2013
A/chicken/Ningbo/2929/2013
A/chicken/Shandong/wf0712/2013
A/chicken/Shandong/yt0711/2013
A/chicken/Jiangsu/ZJDT1/2013
A/duck/Zhejiang/D488/2013
A/chicken/Beijing/11/2013
A/chicken/Tianjin/120/2013
A/chicken/Jiangsu/WJ58/2012
A/chicken/Jiangsu/XZ38/2012
A/goose/Jiangsu/SQ119/2012
A/chicken/Anhui/230/2012
A/chicken/Shandong/qd0427/2012
A/chicken/Zhejiang/618/2012
A/chicken/Shandong/SDWF17/2012
A/chicken/Shangdong/SDWF29/2012
A/chicken/Shangdong/SDWF14/2012
A/chicken/Shandong/160/2012
A/chicken/Jiangsu/XZMG/2012
A/chicken/Jiangsu/XZWHL/2012
A/chicken/Jiangsu/XZLZG/2012
A/chicken/Jiangsu/XZZSL/2012
A/chicken/Zhejiang/B2013/2012
A/chicken/Huzhou/3892/2013
A/chicken/Jiangsu/WJYBF/2012
A/chicken/Jiangsu/WJ57/2012
A/chicken/Jiangsu/YZ0511/2011
A/duck/Hunan/S4111/2011
A/chicken/Jiangsu/XZ57/2012
A/chicken/Shandong/yt0106/2012
A/chicken/Shanghai/C3/2012
A/chicken/Shanghai/C2/2012
A/chicken/Zhejiang/C1083/2011
A/chicken/Jiangsu/JT12/2011
A/chicken/Jiangsu/B20/2011
A/duck/Zhejiang/B1995/2012
A/chicken/Zhejiang/611/2011
A/chicken/China/SY03/2012
A/chicken/China/PJ03/2012
A/chicken/Henan/22/2011
A/chicken/Jiangxi/6719/2013
A/chicken/Shenzhen/1544/2013
A/mink/Shandong/F10/2013
A/mink/Shandong/F6/2013
A/Duck/Shandong/1432/2017
A/chicken/ShanXi/1807/2019
A/chicken/Shandong/1807/2019
A/mink/Shandong/Z2/2015
A/mink/Shandong/Z6/2015
A/mink/Shandong/Z1/2015
A/mink/Shandong/Z4/2015
A/chicken/ShanXi/1784/2019
A/chicken/Shandong/1784/2019
A/Duck/Shandong/Y2/2017
A/Duck/Shandong/1434/2017
A/mink/Shandong/Z5/2015
A/mink/Shandong/Z3/2015
A/chicken/Juxian/3412/2013
A/chicken/Juxian/3387/2013
A/environment/Fujian/08424/2013
A/chicken/Rizhao/2670/2013
A/chicken/Beijing/0331/2013
A/Mallard/Zhejiang/136/2013
A/Environment/GD-MZ_397/H9N2/2014-5
A/Environment/GD-MZ_409/H9N2/2014-5
A/chicken/Shenzhen/1519/2013
A/Environment/GD-SW_93/H9N2/2013-5
A/duck/Zhejiang/D484/2013
A/Duck/Shandong/Y1/2017
A/chicken/Jiangxi/38/2011
A/chicken/Jiangxi/37/2011
A/chicken/Zhejiang/329/2011
A/chicken/Jiangxi/40/2011
A/chicken/China/SN02/2012
A/chicken/China/AV1535NP-5.seq/2011
A/chicken/Xinjiang/34/2012
A/chicken/Shandong/196/2011
A/chicken/Gansu/419/2012
A/chicken/Guangdong/04.23_DGQTXC191-P/2015_H9N2_
A/pigeon/Guangdong/04.23_DGQTXC196-O/2015_H9N2_
A/chicken/Guangdong/04.23_DGQTXC193-O/2015_H9N2_
A/chicken/Guangdong/04.23_DGQTXC194-O/2015_H9N2_
A/chicken/Guangdong/04.23_DGQTXC194-P/2015_H9N2_
A/chicken/Guangdong/835/2016
A/goose/Guangdong/A11/2016
A/chicken/Guangdong/GD1601/2016
A/chicken/Guangdong/04.22_DGCPLB036-O/2015_Mixed_
A/Air/GD-GZ_258/H9N2/2015-3
A/Environment/GD-GZ_812/H9N2/2014-10
A/Environment/GD-MM_289/H9N2/2014-11
A/chicken/Guangxi/SIC19/2014
A/chicken/China/D3395/2013
A/chicken/Hainan/SIC33/2014
A/chicken/China/E3653/2014
A/chicken/Guangdong/SIC28/2014
A/chicken/China/D1467/2013
A/chicken/Shenzhen/1591/2013
A/chicken/China/D1673/2013
A/Environment/GD-YF_233/H9N2/2014-7
A/chicken/China/D3458/2013
A/chicken/Fujian/SIC13/2013
A/chicken/Shenzhen/2066/2013
A/duck/Jiangxi/18/2011
A/chicken/Wuxi/7/2010
A/chicken/Hebei/B5/2010
A/chicken/Anhui/11/2010
A/chicken/Shandong/qd1013/2012
A/chicken/Shandong/qd0920/2012
A/ostrich/Beijing/712/2013
A/chicken/Shandong/qd0516/2012
A/chicken/Shandong/wf0202/2012
A/chicken/Jiangsu/YZ4/2011
A/chicken/Zhejiang/C1219/2010
A/Duck/Fujian/1753/2009
A/chicken/Shandong/C9YT/2011
A/chicken/Shandong/sd01/2010
A/chicken/Shandong/B1/2010
A/chicken/Shangdong/SDWD/2011
A/chicken/Qianzhou/12/2010
A/chicken/Jiangsu/DTNSZ/2011
A/chicken/Shanghai/D/2011
A/chicken/Shanghai/A/2010
A/chicken/Jiangsu/Q3/2010
A/chicken/Anhui/HF/2010
A/duck/Zhejiang/C1036/2009
A/chicken/Shandong/07/2011
A/chicken/Shandong/11/2010
A/chicken/Shandong/11/2011
A/chicken/Shandong/09/2011
A/chicken/Shandong/06/2011
A/chicken/Shandong/JL/2008
A/chicken/Hebei/DF/2008
A/chicken/Sichuan/02/2011
A/chicken/Jiangxi/14/2011
A/chicken/Jiangxi/13/2011
A/chicken/Fujian/SIC4/2013
A/chicken/Hunan/C4136/2010
A/chicken/Anhui/1/2008
A/chicken/Hubei/SIC7/2013
A/chicken/Shanghai/96/2009
A/duck/Shanghai/C163/2009
A/swine/Shanghai/Y1/2009
A/chicken/Shanghai/50/2009
A/chicken/Zhejiang/Q1D4/2010
A/chicken/Guangxi/066C10/2010
A/duck/Guangxi/NN/2005
A/chicken/Guangxi/067C4/2010
A/chicken/Guangxi/C1435/2012
A/chicken/Jiaxing/4742/2013
A/chicken/Jiaxing/4380/2013
A/chicken/Jiaxing/4321/2013
A/silkie_chicken/Shaoxing/5371/2013
A/chicken/Shaoxing/5453/2013
A/pigeon/Zhejiang/2P4/2013
A/pigeon/Zhejiang/2P5/2013
A/chicken/China/D3345/2013
A/chicken/Suzhou/097-2/2013
A/chicken/Shanghai/C1/2012
A/chicken/Shanghai/C/2011
A/chicken/Shandong/03/2011
A/chicken/Zhejiang/C3188/2010
A/chicken/Yunnan/C4090/2011
A/chicken/Jiangsu/TS/2010
A/chicken/Hebei/fx05/2010
A/chicken/Hebei/TS/2008
A/chicken/Shandong/B2/2011
A/chicken/Fujian/SIC9/2013
A/chicken/China/D2703/2013
A/chicken/China/D2744/2013
A/chicken/Dongguan/3073/2013
A/chicken/Guangdong/SIC2/2012
A/chicken/Shenzhen/1770/2013
A/chicken/Shenzhen/678/2013
A/chicken/Shenzhen/1882/2013
A/Environment/Guangdong/98640/2014
A/Environment/GD-MM_281/H9N2/2014-11
A/chicken/Guangdong/SIC38/2015
A/chicken/Dongguan/2701/2013
A/chicken/Guangdong/LG1/2013
A/chicken/Guangdong/8527/2011
A/chicken/Shandong/513/2011
A/chicken/Shanghai/8/2010
A/chicken/Shandong/2567/2009
A/chicken/Shandong/2552/2009
A/chicken/Shandong/Li-2/2010
A/chicken/Shandong/397/2010
A/chicken/Shanghai/510/2009
A/chicken/Jiangsu/07/2009
A/environment/Guangdong/04.22_DGCPLB005/2015_H9N2_
A/duck/Guangdong/04.23_DGQTXC201-P/2015_H9N2_
A/chicken/Guangdong/04.23_DGQTXC195-O/2015_H9N2_
A/chicken/Guangdong/04.23_DGQTXC195-P/2015_H9N2_
A/chicken/Guangdong/04.23_DGQTXC191-O/2015_H9N2_
A/chicken/China/E2290/2014
A/chicken/China/E3555/2014
A/chicken/China/E3595/2014
A/environment/Guangdong/02.11_DGCP026/2015_Mixed_
A/Environment/GD-GZ_112/H9N2/2014
A/Environment/GD-GZ_707/H9N2/2014-8
A/chicken/China/CK76/2017
A/Environment/GD-GZ_874/H9N2/2014-11
A/pigeon/Guangdong/04.15_SZBAXQ044/2015_Mixed_
A/quail/Guangdong/04.15_SZBAXQ001/2015_Mixed_
A/Environment/GD-ZQ_408/H9N2/2014-12
A/chicken/Guangdong/SIC1/2012
A/duck/Guangdong/03.26_DGCP101-O/2015_H9N2_
A/silkie_chicken/Dongguan/4184/2013
A/chicken/Shenzhen/1377/2013
A/chicken/Dongguan/3550/2013
A/chicken/Guangdong/SIC14/2013
A/chicken/Shenzhen/1098/2013
A/Environment/GD-PY_778/H9N2/2014-10
A/chicken/Dongguan/3056/2013
A/chicken/Shenzhen/2403/2013
A/chicken/Dongguan/2827/2013
A/chicken/Guangdong/SIC23/2014
A/environment/Guangdong/25005/2013
A/Environment/GD-YJ_25005/H9N2/2013
A/chicken/Shenzhen/3745/2013
A/chicken/Guangdong/04.22_DGCP037-O/2015_Mixed_
A/chicken/Guangdong/SIC31/2014
A/chicken/Fujian/C1161/2013
A/chicken/Dongguan/3562/2013
A/chicken/Dongguan/2900/2013
A/chicken/Shenzhen/2477/2013
A/chicken/Shenzhen/1664/2013
A/chicken/Shenzhen/354/2013
A/chicken/Dongguan/2309/2013
A/chicken/Dongguan/3250/2013
A/chicken/Dongguan/3315/2013
A/chicken/Shenzhen/2636/2013
A/chicken/Guangdong/C76/2012
A/environment/Guangdong/GD130206/2013
A/environment/Guangdong/GD130210/2013
A/environment/Guangdong/GD130184/2013
A/chicken/Shenzhen/1737/2013
A/chicken/China/D1389/2013
A/Environment/Guangdong/72907/2014
A/Environment/GD-MM_191/H9N2/2014-7
A/Environment/GD-MZ_308/H9N2/2014-4
A/ostrich/Hebei/179/2014
A/Environment/GD-HY_81/H9N2/2013-7
A/environment/Guangxi/07254/2013
A/chicken/Guangxi/LS/2013
A/Environment/GD-MM_252/H9N2/2014-10
A/chicken/Guangdong/C1122/2011
A/duck/Guangdong/C3204/2010
A/quail/Guangxi/198Q39/2015
A/dove/Guangxi/9688/2014
A/Air/GD-GZ_259/H9N2/2015-3
A/duck/China/208D11/2015
A/chicken/Guangdong/SIC10/2013
A/quail/Jiangsu/S16/2012
A/quail/Jiangsu/S12/2012
A/quail/Jiangsu/S18/2012
A/quail/Jiangsu/S15/2012
A/chicken/Henan/90/2012
A/chicken/Anhui/63/2012
A/Environment/Yunnan/01006/2013
A/pigeon/Yunnan/1191/2013
A/pigeon/Yunnan/1045/2013
A/chicken/Yunnan/361/2013
A/duck/Guangdong/222/2015
A/pigeon/Guizhou/29/2012
A/pigeon/Guizhou/112/2012
A/pigeon/Guizhou/114/2012
A/pigeon/Guizhou/111/2012
A/pigeon/Guizhou/147/2012
A/pigeon/Guizhou/150/2012
A/chicken/Guizhou/299/2012
A/chicken/Guangdong/34/2011
A/chicken/Jilin/0519/2012
A/chicken/Guangdong/ZHJ/2011
A/quail/Hainan/250/2012
A/silkie_chicken/Wenzhou/812/2013
A/chicken/Wenzhou/642/2013
A/chicken/Wenzhou/253/2013
A/chicken/Wenzhou/598/2013
A/chicken/Jiangxi/15Y/2011
A/chicken/Jiangxi/13Y/2011
A/chicken/Jiangxi/69Y/2011
A/chicken/Guangdong/ZCY/2011
A/chicken/Guangdong/01/2011
A/chicken/Hainan/22/2011

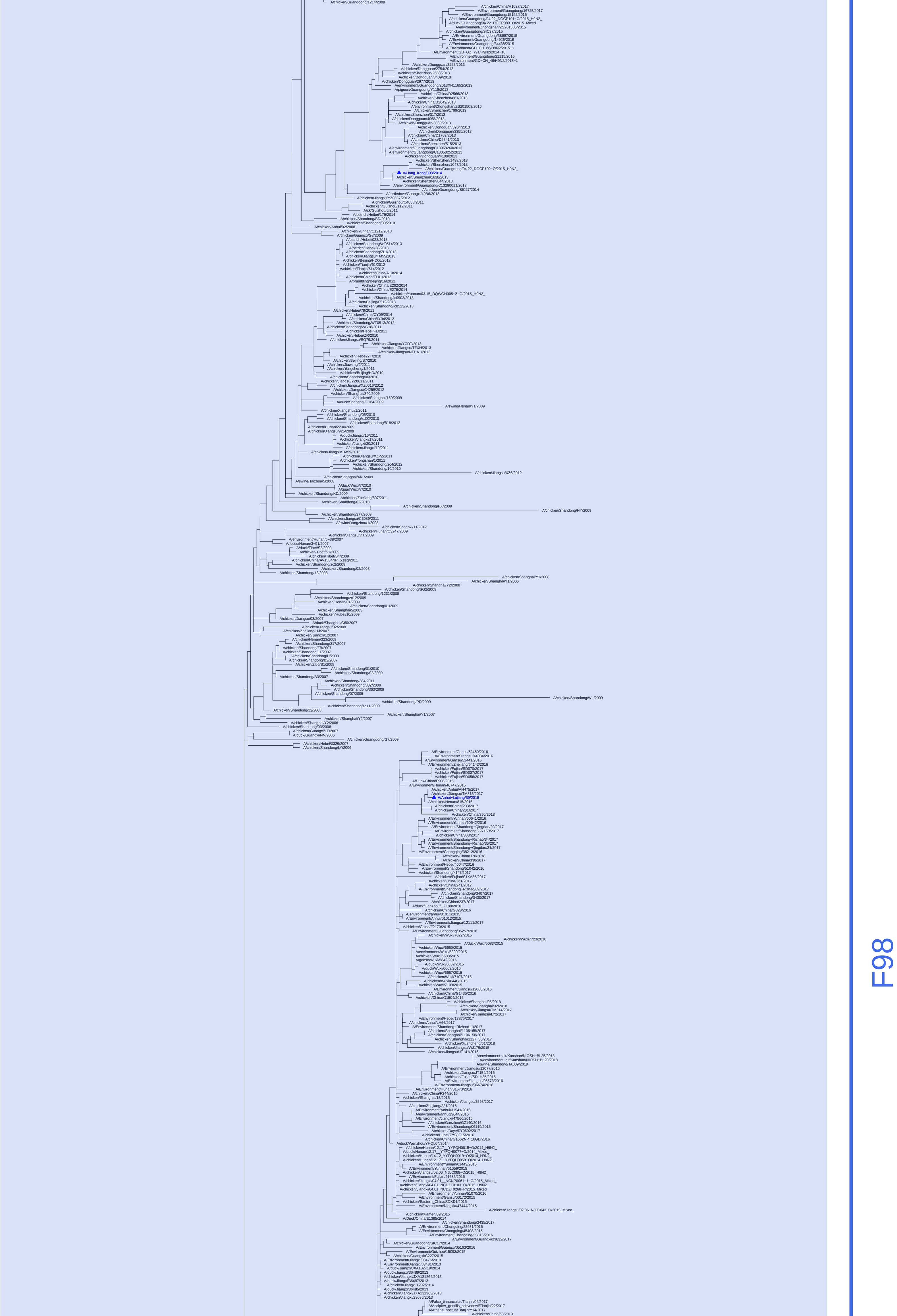
F98

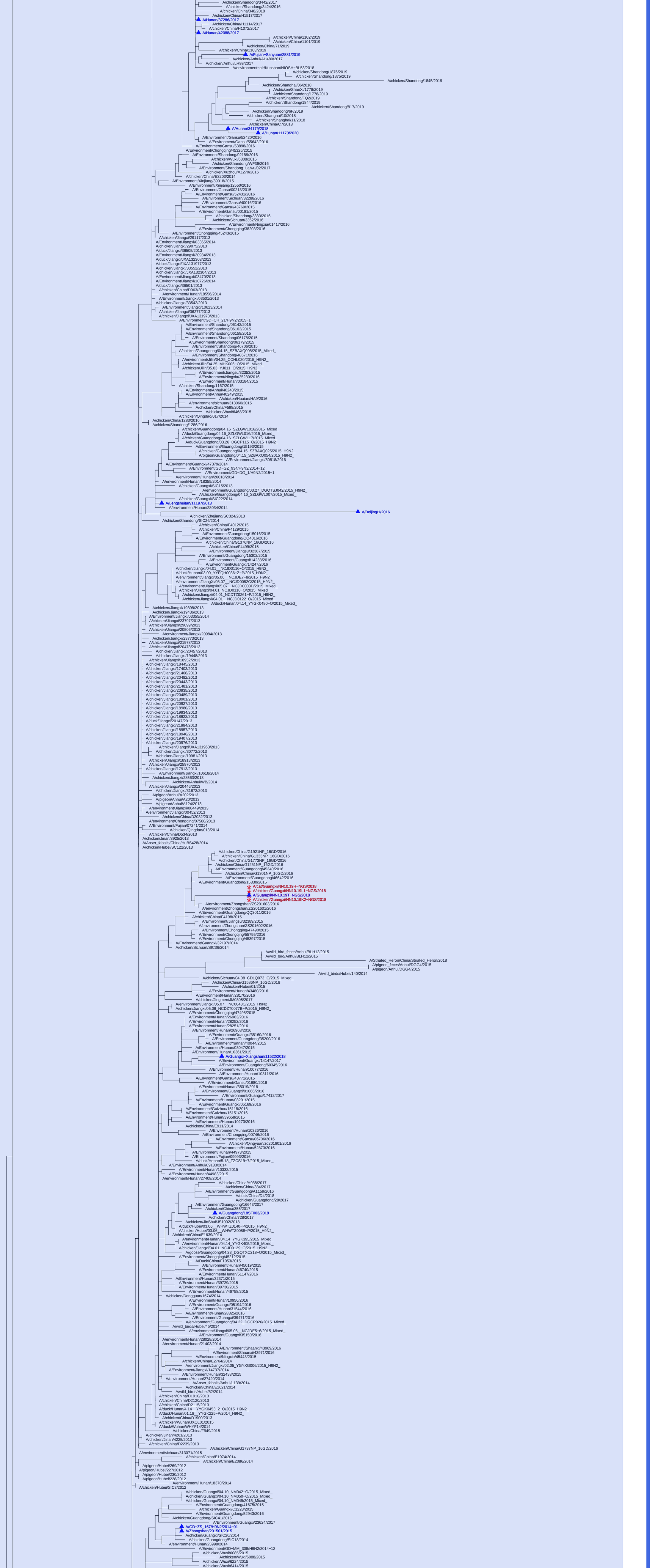

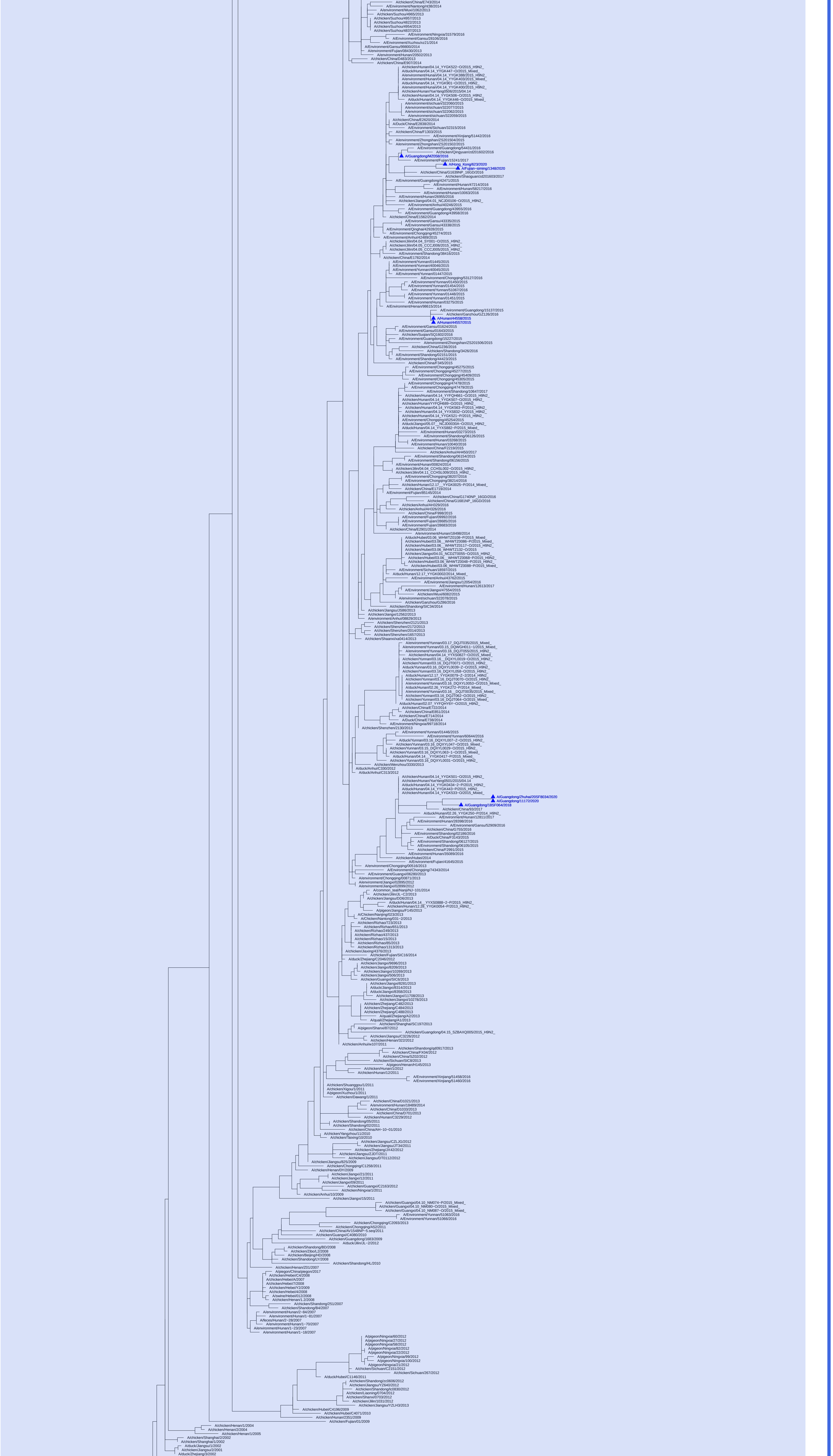

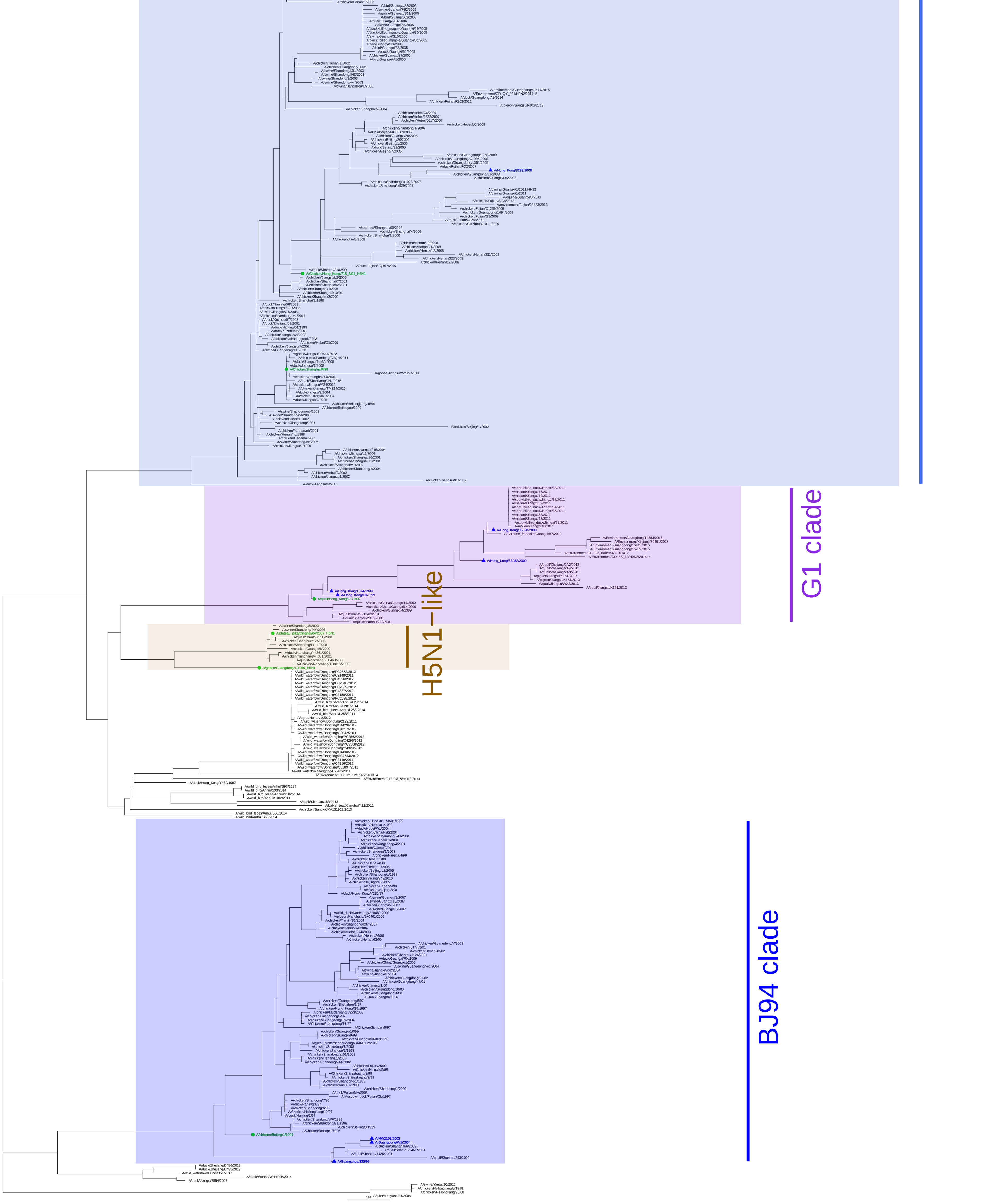

G1 clade
H5N1-like
BJ94 clade

(G) MP

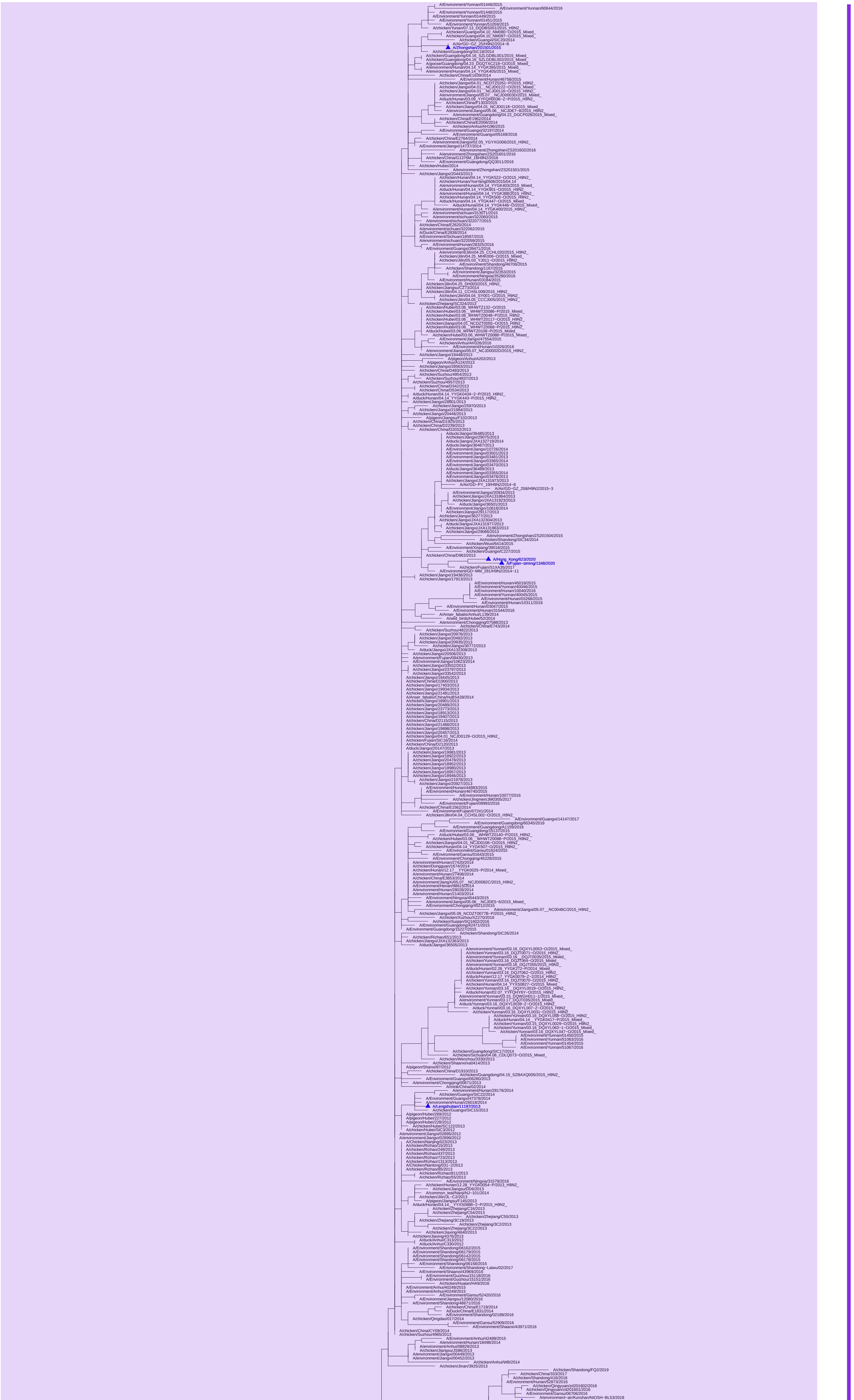

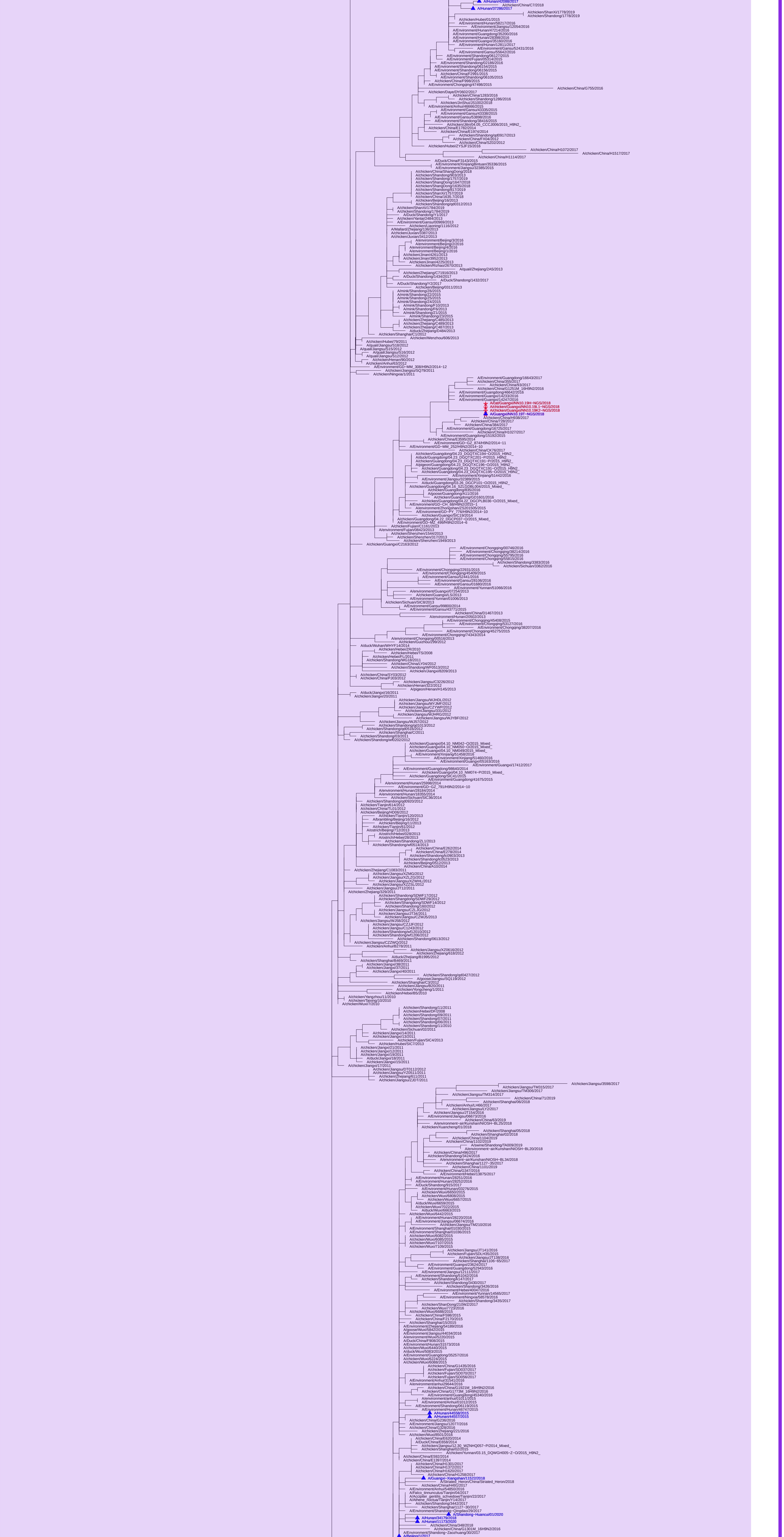

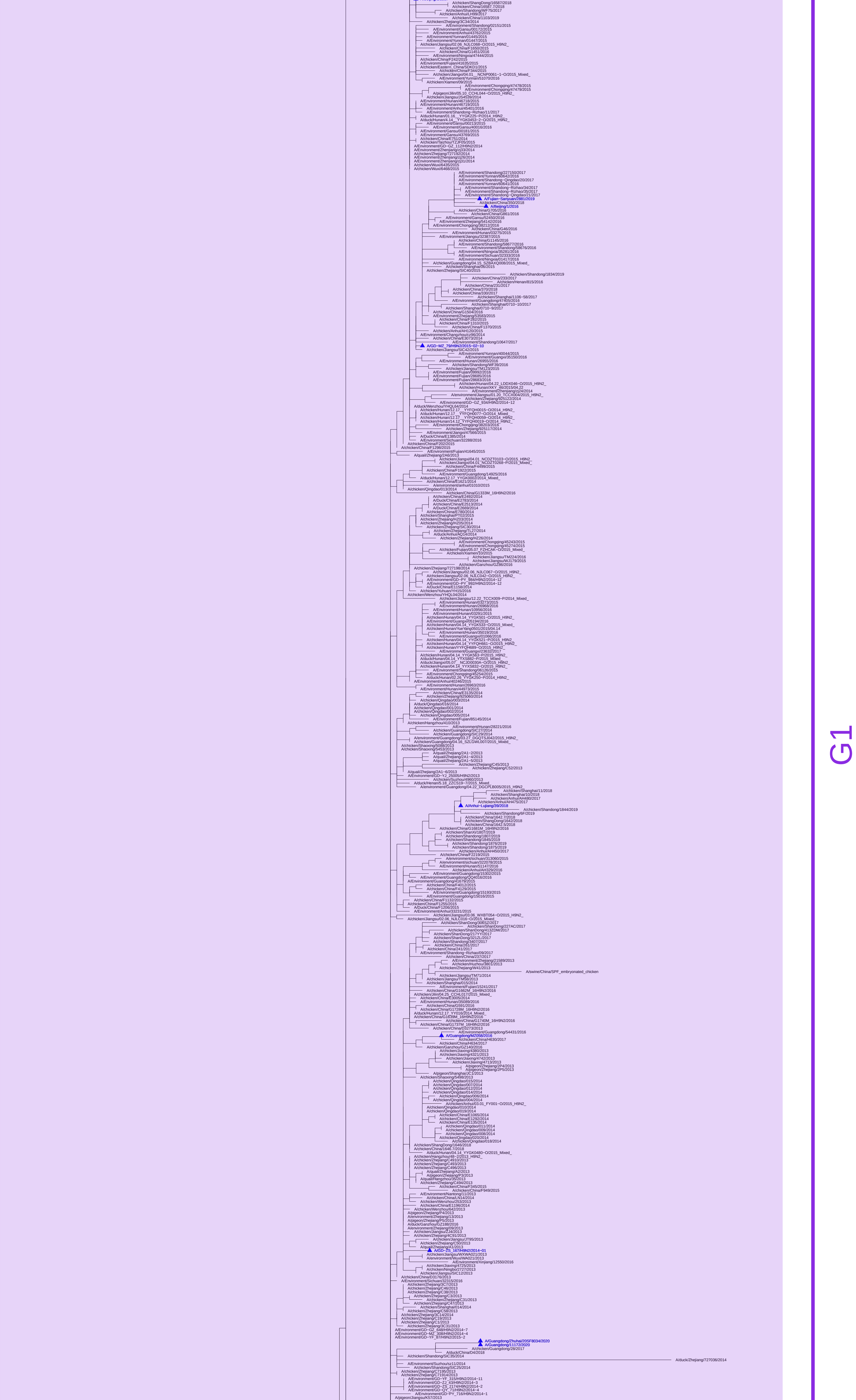
G1

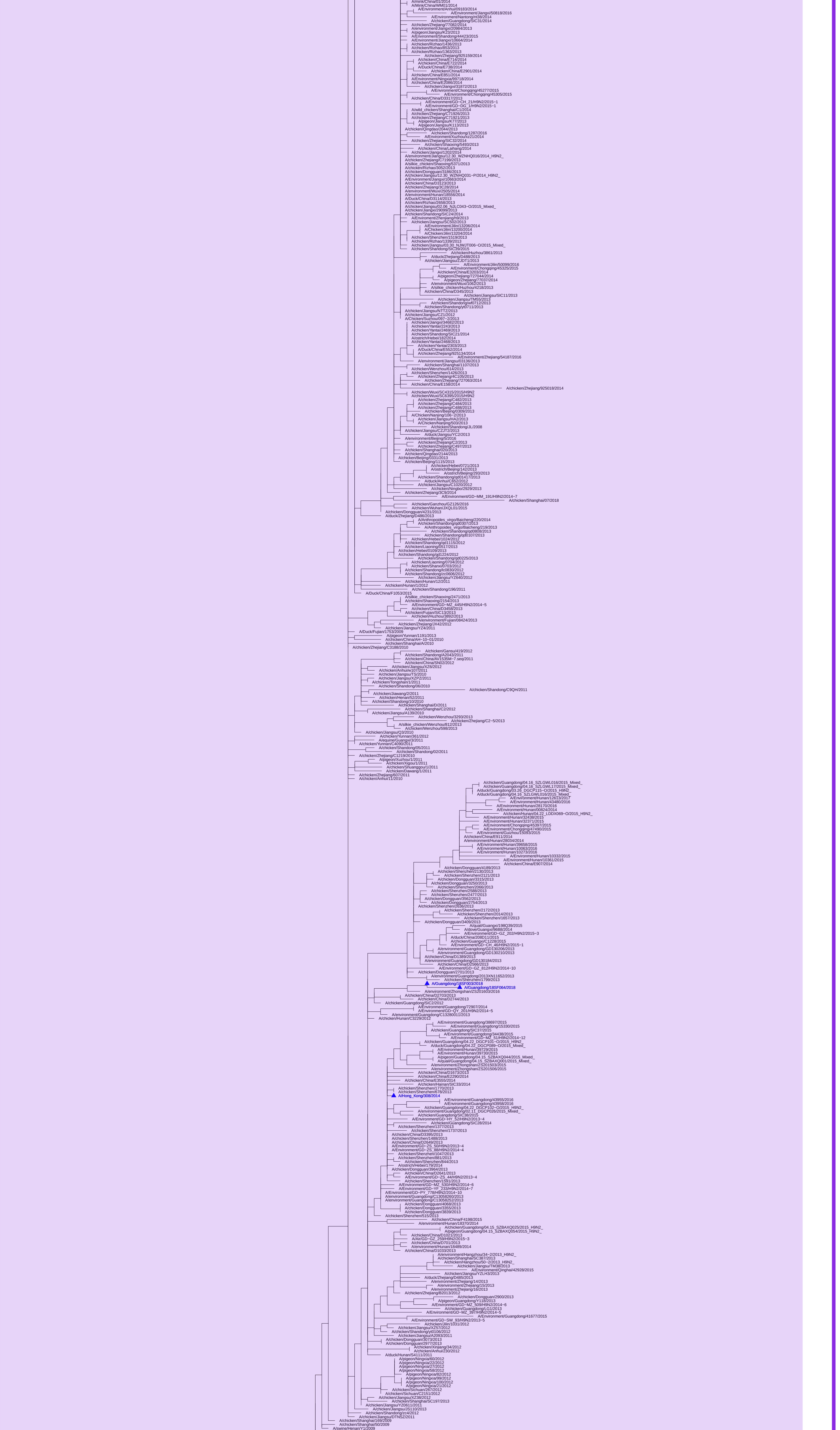

A/Guangdong/18SF003/2018
A/Guangdong/18SF064/2018
A/Hong_Kong/308/2014

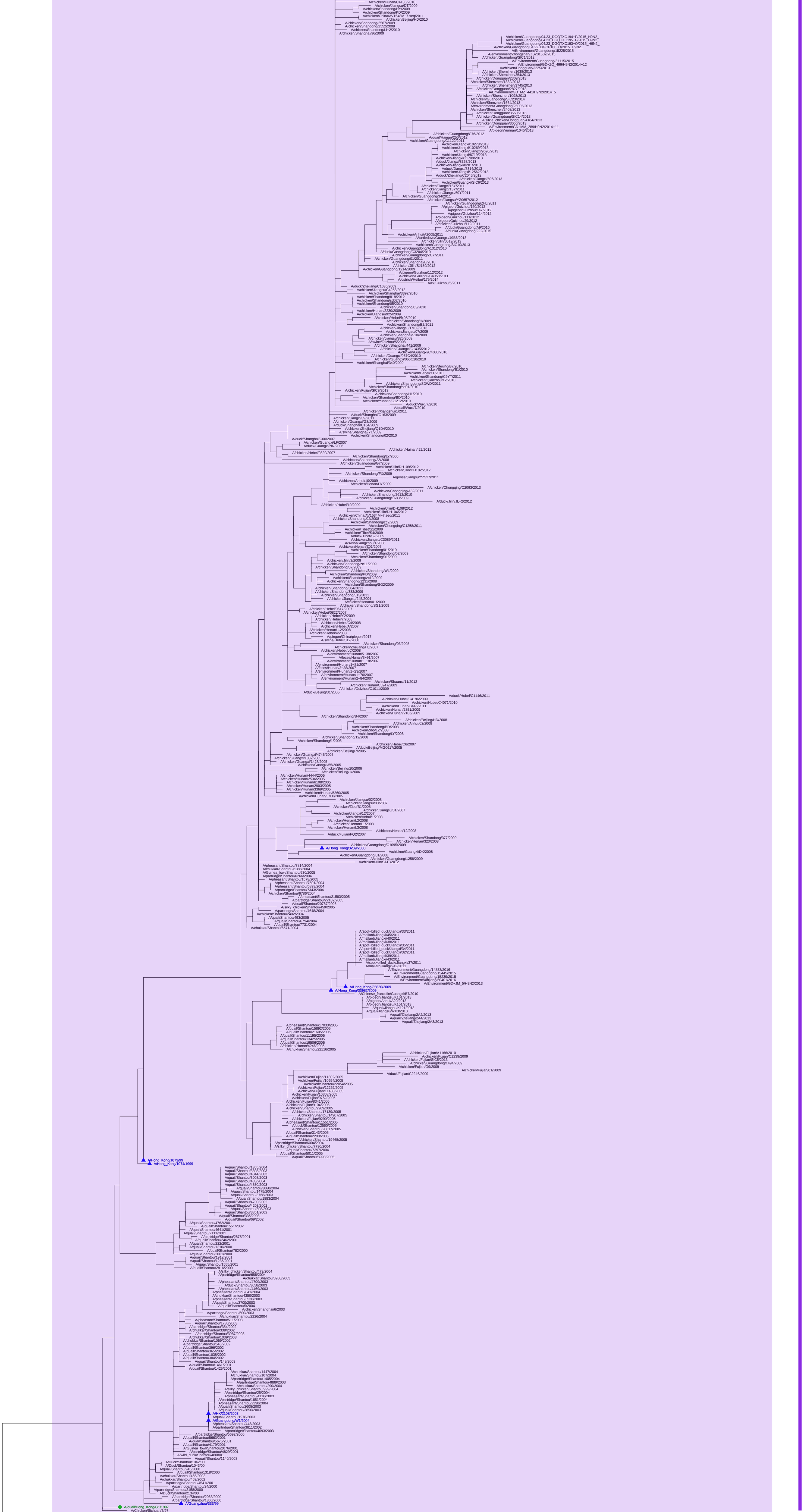

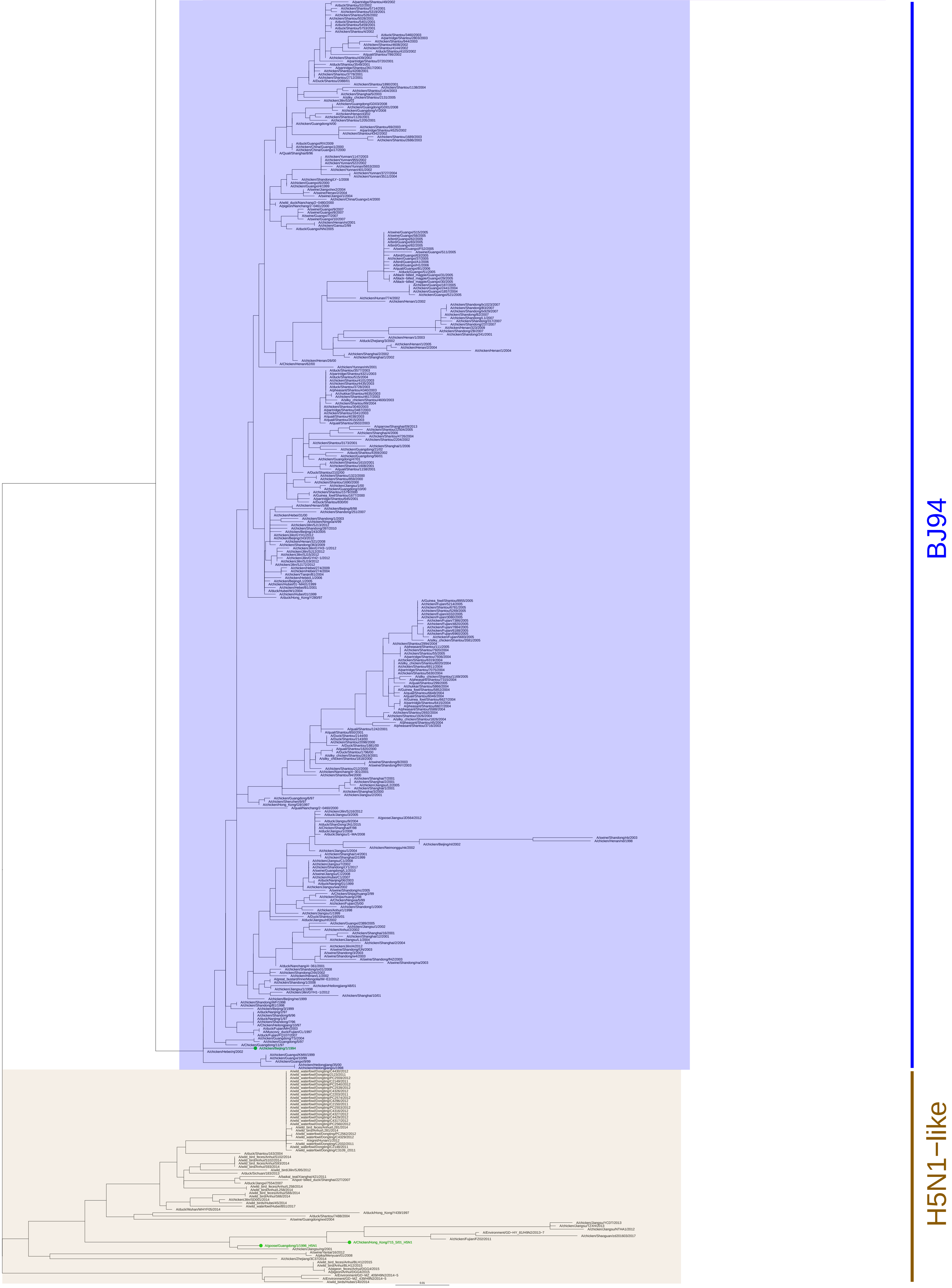
BJ94
H5N1−like

(H) NS

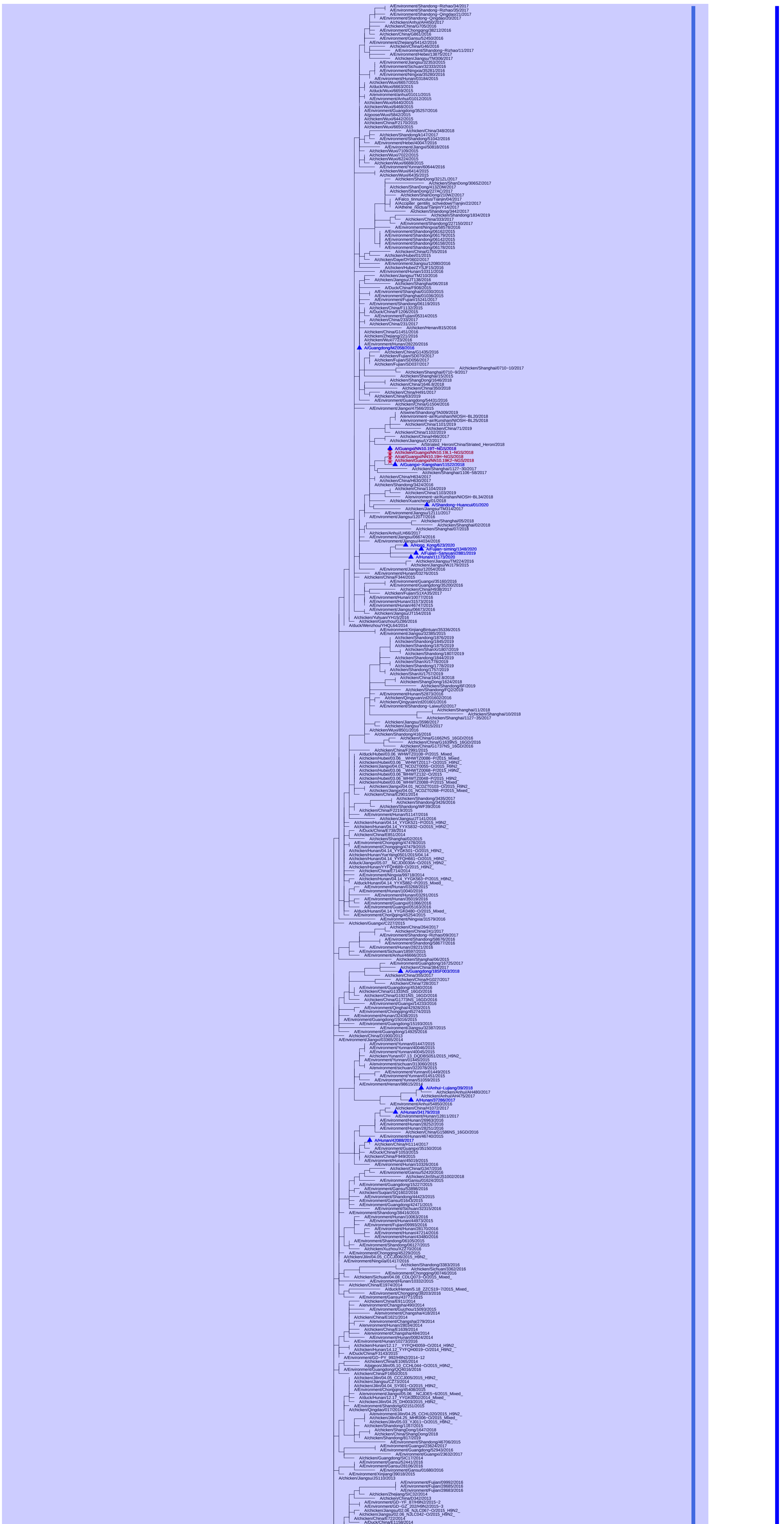

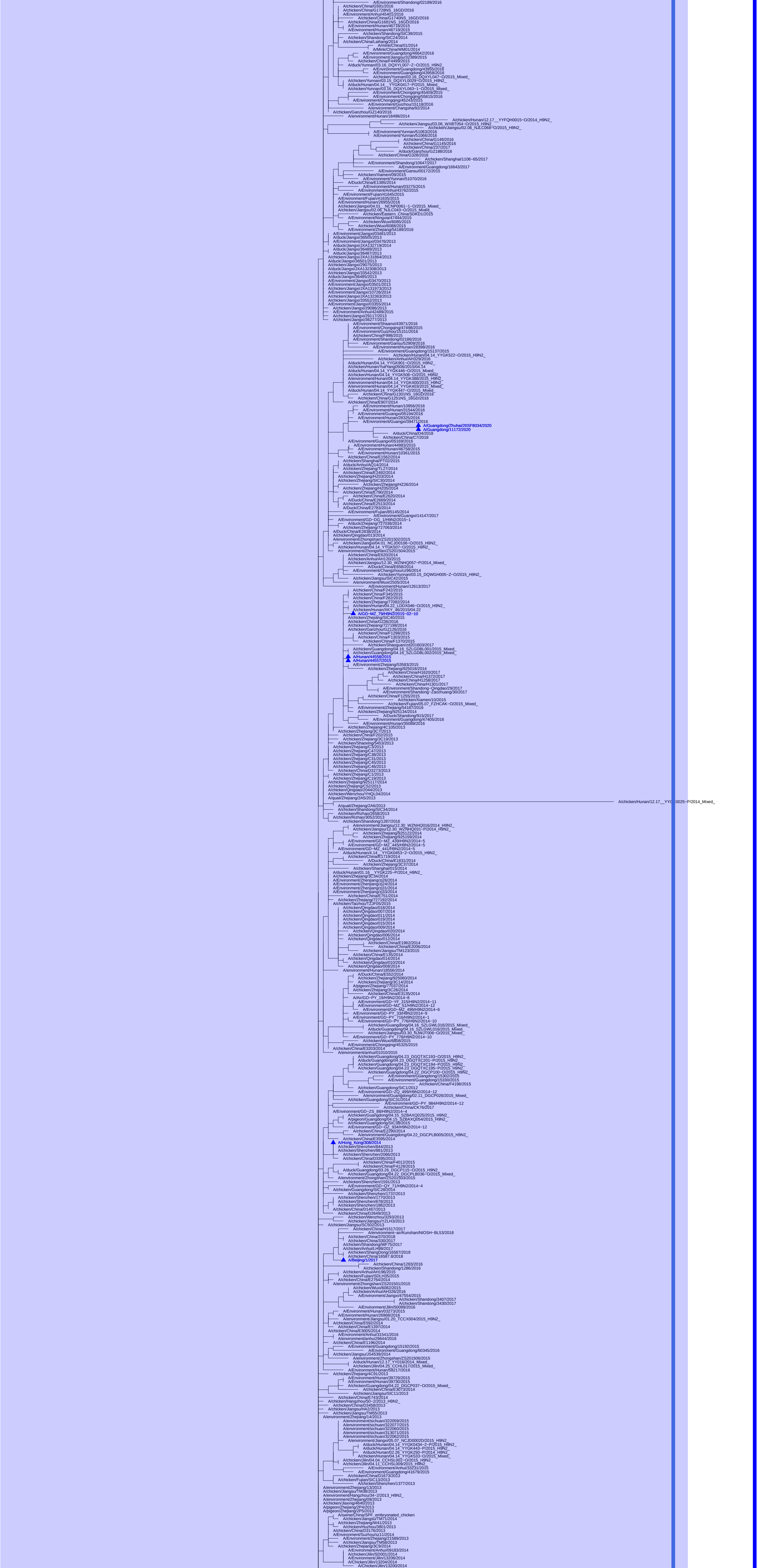

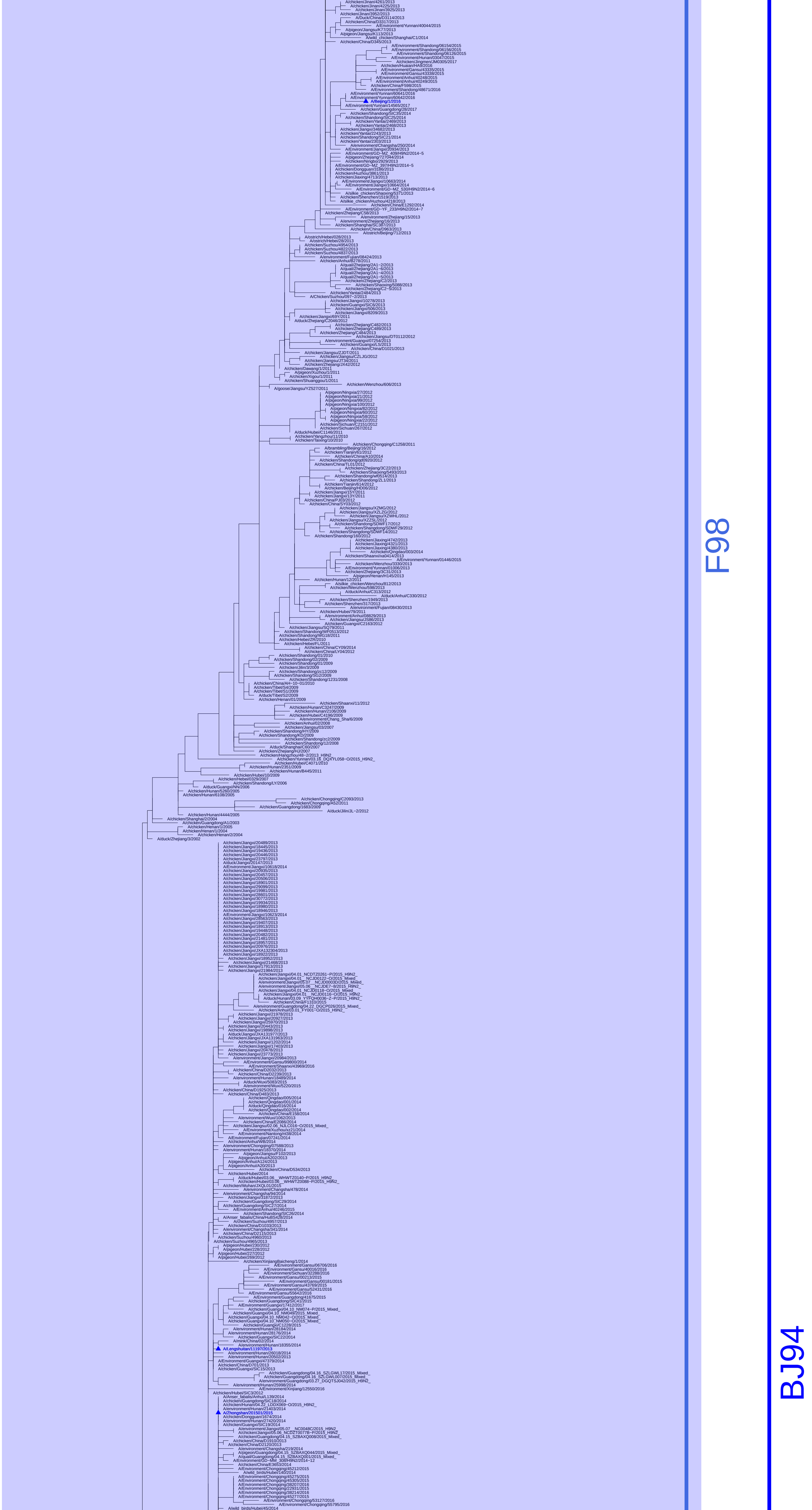
F98
BJ94

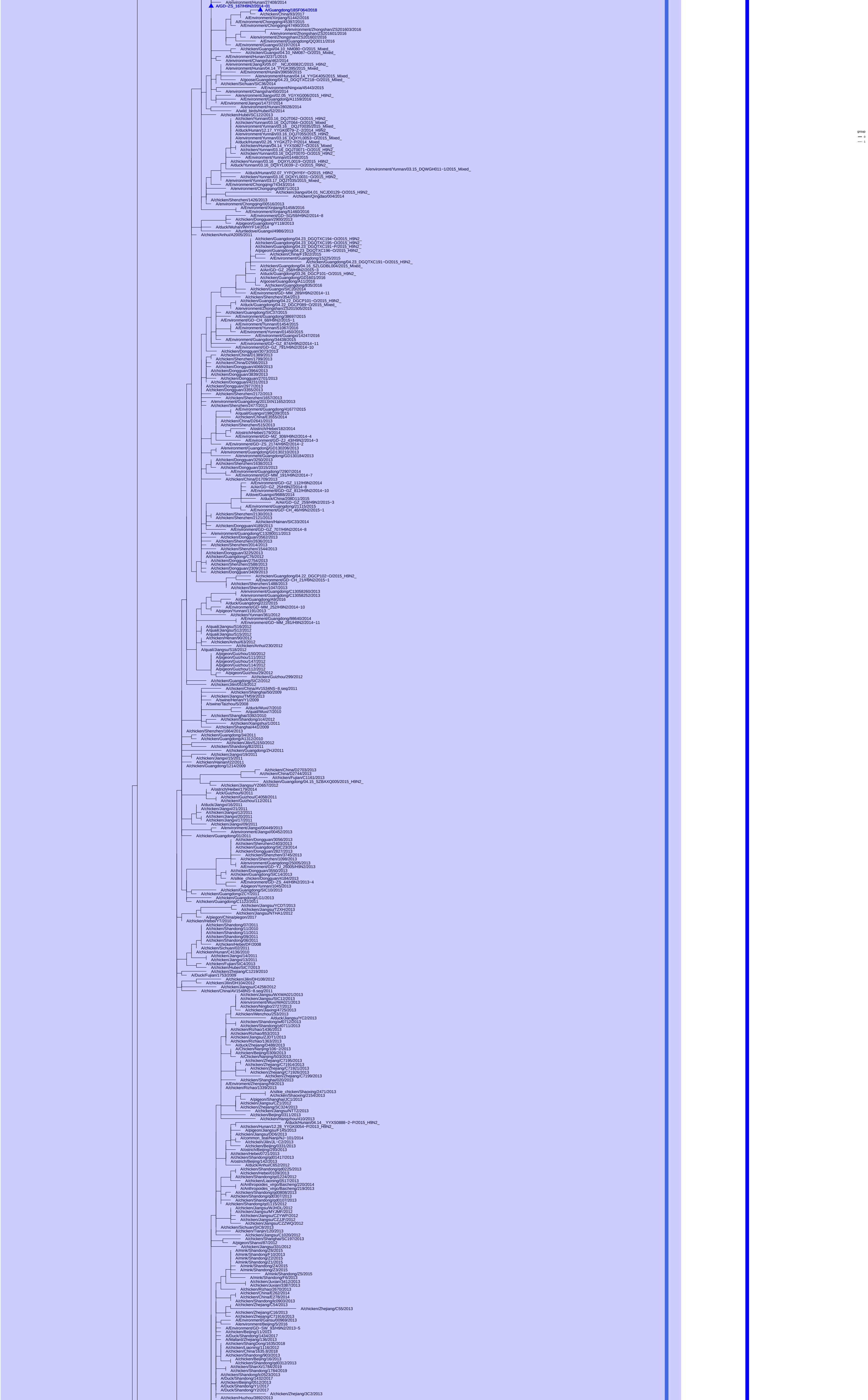

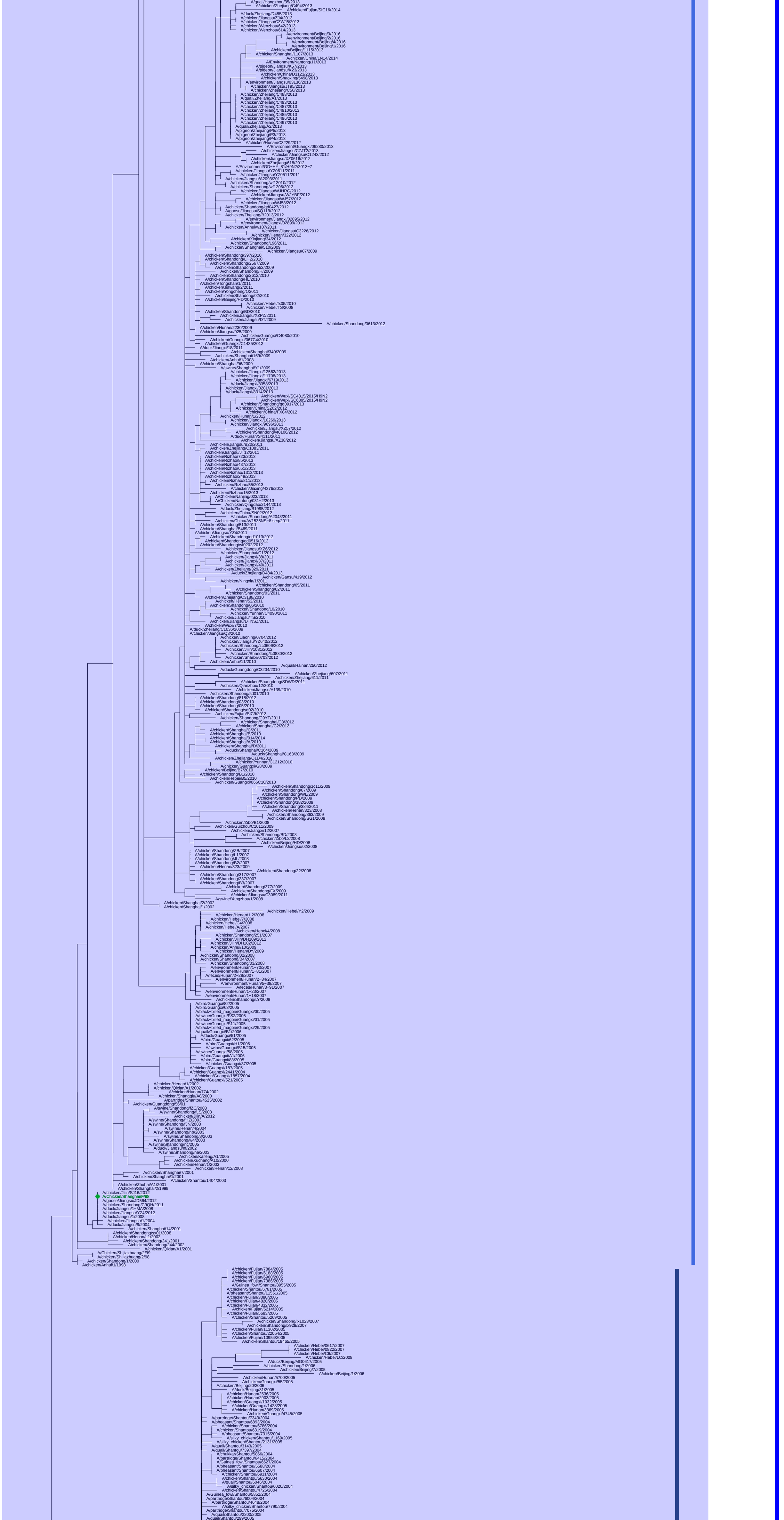

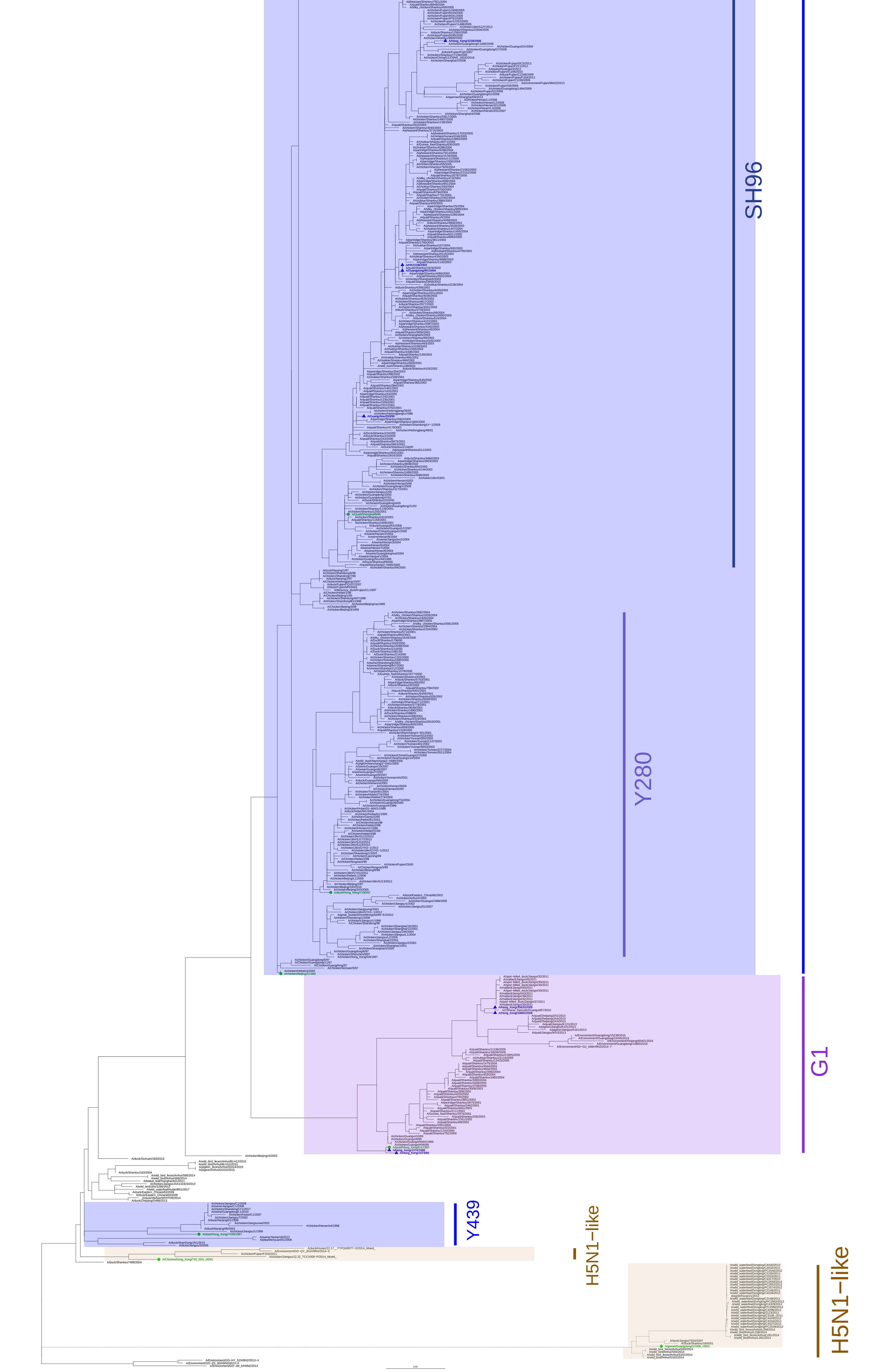
SH96
Y280
G1
Y439
H5N1-like
H5N1-like
